# Circular RNA TFRC/*SCD1* mRNA interaction regulates ferroptosis and metastasis in gastric cancer

**DOI:** 10.1038/s41419-025-07759-x

**Published:** 2025-06-05

**Authors:** Zhi Lin, Chonglei Zhong, Ming Shi, Qinpeng Long, Liang Jing, Yang Yu, Jing Chou, Miao Chen, Minhuan Lan, Fei Long

**Affiliations:** 1 Department of Pediatrics, The Third Xiangya Hospital of Central South University, Changsha, Hunan China; 2 Department of Gastrointestinal Surgery, The Third Xiangya Hospital of Central South University, Changsha, Hunan China; 3 Department of Pediatrics, The First Affiliated Hospital of University of South China, Hengyang, Hunan China; 4 Department of Gastrointestional Surgery, Hunan University of Medicine General Hospital, Huaihua, Hunan China; 5 Department of Gastrointestinal Surgery, The First Affiliated Hospital of Guangzhou University of Traditional Chinese Medicine, Guangzhou, Guangdong China; 6 College of Chemistry and Chemical Engineering, Central South University, Changsha, Hunan China; 7 Postdoctoral Research Station of Basic Medicine, The Third Xiangya Hospital of Central South University, Changsha, China

**Keywords:** Gastric cancer, Cell death

## Abstract

Ferroptosis, an iron-dependent form of programmed cell death, holds promise for cancer treatment. Circular RNAs (circRNAs), widely expressed across tumor types, modulate multiple cellular biological processes, including ferroptosis. However, the regulatory dynamics of circRNAs in gastric cancer (GC)-associated ferroptosis remain poorly understood. Here, circTFRC (circBase ID: hsa_circ_0068606), a novel circRNA, was identified as significantly upregulated in GC tissues and cell lines, with its plasma levels strongly associated with tumor size and metastatic status. Targeted suppression of circTFRC enhanced ferroptotic cell death, resulting in reduced proliferation and motility of GC cells in vitro. At the molecular level, circTFRC bound directly to *SCD1* mRNAs, stabilizing and enhancing their translation via recruiting the RNA-binding protein ELAVL1. Elevated SCD1 expression mitigated ferroptosis and promoted oncogenic lipid metabolic reprogramming, thereby driving GC progression. In vivo studies further confirmed that circTFRC silencing promoted ferroptosis and inhibited tumor growth and progression. These results delineate a circTFRC-mediated axis that impairs ferroptosis vulnerability in GC cells and supports malignancy advancement. CircTFRC emerges as a biomarker with diagnostic potential and a candidate for therapeutic intervention targeting ferroptosis in GC.

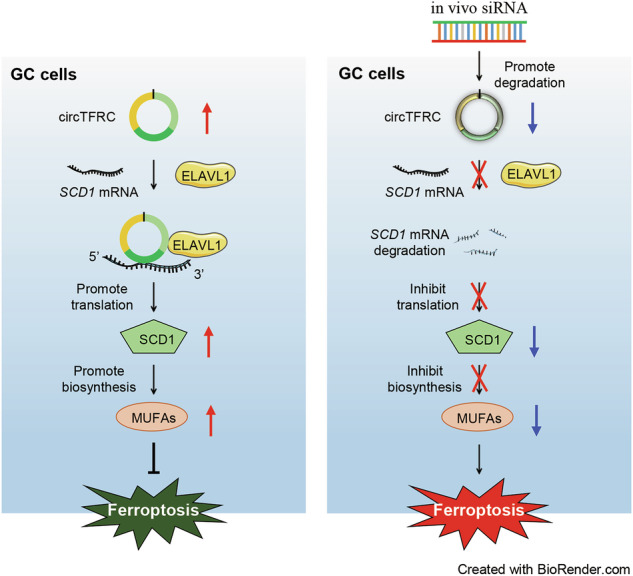

## Background

Gastric cancer (GC) remains a leading cause of cancer incidence and mortality globally, accounting for 4.9% of newly diagnosed malignancies and 6.8% of cancer-related deaths [1]. A substantial proportion of GC cases exhibit resistance to standard therapeutic modalities—including chemotherapy, radiotherapy, targeted agents, and immunotherapeutic strategies—culminating in elevated recurrence and metastasis rates, alongside unfavorable clinical outcomes [2, 3]. Accordingly, advancing the identification of novel molecular targets and the development of alternative therapeutic strategies is imperative for achieving precision oncology in GC.

Ferroptosis, a form of regulated cell death driven by iron accumulation, lipid peroxidation, and membrane disruption, has emerged as a significant factor in cancer biology over the past decade [4, 5]. Its role in tumor initiation and therapeutic response suggests considerable potential for targeted pharmacological modulation [6, 7]. In the context of GC, impaired ferroptosis regulation accelerates disease progression and is increasingly being recognized as a therapeutic opportunity. Ferroptosis-related proteins, such as ACTL6A [8], SOX13 [9], and HTR2B [10], have been identified as viable intervention points, reinforcing the therapeutic relevance of ferroptosis-oriented approaches in GC treatment development.

Circular RNAs (circRNAs), characterized by covalently closed loop structures, represent a class of abundant, stable, evolutionarily conserved, and tissue-specific noncoding RNAs (ncRNAs) [11]. Aberrations in circRNA expression across tumor types have been implicated in oncogenesis through mechanisms such as miRNA sequestration, protein interaction modulation, and serving as templates for protein translation [12, 13]. Recent studies have implicated circRNA dysregulation in the modulation of ferroptosis across malignancies. For example, circPIAS1 (circBase ID: hsa_circ_0007088) promotes hepatocellular carcinoma progression by inhibiting ferroptosis via the miR-455-3p/NUPR1/FTH1 axis [14], whereas circFOXP1 encodes a protein isoform (circFOXP1-231aa) that activates ferroptosis pathways to prevent recurrence in intrahepatic cholangiocarcinoma (ICC) [15]. Furthermore, circRNAs transported via exosomes, such as circUPF2 [16] and circRNA_101093 [17], contribute to ferroptosis resistance in cancer cells. Nevertheless, the regulatory mechanisms of circRNAs in GC-associated ferroptosis and the underlying molecular circuitry remain largely unknown [18, 19].

This study identified that ferroptosis-associated circRNA circTFRC (circBase ID: hsa_circ_0068606) was markedly upregulated in GC. Mechanistically, circTFRC bound directly to stearoyl coenzyme A desaturase-1 (*SCD1*) mRNAs, enhancing their stability and translation through an ELAVL1-dependent mechanism, thereby conferring resistance to ferroptosis and promoting GC cell proliferation and motility. In vivo, silencing circTFRC with small interfering RNAs (siRNAs) effectively triggered ferroptosis and suppressed tumor progression in murine models. Targeting circTFRC with siRNAs for ferroptosis induction may offer a viable therapeutic strategy for GC management.

## Methodologies and materials

### Patients and samples

Primary GC tissues and matched adjacent histologically normal tissues (located >5 cm from the lesion) were collected from 42 patients undergoing gastrectomy for GC without preceding neoadjuvant therapy at The Third Xiangya Hospital of Central South University between January and December 2023. Additionally, three normal gastric mucosal samples were acquired from healthy individuals undergoing diagnostic gastroscopy. Peripheral blood samples were obtained from 40 GC patients and 20 age- and sex-matched healthy controls. All specimens were promptly snap-frozen in liquid nitrogen and stored at −80 °C. Ethical approval was granted by the Ethics Committee of The Third Xiangya Hospital of Central South University (No: 2023-S371), and informed consent was obtained from all participants.

### Cell lines and cell culture

Human gastric epithelial cell line GES-1 and GC cell lines AGS, HGC-27, MKN-45, and MKN-28 were obtained from the American Type Culture Collection (ATCC). Cells were cultured in RPMI 1640 medium (Gibco) supplemented with 10% FBS (Sigma-Aldrich), 100 IU/ml penicillin, and 100 mg/ml streptomycin under standard conditions (37 °C, 5% CO_2_). Cell line identity was confirmed via short tandem repeat (STR) profiling, accompanied by assessments of isozyme expression, viability, and mycoplasma contamination.

### CircRNA microarray analysis

CircRNA microarray datasets GSE194384 (three paired GC and adjacent non-tumorous tissues), GSE93541 (plasma samples from three GC patients and three healthy individuals), and GSE83521 (six GC and six corresponding non-tumorous mucosal tissues) were retrieved from the Gene Expression Omnibus (GEO) database. CircRNA expression profiling was performed using the Arraystar Human CircRNA microarray V1 (GSE93541, GSE83521) and V2 (GSE194384) on GPL19978 and GPL21825 platforms, respectively. Bioinformatics analysis identified differentially expressed circRNAs between GC and normal samples (*P* < 0.05), with a selection criterion of |log_2_FC| ≥ 1 to define significantly altered circRNAs in GC tissues or plasma.

### Bioinformatics analysis

The GEO (https://www.ncbi.nlm.nih.gov/geo/) and GC cohort datasets from The Cancer Genome Atlas (TCGA) (https://www.cancer.gov/ccg/research/genome-sequencing/tcga) were employed to compare gene expression profiles between normal and tumor tissues and to explore gene co-expression patterns. CircRNA identification and annotation were conducted using circBase (http://www.circbase.org/), circBank (http://www.circbank.cn/), and circRNADb (http://reprod.njmu.edu.cn/cgi-bin/circrnadb/circRNADb.php). Predictions of RNA–RNA-binding protein (RBP) interactions were performed via ENCORI (https://rnasysu.com/encori/), RBPDB (http://rbpdb.ccbr.utoronto.ca/), CircInteractome (https://circinteractome.nia.nih.gov/), and RNA–Protein Interaction Prediction (RPISeq) (http://pridb.gdcb.iastate.edu/RPISeq/). The FerrDb V2 platform (http://www.zhounan.org/ferrdb/current/) was applied to screen for genes implicated in ferroptosis inhibition. Complementarity between circRNAs and mRNAs was assessed using the Basic Local Alignment Search Tool (BLAST) (http://blast.ncbi.nlm.nih.gov/) to identify potential high-affinity base-pair interactions.

### Total RNA extraction and quantitative real-time PCR (qRT‒PCR)

Total RNA was extracted using TRIzol Reagent (Invitrogen) and reverse-transcribed with RiverTra Ace qPCR RT Master Mix containing gDNA Scavenger (TOYOBO), according to the manufacturer’s instructions. Quantitative analysis of gene expression was conducted on the LightCycler 480 Real-Time PCR System (Roche) with the KOD SYBR^®^ qPCR Mix Kit (TOYOBO). Relative expression levels of circRNAs and mRNAs were determined via the 2^−ΔΔCt^ method, with *GAPDH* serving as the internal reference. Primer sequences were provided in Table S1.

### RNase R treatment assays

Total RNA (2 μg) extracted from AGS and HGC-27 cells underwent RNase R (5 U/μg, Epicenter Technologies) treatment at 37 °C for 30 min to assess RNA stability, followed by reverse transcription. Quantification of circTFRC, *TFRC* mRNA, and *GAPDH* mRNA levels was performed via qRT–PCR.

### Actinomycin D treatment assays

AGS and HGC-27 cells were seeded into six-well plates and treated with 5 μg/mL Actinomycin D (Sigma-Aldrich) or DMSO upon reaching ~60% confluency following a 24-h incubation. Total RNA was harvested at specified time points (0, 2, 4, 6, 8, 12, and 24 h) for qRT–PCR analysis to quantify circTFRC and linear *TFRC* mRNA levels, enabling calculation of their respective transcript half-lives.

### Nuclear and cytoplasmic RNA extraction

Cytoplasmic and nuclear RNA isolation was performed using the Cytoplasmic & Nuclear RNA Purification Kit (Norgen Biotek) according to the manufacturer’s instructions. AGS or HGC-27 cells were lysed in pre-chilled Lysis Buffer J for 5 min on ice. Lysates were then centrifuged at maximum speed for 3 min using a benchtop centrifuge, resulting in a cytoplasmic fraction in the supernatant and a nuclear fraction in the pellet. RNA from both compartments was column-bound, subjected to sequential washes with Wash Solution A, and subsequently eluted for downstream analysis.

### RNA fluorescence in situ hybridization (FISH)

Cy3-conjugated FISH probes specific to the back-splice junction (BSJ) of circTFRC were synthesized by GenePharma. RNA FISH was conducted using the GenePharma RNA FISH Kit in accordance with the manufacturer’s instructions [20]. Fluorescence imaging was performed on a LEICA^®^ STELLARIS 5 Confocal Microscope Platform (Leica Microsystems, Germany).

### Small interfering RNAs (siRNAs), plasmid, short hairpin RNAs (shRNAs), lentivirus construction, and cell transfection

Two siRNAs targeting circTFRC BSJ regions and a negative control siRNA (si-NC) were synthesized by GenePharma. ELAVL1 and *SCD1* overexpression vectors (GV367) were obtained from GeneChem. GC cell transfection with the respective siRNAs or plasmids was conducted using Lipofectamine 3000 (Invitrogen) according to the manufacturer’s instructions. For the generation of stable knockdown cell lines, lentiviral particles encoding circTFRC-targeting shRNAs, along with green fluorescent protein and puromycin resistance cassettes, were provided by GeneChem. Seventy-two hours post-infection, puromycin (Gibco) was applied at 5–10 μg/mL to enrich for successfully transduced cells exhibiting circTFRC silencing. Silencing efficiency was verified by qRT–PCR. The siRNA target sequences were as follows: si-circTFRC#1, GTCATGAGAGTTCTTCTGTGT-dTdT; si-circTFRC#2, GATCGTGTCATGAGAGTTCTT-dTdT.

### Cell counting kit-8 (CCK-8) assay

Cell viability was assessed using the CCK-8 assay. Cells (2000 per well) were seeded into 96-well plates and incubated for 0, 24, 48, or 72 h. Subsequently, 10 μL of CCK-8 reagent (Abbkine) was added to each well and incubated for 1 h. Absorbance at 450 nm was measured using a BioTek microplate reader (ELX800, BioTek instruments).

### Plate colony formation assay

Plate colony formation assays were employed to evaluate cellular proliferative capacity. A total of 200–500 treated cells per group were seeded into 6-well plates in triplicate. After a 7–10-day incubation period, cells were fixed with 4% paraformaldehyde for 30 min, stained with 0.1% crystal violet for 15 min, and subsequently air-dried at room temperature. Colonies containing at least 50 cells were imaged and quantified.

### Transwell assay

Cell motility was evaluated using 24-well Transwell chambers with 8 μm pore inserts (Corning). Approximately 2–6 × 10⁴ treated cells were suspended in 200 μL of serum-free medium and seeded into the upper compartment, while 600 μL of complete medium (RPMI 1640 supplemented with 10% FBS) was added to the lower compartment as a chemoattractant. After a 24-h incubation, non-migratory cells remaining on the upper membrane surface were removed. Cells that traversed to the lower membrane surface were fixed in 4% paraformaldehyde for 30 min, stained with 0.1% crystal violet for 15 min at room temperature, and quantified across five randomly selected fields under an Olympus BX51 inverted microscope at ×100 magnification.

### RSL3 and ferrostatin-1 treatment

RSL3, a selective glutathione peroxidase 4 (GPX4) inhibitor, induces ferroptosis by triggering lipid peroxide accumulation and subsequent cell demise [21]. Its potent activity across diverse cell types, including GC cells, has established it as a standard reagent in ferroptosis studies [9, 22]. Conversely, ferrostatin-1 (Fer-1), a lipid ROS scavenger, impedes ferroptosis progression by stabilizing cellular membranes and suppressing lipid peroxidation, without interfering with other cell death modalities [8, 9, 23]. The combination of RSL3 and Fer-1 enables precise modulation of ferroptosis responses in GC cells, thereby facilitating mechanistic investigations and informing therapeutic development. Accordingly, RSL3 was employed to initiate ferroptosis, while Fer-1 was applied to counteract ferroptosis damage in this study.

GC cells were plated at an optimized density and incubated for 24 h prior to treatment. The culture medium was then refreshed with formulations containing RSL3 (1 µM), Fer-1 (0.75 µM), or both agents in combination, followed by a 16-h incubation period. Post-treatment assessments included quantification of cell death, reactive oxygen species (ROS) accumulation, and lipid peroxidation, utilizing the respective detection assays described below.

### Cell death assay

To evaluate cell death rates, ~3 × 10⁵ GC cells were seeded in 6-well plates and treated the next day as specified. Following treatment, cells were incubated with propidium iodide (PI) for 20 min at 37 °C, then washed twice with PBS. Fluorescence microscopy (×100 magnification) was employed to assess morphological alterations and calculate the proportion of PI-positive (dead) cells. Quantitative analysis of PI-positive populations was subsequently conducted using the Cytek^®^ Northern Lights™-CLC (NL-CLC) full spectrum flow cytometry system (Cytek^®^ Biosciences, USA), with a minimum of 10,000 cells analyzed per condition. FlowJo™ V10.8 Software was utilized for downstream data processing.

### Lipid ROS detection

Lipid ROS were quantified using the C11 BODIPY 581/591 probe (Thermo Fisher Scientific) following established protocols [24]. Treated cells were incubated with 5 µM C11 BODIPY 581/591 at 37 °C for 30 min, then washed three times with PBS. Oxidation of the probe induced a fluorescence shift from ~590 to ~510 nm, corresponding to lipid ROS accumulation. This shift was measured on a Cytek^®^ NL-CLC full spectrum flow cytometry system (Cytek^®^ Biosciences, USA), analyzing a minimum of 10,000 cells per sample. Data were processed using FlowJo™ V10.8 Software. For confocal imaging, cells were cultured in confocal dishes (Biosharp, China), stained with C11 BODIPY 581/591 post-treatment, washed, and imaged with the LEICA^®^ STELLARIS 5 Confocal Microscope Platform (Leica Microsystems, Germany).

### Malondialdehyde (MDA) detection

MDA concentrations in both experimental cells and tumor tissues were quantified using a colorimetric assay kit (Elabscience) according to the manufacturer’s protocol. Cell lysis was conducted on ice with MDA Lysis Buffer containing BHT, followed by centrifugation at 13,000 × *g* for 10 min. Supernatants were transferred to microcentrifuge tubes, and TBA solution was added to enable formation of the MDA-TBA adduct. The resulting mixtures were incubated at 95 °C for 70 min and subsequently cooled to ambient temperature. A 200 μL aliquot of each reaction was dispensed into a 96-well plate, and absorbance was recorded at 532 nm.

### Measurement of intracellular ROS

Intracellular ROS levels were quantified by incubating treated cells with 5 µM CM-H2DCFDA (Glpbio, USA), a redox-sensitive fluorescent probe, for 30 min at 37 °C in accordance with the manufacturer’s protocol. Post-incubation, unbound dye was removed through washing, and fluorescence intensity was recorded using the Cytek^®^ NL-CLC full spectrum flow cytometry system (Cytek^®^ Biosciences). A minimum of 20,000 cells was evaluated for each condition. Subsequent data processing was conducted with FlowJo™ V10.8 Software.

### Measurement of mitochondrial ROS

Mitochondrial ROS levels were measured using MitoSOX Red (Glpbio, USA) via full-spectrum flow cytometry. Post-treatment, cells were incubated with 5 µM MitoSOX at 37 °C for 10 min, followed by analysis on the Cytek^®^ NL-CLC system (Cytek^®^ Biosciences, USA). A minimum of 20,000 cells was evaluated for each condition. Data processing was conducted with FlowJo™ V10.8 Software.

### Measurement of intracellular Fe^2+^

Intracellular Fe^2+^ levels in GC cells were quantified by incubating cells with 1 µM FerroOrange (F374, DOJINDO, Japan) for 30 min at 37 °C in the absence of light. FerroOrange, as a novel fluorescent probe, enables fluorescence imaging of Fe^2+^ within live cells. Fluorescence signals were then captured using a confocal microscope (STELLARIS, Leica, Germany).

### Western blotting (WB) and antibodies

Cells were lysed on ice for 15 min in 1× cell lysis buffer (Cell Signaling Technology) supplemented with 1× protease and phosphatase inhibitor cocktail (Cell Signaling Technology). Lysates were centrifuged at 12,000 rpm for 15 min at 4 °C, and supernatants were collected for protein quantification using the bicinchoninic acid assay (Thermo Fisher Scientific). Equal amounts of protein (20 µg) were resolved on 10% or 12% polyacrylamide gels and transferred to 0.2 μm polyvinylidene difluoride membranes (Millipore). Membranes were blocked in TBST containing 8% skim milk for 1.5 h, followed by overnight incubation at 4 °C with primary antibodies. After washing, membranes were exposed to HRP-conjugated secondary antibodies for 1 h at room temperature, and signal development was performed using enhanced chemiluminescence (Thermo Fisher Scientific). Imaging and densitometric analysis were conducted using the ChemiDoc Touch Imaging System and Image Lab Software (Bio-Rad). Antibodies included ELAVL1 (Proteintech, #11910-1-AP; 1:2000), SCD1 (Proteintech, #28678-1-AP; 1:2000), GPX4 (ABclonal, A11243; 1:1000), and β-actin (CST, 3700S; 1:5000).

### RNA-binding protein immunoprecipitation (RIP)

RIP assays were performed using the EZ-Magna RNA-Binding Protein Immunoprecipitation Kit (Merck, KGaA) in accordance with the manufacturer’s instructions [20]. Briefly, protein A/G magnetic beads were pre-incubated with target-specific antibodies (e.g., anti-ELAVL1) or anti-IgG as a negative control. Cell lysates were prepared in RIP lysis buffer supplemented with protease and RNase inhibitors, followed by overnight incubation at 4 °C with the antibody-conjugated beads. After six stringent wash steps, immunoprecipitated complexes were further digested with RNase-free DNase and proteinase K for RNA purification. The isolated RNA was quantified by qRT–PCR, and enrichment was calculated relative to input RNA.

### RNA pull-down assay

Biotin-labeled probes targeting circTFRC and corresponding controls were custom-synthesized by Sangon Biotech for RNA pull-down assays. Experimental procedures followed the Pierce™ Magnetic RNA–Protein Pull-Down Kit protocol (Pierce Biotechnology) [20]. Approximately 1 × 10^7^ cells were washed with PBS and lysed in lysis buffer. For probe-bead complex formation, 3 µg of biotinylated circTFRC-specific or control probes were incubated with 50 µL of prewashed streptavidin magnetic beads at room temperature for 1 h. The resulting complexes were then incubated with cell lysates overnight at 4 °C under constant rotation. After six washes with the supplied buffer, bound components were eluted in 50 µL of elution buffer at 37 °C for 30 min with agitation. Isolated RNAs were subjected to qRT-PCR, and associated proteins were analyzed via Western blotting.

### Animal experiments

Female NOD-SCID mice (6 weeks old, 18–20 g) were obtained from SLAC Laboratory Animal Co., Ltd (Hunan, China) and housed in the Department of Laboratory Animals, Central South University (Hunan, China). Animals were maintained in sterile individually ventilated cages (IVCs) under specific pathogen-free conditions, with ad libitum access to sterilized food and water. For xenograft establishment, 5 × 10⁶ GC cells were subcutaneously implanted into each mouse. Tumor development was assessed every 3 days, alongside concurrent monitoring of tumor volume and body weight. Tumor volume was calculated using the formula: 0.5 × length × width². Once tumors reached ~50 mm³, mice were randomly allocated into two groups (*n* = 7 per group): (1) control siRNA and (2) circTFRC siRNA. The treatment group received intratumoral injections of in vivo-optimized circTFRC siRNA (5 nmol/injection, RiboBio) every 3 days, while the control group was administered control siRNA [25, 26]. After 21 days of treatment, mice were euthanized and tumors harvested for further analysis.

For metastasis modeling, 3 × 10⁶ GC cells expressing firefly luciferase were introduced into recipient mice via tail vein injection to enable in vivo tracking. After 1-week engraftment period, mice were randomly assigned to two cohorts (*n* = 7 per group): a control siRNA group and a circTFRC siRNA group. The latter received intravenous administration of in vivo-optimized circTFRC siRNA (10 nmol per dose, RiboBio) every 3 days, whereas the control cohort was treated with non-targeting siRNA [27, 28]. Bioluminescent imaging using the IVIS^®^ Spectrum system (PerkinElmer) was conducted weekly to assess tumor dissemination, with 150 mg/kg D-luciferin (YEASEN, 40902ES03) administered intraperitoneally 10 min prior to imaging. Animal body weights were recorded weekly, and general condition was evaluated every 3 days. At 6 weeks post-injection, mice were euthanized under humane protocols, and lung tissues were harvested for further analysis. All procedures involving animals received ethical approval from the Department of Laboratory Animals, Central South University (Changsha, Hunan, China) and complied with the National Institutes of Health Guide for the Care and Use of Laboratory Animals.

### Statistical analysis

All non-animal experiments were independently conducted at least three times, with representative results presented. Data are expressed as mean ± standard deviation (SD) based on a minimum of three biological replicates. Statistical differences were determined using either Student’s *t* test or one-way analysis of variance (ANOVA), as indicated in the corresponding figure legends. Diagnostic performance of circTFRC as a GC biomarker was assessed via receiver operating characteristic (ROC) curve analysis, including calculation of area under the curve (AUC) values. All statistical analyses were performed using GraphPad Prism version 8.0.1. Significance thresholds were annotated in figures as follows: * *P* < 0.05, ** *P* < 0.01, *** *P* < 0.001, **** *P* < 0.0001.

## Results

### CircTFRC exhibited elevated expression in GC and was linked to GC advancement

**Fig. 1:**
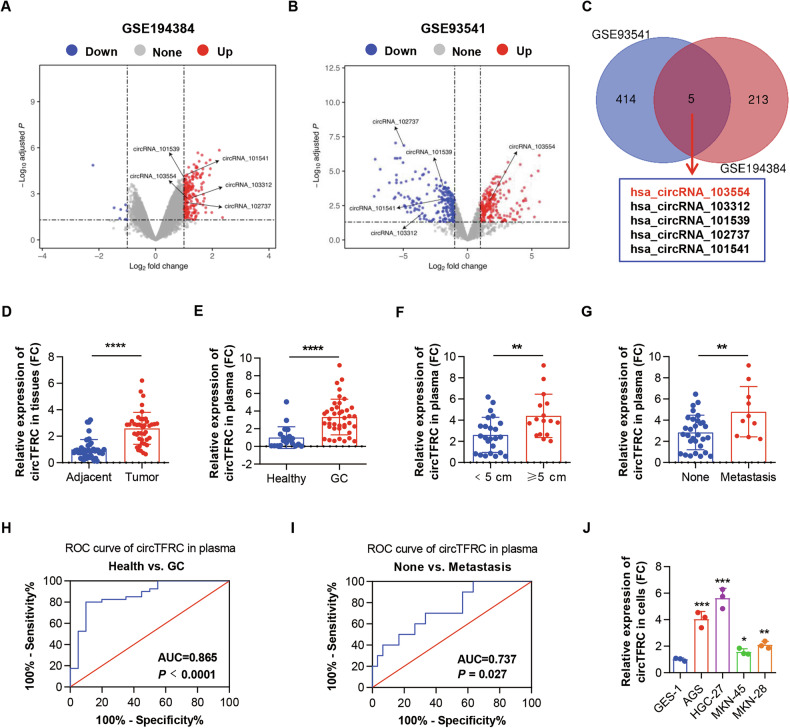
CircTFRC exhibited elevated expression in GC and was linked to GC advancement. **A** Volcano plot of differentially expressed circRNAs in three pairs of GC tissues and adjacent normal-appearing tissues. **B** Volcano plot of circRNA expression in plasma samples from three patients with GC and three healthy controls. Screening criteria: |log_2_FC| ≥ 1, *P* < 0.05. Red points indicate significantly upregulated circRNAs; blue points indicate significantly downregulated circRNAs. **C** Venn diagram displaying intersecting differentially expressed circRNAs between the two microarray datasets. **D** qRT–PCR analysis of circTFRC expression in 42 pairs of GC tissues and adjacent normal-appearing tissues. **E** qRT–PCR analysis of circTFRC expression in plasma from 40 patients with GC and 20 healthy volunteers. **F** qRT–PCR analysis comparing circTFRC expression in plasma from 24 GC individuals with small tumors (<5 cm) and 16 GC individuals with large tumors (≥5 cm). **G** qRT–PCR analysis comparing circTFRC expression in plasma from 30 GC individuals without distant metastases and 10 GC individuals with distant metastases. **H** ROC curve evaluating the diagnostic accuracy of circTFRC in plasma samples for distinguishing patients with GC from healthy controls. **I** ROC curve evaluating the diagnostic accuracy of circTFRC in plasma samples for distinguishing GC individuals with distant metastases from those without. **J** qRT–PCR analysis of circTFRC expression in the gastric normal epithelial cell line (GES-1) and various GC cell lines. Data are presented as mean ± SD. *P* values were calculated using a two-tailed paired (**D**) or unpaired Student’s *t* test (**E**–**G**) or one-way ANOVA (**J**); *P < 0.05, **P < 0.01, ***P < 0.001, ****P < 0.0001. See also Fig. S1.

To investigate circRNA expression profiles in GC tissues and patient plasma, two microarray datasets (GSE194384 and GSE93541) were analyzed. A total of 218 and 419 circRNAs were identified as differentially expressed (|log_2_FC| ≥ 1.0, *P* < 0.05) in tissue and plasma samples, respectively (Fig. 1A, B; Tables S2–S3). Cross-comparison of the datasets revealed five overlapping circRNAs (Fig. 1C), among which hsa_circRNA_103554 (circTFRC) was the only one consistently and selectively upregulated in both GC tissues and plasma (Figs. 1A, B and S1A, B). Independent validation using the GSE83521 dataset confirmed elevated circTFRC expression in GC tissues (Fig. S1C). Comparative analysis of circTFRC expression between three normal-appearing tissues adjacent to GC and three matched normal gastric mucosal samples from healthy donors revealed no significant differences (Fig. S1D). Consequently, adjacent non-tumorous tissues were used as controls throughout the study. Quantitative RT–PCR analysis of 42 GC tissue pairs demonstrated increased circTFRC expression in tumor samples (Fig. 1D), while plasma from GC patients also exhibited significantly elevated levels relative to healthy individuals (Fig. 1E). Elevated plasma circTFRC levels were positively correlated with tumor size and distant metastasis (Fig. 1F, G). ROC analysis yielded an AUC of 0.865 (95% CI: 0.763–0.967, *P* < 0.0001), indicating robust discriminative power for distinguishing GC patients from healthy controls (Fig. 1H). Additionally, circTFRC levels differentiated GC cases with distant metastasis from those without, with an AUC of 0.737 (95% CI: 0.562–0.912, *P* = 0.027) (Fig. 1I), supporting its potential as a plasma-based biomarker for GC detection and stratification.

Additionally, elevated circTFRC expression was observed in GC cell lines (AGS, HGC-27, MKN-45, and MKN-28) compared to the normal gastric epithelial cell line GES-1 (Fig. 1J). Consistent with circRNA microarray data (Figs. S1A–C), the high abundance of circTFRC was further confirmed in GC cells, exceeding the expression levels of other reported upregulated circRNAs in GC, including circURI1 (Arraystar ID: hsa_circRNA_102503) [29], circNRIP1 (Arraystar ID: hsa_circRNA_103110) [30], circ-RanGAP1 (Arraystar ID: hsa_circRNA_103237) [31], hsa_circ_0088300 (Arraystar ID: hsa_circRNA_104902) [32], and hsa_circ_0007376 (Arraystar ID: hsa_circRNA_102415) [33] (Figs. S1E, F). Collectively, circTFRC demonstrates consistently elevated expression in GC and exhibits a strong association with tumor progression.

### CircTFRC represented a newly identified circRNA predominantly found in the cytoplasm

**Fig. 2:**
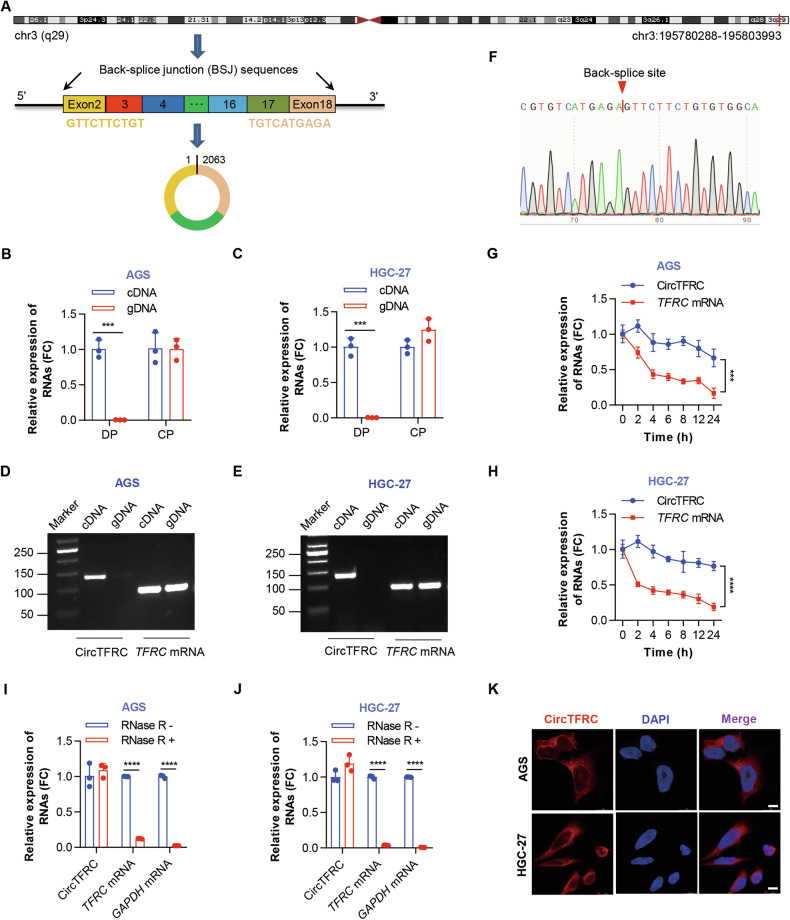
CircTFRC represented a newly identified circRNA predominantly found in the cytoplasm. **A** Schematic illustration of circTFRC’s chromosomal location, exon structure, and back-splice junction. **B**, **C** qRT–PCR results showing circTFRC amplification from cDNA and gDNA of GC cells using divergent (DP) and convergent primers (CP). cDNA complementary DNA, gDNA genomic DNA. **D**, **E** Agarose gel electrophoresis of PCR products verifying circTFRC circularization in GC cells. **F** Sanger sequencing confirming the back-splice junction of circTFRC. **G**, **H** qRT–PCR analysis of circTFRC and *TFRC* mRNA expression in GC cells treated with Actinomycin D (5 µg/mL) at various time points. **I**, **J** qRT–PCR analysis of circTFRC and *TFRC* mRNA expression in GC cells following RNase R treatment (5 U/μg, 30 min). **K** RNA fluorescence in situ hybridization (FISH) depicting the subcellular localization of circTFRC in GC cells. Nuclei are counterstained with DAPI. Scale bar, 10 µm. Data are presented as mean ± SD. *P*-values were calculated using a two-tailed unpaired Student’s *t* test (**B**, **C**, **I**, **J**) or two-way ANOVA (**G**, **H**); ***P < 0.001, ****P < 0.0001. See also Fig. S2.

According to circBase (ID: hsa_circ_0068606), circBank (ID: hsa_circTFRC_007), and circRNADb (ID: hsa_circ_06856), circTFRC is an exon-derived circRNA mapped to chr3:195780288–195803993, with a transcript length of 2063 nucleotides (nt). Based on GRCh37/hg19, it originates from exons 2–18 of the transferrin receptor (*TFRC*) locus (Fig. 2A) and is designated circTFRC. As circTFRC had not been previously annotated functionally, its existence and circular structure were experimentally validated. To eliminate the possibility of genomic rearrangement, both divergent primers specific to circTFRC and convergent primers targeting linear *TFRC* mRNA were employed (Fig. S2A, B). PCR amplification was detected only in cDNA using divergent primers, with no signal from genomic DNA, confirming the circular configuration of the *TFRC*-derived transcript and excluding trans-splicing artifacts (Fig. 2B–E). Sanger sequencing further verified the BSJ site of circTFRC (Fig. 2F), consistent with circBase and microarray probe annotations. Transcriptional inhibition with Actinomycin D revealed that circTFRC possessed a significantly extended half-life (>24 h) compared to linear *TFRC* mRNA (<4 h) (Fig. 2G–H), indicating greater transcript stability. RNase R treatment demonstrated resistance in circTFRC, whereas linear *TFRC* mRNA underwent substantial degradation (Fig. 2I, J). Subcellular localization by FISH (Fig. 2K) and nucleocytoplasmic fractionation (Fig. S2C, D) revealed predominant cytoplasmic enrichment of circTFRC in GC cells. Collectively, these results identify circTFRC as a highly stable, cytoplasm-localized circRNA derived from the TFRC gene in GC cells.

### CircTFRC knockdown inhibited the proliferation and migration of GC cells

**Fig. 3:**
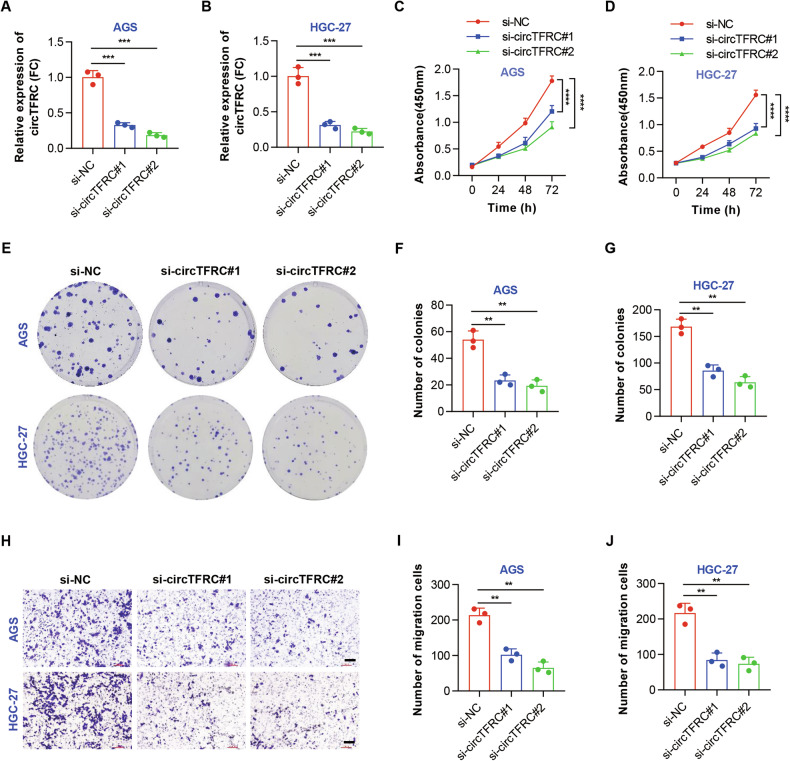
CircTFRC knockdown inhibited the proliferation and migration of GC cells. **A, B** qRT–PCR analysis confirming significant knockdown of circTFRC in AGS and HGC-27 cells following siRNA transfection. **C**, **D)** CCK-8 assay illustrating the proliferation of AGS and HGC-27 cells under control conditions (si-NC) or with circTFRC knockdown (si-circTFRC) at indicated time points (0, 24, 48, and 72 h). **E**, **F**, **G** Plate colony formation assay assessing colony formation in AGS and HGC-27 cells under control conditions (si-NC) or with circTFRC knockdown (si-circTFRC) over 7–10 days. **H**, **I**, **J** Transwell assay evaluating the migration ability of AGS and HGC-27 cells under control conditions (si-NC) or with circTFRC knockdown (si-circTFRC) over 24 h. Scale bar, 100 µm. Data are presented as mean ± SD. *P-*values were calculated using a two-tailed unpaired Student’s *t* test (**A**, **B**, **F**, **G**, **I**, **J**) or two-way ANOVA (**C**, **D**); **P < 0.01, ***P < 0.001, ****P < 0.0001. See also Fig. S3.

To examine the involvement of circTFRC in GC progression, two siRNAs targeting its BSJ site were designed to selectively suppress circTFRC expression in AGS and HGC-27 cells (Fig. 3A, B). CCK-8 (Fig. 3C, D) and colony formation assays (Fig. 3E–G) revealed a marked reduction in cell viability and proliferation following circTFRC knockdown. Transwell migration assays further showed diminished migratory capacity upon circTFRC silencing (Fig. 3H–J). Importantly, *TFRC* mRNA levels and other circRNAs originating from the *TFRC* locus, such as hsa_circ_0068631 [34, 35], remained largely unaffected by circTFRC depletion (Fig. S3A, B), suggesting that its influence on GC cell growth and motility are specifically mediated by circTFRC. Collectively, these results underscore the cancer-promoting function of circTFRC in GC.

### CircTFRC knockdown promoted the ferroptosis of GC cells

**Fig. 4:**
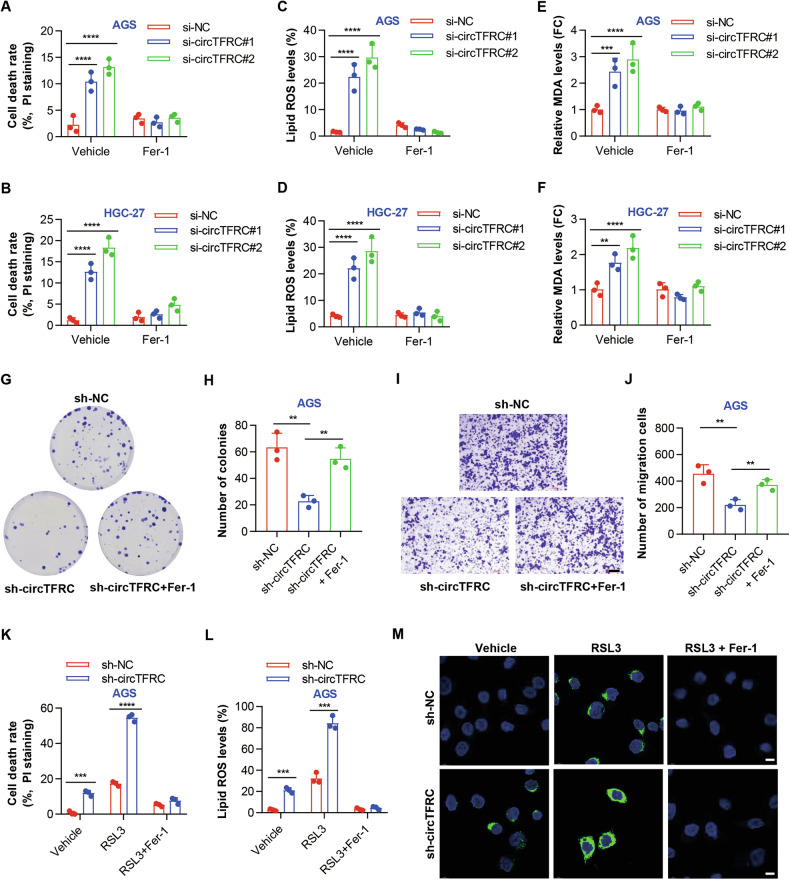
CircTFRC knockdown promoted the ferroptosis of GC cells. **A**, **B** Propidium iodide (PI) staining showing the cell death rates of control (si-NC) and circTFRC knockdown (si-circTFRC) AGS and HGC-27 cells in the absence or presence of ferrostatin-1 (0.75 μM, 16 h). **C**, **D** Flow cytometry showing the lipid ROS levels (stained with C11 BODIPY 581/591) in control (si-NC) and circTFRC knockdown (si-circTFRC) AGS and HGC-27 cells in the absence or presence of ferrostatin-1 (0.75 μM, 16 h). ROS reactive oxygen species. **E**, **F** ELISA assays showing the relative MDA levels in control (si-NC) and circTFRC knockdown (si-circTFRC) AGS and HGC-27 cells in the absence or presence of ferrostatin-1 (0.75 μM, 16 h). MDA malondialdehyde. **G**, **H** Plate colony formation assay assessing colony formation in AGS cells under control conditions (sh-NC) or with circTFRC knockdown (sh-circTFRC) in the absence or presence of ferrostatin-1 (0.25 μM, 72 h). **I**, **J** Transwell assay evaluating the migration ability of AGS cells under control conditions (sh-NC) or with circTFRC knockdown (sh-circTFRC) in the absence or presence of ferrostatin-1 (0.75 μM, 16 h). **K** PI staining showing the cell death rates of control (sh-NC) and circTFRC knockdown (sh-circTFRC) AGS cells following treatment with RSL3 (1 μM) in the absence or presence of ferrostatin-1 (0.75 μM) for 16 h. **L** Flow cytometry showing the lipid ROS levels (stained with C11 BODIPY 581/591) in control (sh-NC) and circTFRC knockdown (sh-circTFRC) AGS cells following treatment with RSL3 (1 μM) in the absence or presence of ferrostatin-1 (0.75 μM) for 16 h. **M** Confocal microscopy showing the lipid ROS levels in control (sh-NC) and circTFRC knockdown (sh-circTFRC) AGS cells following treatment with RSL3 (1 μM) in the absence or presence of ferrostatin-1 (0.75 μM) for 16 h. Lipid ROS are stained with C11 BODIPY 581/591 probe (green). Nuclei are counterstained with DAPI (blue). Scale bar, 10 µm. Data are presented as mean ± SD. *P*-values were calculated using a two-tailed one-way ANOVA (**A**–**F**) or unpaired Student’s *t* test (**H**, **J**–**L**); **P < 0.01, ***P < 0.001, ****P < 0.0001. See also Fig. S4.

Ferroptosis resistance has been increasingly associated with GC tumorigenesis and progression [36, 37]. CircTFRC was proposed to contribute to GC advancement by modulating ferroptosis susceptibility. Supporting this notion, silencing circTFRC markedly elevated GC cell death (Figs. 4A, B and S4A, B), an effect abrogated by Fer-1, a ferroptosis inhibitor, but not by apoptosis (ZVAD-FMK) or necroptosis (necrostatin-1, Nec-1) inhibitors (Fig. S4C, D). CircTFRC depletion also enhanced lipid peroxidation, evidenced by increased lipid ROS accumulation (Figs. 4C, D and S4E), and elevated intracellular MDA (Fig. 4E, F), a terminal byproduct of lipid oxidation. Both lipid ROS and MDA elevations were attenuated by Fer-1 (Figs. 4C–F and S4E). Furthermore, the inhibitory effects of circTFRC knockdown on GC cell proliferation and motility were reversed upon Fer-1 treatment (Fig. 4G–J). These results collectively suggest that circTFRC acts as an intrinsic repressor of ferroptosis, thereby supporting GC cell growth and migratory capacity.

To further explore therapeutic implications, the combinatorial impact of circTFRC inhibition with the ferroptosis inducer RSL3 was assessed. CircTFRC silencing significantly amplified RSL3-induced cytotoxicity (Figs. 4K and S4F), accompanied by augmented lipid ROS (Figs. 4L and S4G) and MDA accumulation (Fig. S4H). Confocal microscopy revealed increased oxidized lipid deposition in the cytoplasm and plasma membranes of circTFRC-silenced cells (Fig. 4M). The enhanced cell death, oxidative lipid damage, and MDA levels triggered by the combined treatment were fully reversed by Fer-1 (Figs. 4K–M and S4F–H), confirming ferroptosis as the underlying mechanism. These findings indicate that circTFRC silencing substantially sensitizes GC cells to ferroptosis. Therapeutic strategies integrating circTFRC inhibition with ferroptosis-inducing agents may thus represent a promising direction in GC management.

### CircTFRC interacted with *SCD1* mRNA and enhanced its stability *via* recruiting ELAVL1

To investigate how circTFRC modulates ferroptosis in GC, the impact of circTFRC knockdown on GPX4 protein expression and enzymatic activity was examined, given GPX4’s established regulatory function in ferroptosis pathways [21]. While RSL3 treatment markedly suppressed both GPX4 protein expression and activity, consistent with previous reports [21, 23, 38] silencing circTFRC produced no observable effect on either parameter (Fig. S5A–B). Since iron (Fe^2+^) accumulation serves as the initial trigger for ferroptosis, subsequently leading to the production of ROS [23, 39], we then assessed intracellular Fe^2+^ levels using FerroOrange. The results showed that intracellular Fe^2+^ levels also remained unaltered following circTFRC knockdown (Fig. S5C). In contrast, a notable elevation in total intracellular ROS was detected (Fig. S5D–E), whereas mitochondrial ROS (mitoROS) levels showed minimal variation (Fig. S5F–G). These results suggest that circTFRC knockdown may induce ferroptosis in GC cells primarily via lipid peroxide accumulation at the plasma membrane, independent of GPX4 suppression or iron overload.

**Fig. 5:**
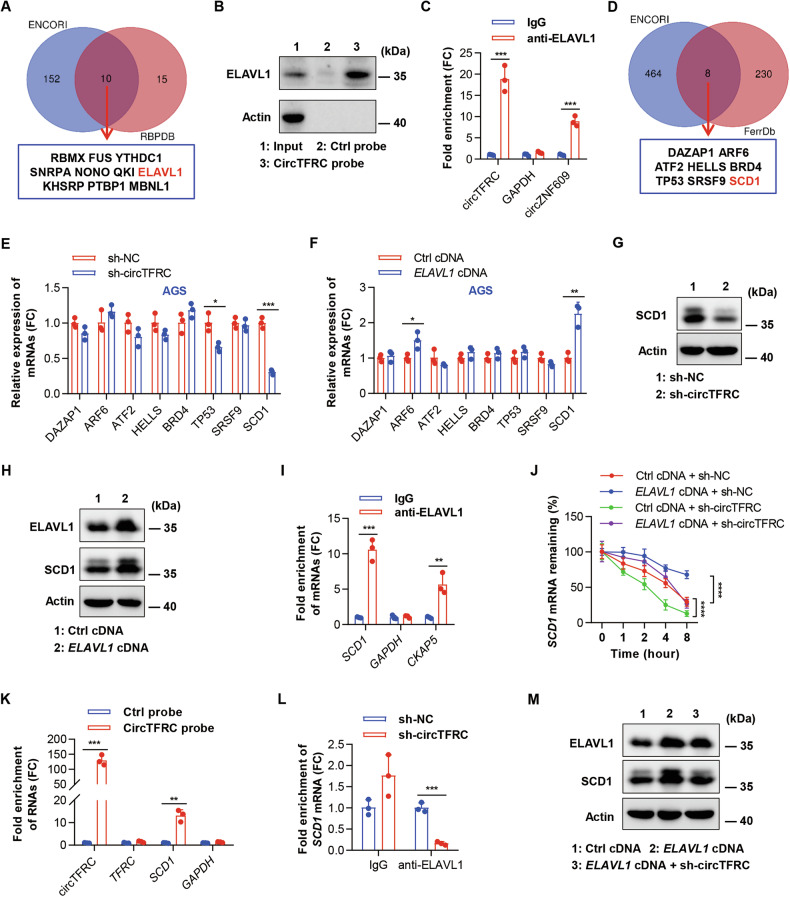
CircTFRC interacted with *SCD1* mRNA and enhanced its stability via recruiting ELAVL1. **A** Venn diagram showing the intersection of potential circTFRC-binding proteins predicted by ENCORI and RBPDB databases. **B** Representative Western blotting showing ELAVL1 enrichment upon circTFRC pulldown in GC cell lysates, with Actin as a negative control. **C** qRT–PCR analysis demonstrating circTFRC enrichment in an anti-ELAVL1 RIP assay in GC cells. IgG serves as a control. **D** Venn diagram displaying the intersection between ferroptosis-suppressor genes in the FerrDb database and ELAVL1-binding mRNAs predicted by ENCORI. **E** qRT–PCR analysis showing the expression of mRNAs in GC cells under control conditions (sh-NC) or circTFRC knockdown (sh-circTFRC). **F** qRT–PCR analysis showing the expression of mRNAs in GC cells transfected with control or *ELAVL1* overexpression vector. **G** Western blotting showing SCD1 protein levels in GC cells under control conditions (sh-NC) or circTFRC knockdown (sh-circTFRC), with Actin as a loading control. **H** Western blotting of ELAVL1 and SCD1 in GC cells transfected with control vector, or *ELAVL1* overexpression vector. **I** qRT–PCR analysis showing *SCD1* 3’ UTR enrichment in an anti-ELAVL1 RIP assay in GC cells, with *CKAP5* mRNA as a positive control and *GAPDH* mRNA as a negative control. **J** qRT–PCR analysis of *SCD1* mRNA stability in control, *ELAVL1*-overexpressing, circTFRC knockdown, and *ELAVL1-*overexpressing + circTFRC knockdown cells following Actinomycin D treatment (5 µg/mL) at various time points (0, 1, 2, 4, and 8 h). **K** qRT–PCR demonstrating circTFRC and *SCD1* 3’ UTR enrichment upon circTFRC pulldown in GC cell lysates. *GAPDH* serves as a negative control. **L** qRT–PCR showing *SCD1* 3’ UTR enrichment after anti-ELAVL1 RIP in GC cells under control conditions (sh-NC) or circTFRC knockdown (sh-circTFRC). **M** Representative Western blot of ELAVL1 and SCD1 in GC cells after the transfection of the control vector or *ELAVL1* overexpression vector or cotransfection of *ELAVL1* + sh-circTFRC vectors. The data are shown as the mean ± SD. The *P* values were determined by a two-tailed unpaired Student’s *t* test (**C**, **E**, **F**, **I**, **K**, **L**) or two-tailed two-way ANOVA (**J**); *P < 0.05, **P < 0.01, ***P < 0.001, ****P < 0.0001. See also Figs. S5 and S6.

Emerging evidence indicates that lipid metabolism plays a regulatory role in lipid peroxidation at the cell membrane, thereby influencing cellular susceptibility to ferroptosis [40–42]. Based on this, circTFRC was hypothesized to modulate membrane lipid peroxidation and ferroptotic cell death through regulation of lipid metabolism-related protein functions or gene expression. To test this premise, the investigation focused on proteins potentially interacting with circTFRC, a mechanism frequently implicated in circRNA-mediated modulation of protein activity and post-transcriptional regulation [43, 44]. Candidate circTFRC-binding proteins were screened using the ENCORI and RBPDB databases (Tables S4 and S5), yielding ten predicted interactors: ELAVL1, RBMX, FUS, YTHDC1, SNRPA, NONO, QKI, KHSRP, PTBP1, and MBNL1 (Fig. 5A). Among these, circTFRC was predicted to harbor 14 ELAVL1 binding sites, as identified via the CircInteractome database (Fig. S6A). Further computational analysis using RPISeq demonstrated high interaction probabilities between circTFRC and ELAVL1, with random forest (RF) and support vector machine (SVM) scores of 0.850 and 0.93, respectively (Fig. S6B). To experimentally validate the circTFRC–ELAVL1 interaction in GC cells, RNA pull-down assays employing a biotin-labeled circTFRC probe were performed. The probe specifically enriched endogenous ELAVL1, while β-actin remained undetected, confirming binding specificity (Fig. 5B). Additional verification through ELAVL1-mediated RIP assays showed selective enrichment of endogenous circTFRC and circZNF609 (a known ELAVL1-binding circRNA) [45], whereas *GAPDH* mRNA was not detected in the immunoprecipitates (Fig. 5C). Collectively, these results support the formation of circTFRC–ELAVL1 RNA-protein complexes in GC cells.

ELAVL1 (also known as HuR), a member of the ELAV-like RNA-binding protein family, binds to AU-rich elements (AREs) within the 3’ untranslated regions (3’UTR) of mRNAs, typically enhancing their stability and translational output [46]. Considering its established regulatory role, a potential interaction between circTFRC and ELAVL1 was hypothesized to influence the stability of specific mRNA transcripts in GC cells. Integration of ferroptosis suppressor genes from the FerrDb V2 database (Table S6) with predicted ELAVL1-associated mRNAs from the ENCORI database (Table S7) revealed eight overlapping candidates (*DAZAP1, ARF6, ATF2, HELLS, BRD4, TP53, SRSF9*, and *SCD1*) (Fig. 5D). Subsequent qRT–PCR analysis confirmed that *SCD1* mRNA expression was markedly reduced following circTFRC silencing (Fig. 5E) and elevated upon ELAVL1 overexpression in AGS cells (Fig. 5F), suggesting that *SCD1* serves as a convergent downstream target of both circTFRC and ELAVL1 in GC. Functionally, *SCD1* mediates the conversion of saturated fatty acids into monounsaturated fatty acids (MUFA), thereby enhancing cellular resilience to ferroptosis and mechanistically supporting the observed regulatory relationship [47–49]. WB analysis demonstrated that circTFRC knockdown led to a reduction in *SCD1* protein expression, whereas ELAVL1 overexpression enhanced its levels in GC cells (Fig. 5G, H). Analysis using AREsite2, a curated database of AU-/GU-/U-rich elements [50], identified multiple AREs—such as AUUUA and WUUUW motifs—within the 3′UTR of *SCD1* mRNA, suggesting potential regulatory interactions with ARE-binding proteins that may confer transcript stability. RPISeq prediction further indicated a high likelihood of interaction between ELAVL1 and the *SCD1* 3′UTR (Fig. S6C). This interaction was experimentally validated by RIP assays targeting ELAVL1 in GC cells, using *CKAP5* mRNA as a positive control—given its known association with ELAVL1 [45] and *GAPDH* mRNA as a negative control (Fig. 5I). Actinomycin D chase assays confirmed that ELAVL1 overexpression extended the half-life of *SCD1* mRNA (Fig. 5J). Consistently, *SCD1* expression was elevated in GC tissues (Fig. S6D), and its transcript levels positively correlated with *ELAVL1* mRNA abundance based on TCGA data (Fig. S6E). These results collectively delineate ELAVL1 as a key regulator of *SCD1* mRNA stability and translational output in GC cells.

The involvement of circTFRC in ELAVL1-mediated stabilization of *SCD1* mRNA was further characterized. Considering the potential for circRNAs to interact with target mRNAs via high-complementarity base pairing [45, 51], a BLAST alignment was performed between circTFRC and the *SCD1* 3’UTR sequences. This analysis revealed a 12-nucleotide region of perfect complementarity within the *SCD1* 3’UTR aligning with circTFRC (Fig. S6F). RNA pull-down assays using biotinylated circTFRC probes demonstrated significant enrichment of both circTFRC and *SCD1* 3’UTR, whereas *TFRC* and *GAPDH* mRNAs were not enriched, confirming target specificity (Fig. 5K). To assess the influence of circTFRC on ELAVL1–*SCD1* 3’UTR association, RIP assays for ELAVL1 were conducted in both control and circTFRC-silenced GC cells. Disruption of circTFRC expression nearly eliminated ELAVL1 binding to the *SCD1* 3’UTR (Fig. 5L), indicating that this interaction is contingent on circTFRC. Furthermore, circTFRC silencing markedly reduced *SCD1* mRNA stability (Fig. 5J). The stabilizing effect and increased protein synthesis driven by ELAVL1 overexpression were nullified upon circTFRC depletion (Fig. 5J, M), while ELAVL1 protein levels remained unchanged (Fig. 5M). These results indicate that circTFRC acts as a molecular scaffold that anchors ELAVL1 to *SCD1* mRNA, thereby enhancing mRNA stability and promoting *SCD1* expression in GC cells.

### CircTFRC exerted an oncogenic effect *via* SCD1 in GC cells

**Fig. 6:**
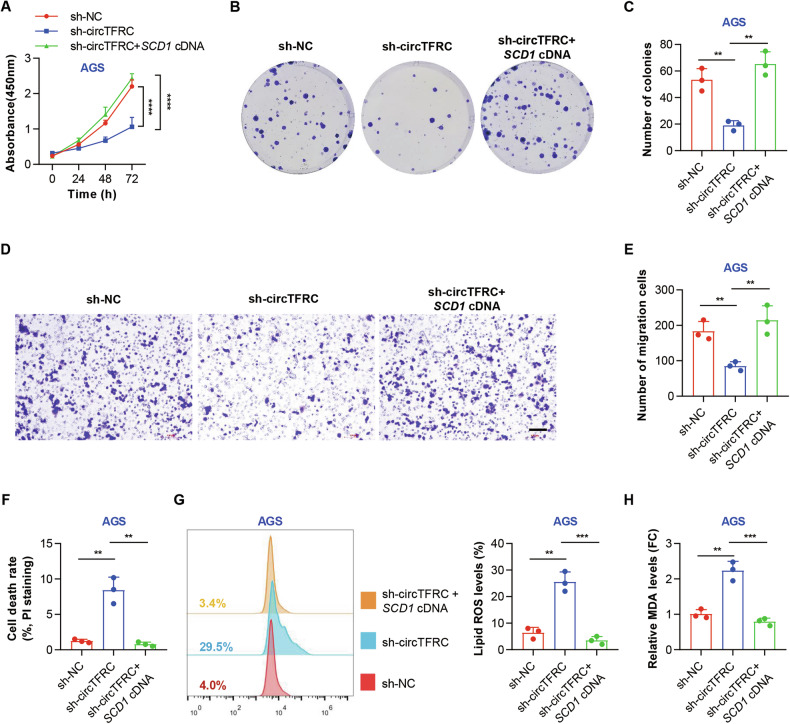
CircTFRC exerted an oncogenic effect via SCD1 in GC cells. **A** CCK-8 assay showing the proliferation of AGS cells under control conditions (sh-NC), or upon circTFRC knockdown (sh*-*circTFRC) or sh*-*circTFRC + *SCD1* vector cotransfection at indicated time points (0, 24, 48, and 72 h). **B**, **C** Plate colony formation assay assessing colony formation in AGS cells under control conditions (sh-NC), or upon circTFRC knockdown (sh*-*circTFRC) or sh*-*circTFRC + *SCD1* vector cotransfection over 10 days. **D, E** Transwell assay showing the migration of AGS cells under control conditions (sh-NC), or upon circTFRC knockdown (sh*-*circTFRC) or sh*-*circTFRC + *SCD1* vector cotransfection over 24 h. Scale bar, 100 µm. **F** PI staining showing the cell death rates of AGS cells under control conditions (sh-NC), or upon circTFRC knockdown (sh*-*circTFRC) or sh*-*circTFRC + *SCD1* vector cotransfection. **G** Flow cytometry showing the lipid ROS levels (stained with C11 BODIPY 581/591) of AGS cells under control conditions (sh-NC), or upon circTFRC knockdown (sh*-*circTFRC) or sh*-*circTFRC + *SCD1* vector cotransfection. ROS reactive oxygen species. **H** ELISA assays showing the relative MDA levels of AGS cells under control conditions (sh-NC), or upon circTFRC knockdown (sh*-*circTFRC) or sh*-*circTFRC + *SCD1* vector cotransfection. MAD malondialdehyde. The data are shown as the mean ± SD. The *P* values were determined by a two-tailed unpaired Student’s *t* test (**C**, **E**–**H**) or two-way ANOVA (**A**); **P < 0.01, ***P < 0.001, ****P < 0.0001. See also Fig. S7.

*SCD1* has been reported to be upregulated during GC development, contributing to tumor proliferation and metastasis by modulating oncogenic fatty acid metabolism and protecting GC cells from ferroptosis [52, 53]. To clarify its involvement in circTFRC-induced tumorigenic effects, in vitro rescue assays were performed. As shown in Fig. S7A, overexpression of *SCD1* restored its expression following circTFRC knockdown. Functional assays, including CCK-8 (Fig. 6A), colony formation (Fig. 6B, C), and Transwell migration (Fig. 6D, E), consistently indicated that *SCD1* overexpression reversed the inhibitory effects of circTFRC silencing on GC cell proliferation and motility. Additionally, *SCD1* overexpression attenuated the increase in cell death triggered by circTFRC depletion (Figs. 6F and S7B) and reduced the accumulation of lipid ROS and MDA induced by circTFRC suppression (Fig. 6G, H). These results suggest that circTFRC promotes GC progression and suppresses ferroptosis through *SCD1* upregulation.

### CircTFRC knockdown promoted ferroptosis and inhibited GC progression in vivo

**Fig. 7:**
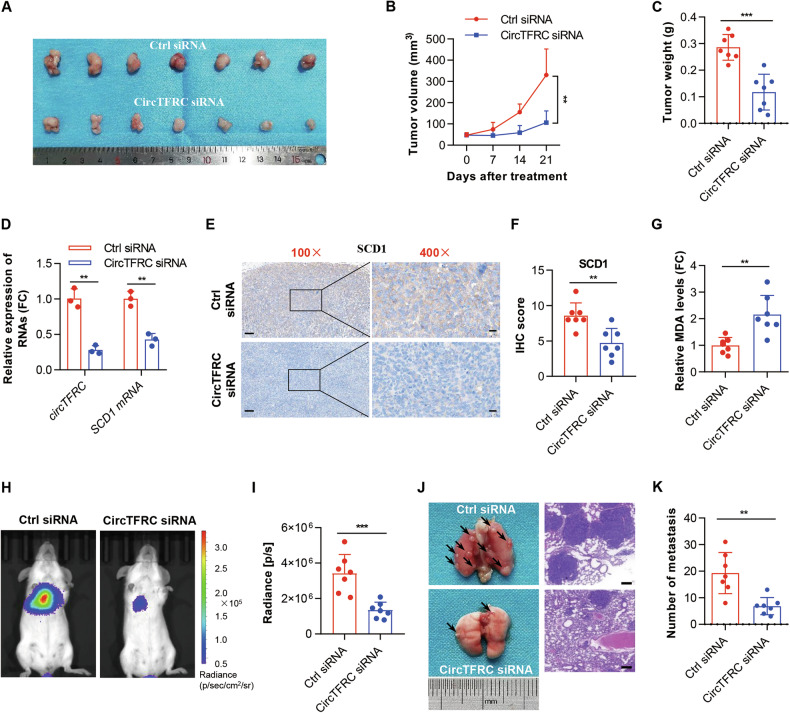
CircTFRC knockdown promoted ferroptosis and inhibited GC progression in vivo. **A** Images of tumors in mice received intratumoral injections of control siRNA or in vivo-optimized circTFRC siRNA (*n* = 7 per group). **B** Tumor growth curves in mice in each group. **C** Tumor weights in mice in each group. **D** qRT‒PCR showing the expression levels of circTFRC and *SCD1* mRNA in control tumors (Ctrl siRNA) or circTFRC knockdown tumors (CircTFRC siRNA). **E**, **F** Immunohistochemistry (IHC) staining showing the expression levels of SCD1 protein in control tumors (Ctrl siRNA) or circTFRC knockdown tumors (CircTFRC siRNA). Scale bar, 100 µm (left), 20 µm (right). **G** ELISA assays showing the relative intratumoral levels of MDA in control tumors (Ctrl siRNA) or circTFRC knockdown tumors (CircTFRC siRNA). MAD malondialdehyde. **H**, **I** The spread of GC cells in control group (Ctrl siRNA) or circTFRC knockdown group (CircTFRC siRNA) was presented through bioluminescence imaging (BLI) using the IVIS® Spectrum imaging system (*n* = 7 per group). Bioluminescent signals were detected by administering 150 mg/kg of the luciferase substrate D-luciferin via intraperitoneal injection 10 min before imaging. **J** Images of lung metastasis nodules in mice received intravenous injections of control siRNA or in vivo-optimized circTFRC siRNA. Scale bar, 200 µm. **K** The number of lung metastasis nodules in mice in control group (Ctrl siRNA) or circTFRC knockdown group (CircTFRC siRNA). The data are shown as the mean ± SD. The *P* values were determined by a two-tailed unpaired Student’s *t* test (**C**, **D**, **F**, **G**, **I**, **K**) or two-way ANOVA (**B**); **P < 0.01, ***P < 0.001.

To investigate the in vivo function of circTFRC in GC ferroptosis and tumor progression, subcutaneous xenograft and systemic metastasis mouse models were established and treated with siRNAs specifically optimized for in vivo silencing of circTFRC. Tumors derived from circTFRC siRNA-treated xenografts displayed significantly reduced volume and weight compared to those receiving control siRNAs (Fig. 7A–C), indicating substantial suppression of GC cell proliferation upon circTFRC depletion. qRT–PCR analysis revealed marked downregulation of circTFRC and *SCD1* mRNA in tumors from the circTFRC-targeted group (Fig. 7D), corroborated by immunohistochemical (IHC) staining, which demonstrated decreased *SCD1* protein expression following circTFRC silencing (Fig. 7E, F). Elevated intratumoral MDA levels were detected in circTFRC-depleted tumors, implicating enhanced ferroptotic cell death in vivo (Fig. 7G). In systemic metastasis models, intravenous delivery of circTFRC siRNAs led to a notable reduction in pulmonary metastatic nodules relative to controls (Fig. 7H–K), reflecting suppressed metastatic potential. Collectively, the data support that circTFRC knockdown inhibits GC tumor growth and dissemination in vivo, potentially by promoting ferroptosis through *SCD1* downregulation.

## Discussion

Recent evidence implicates circRNAs in the regulation of ferroptosis, with downstream effects on tumor growth, metastasis, and chemoresistance [14, 15, 54]. Despite these associations, the mechanistic basis of circRNA-mediated ferroptosis modulation remains poorly understood, and no circRNA-targeted therapies have received clinical approval to date [55]. In this study, hsa_circ_0068608 (circTFRC), a previously unrecognized ferroptosis-related circRNA, was identified as a key mediator of ferroptosis resistance in GC cells, concurrently promoting their proliferation and metastatic potential. Mechanistically, circTFRC enhanced the interaction between ELAVL1 and *SCD1* mRNA, leading to increased *SCD1* stability and expression, which in turn contributed to ferroptosis evasion and tumor advancement. In vivo data further support circTFRC as a viable candidate for ferroptosis-oriented therapeutic intervention in GC.

The *TFRC* gene encodes a membrane-bound receptor essential for iron uptake through receptor-mediated endocytosis [56, 57]. Although *TFRC* mRNA and its splice variants have been extensively examined in the contexts of cancer biology and ferroptosis regulation [58, 59], circRNAs originating from *TFRC* remain insufficiently characterized. Among them, hsa_circ_0068631—derived from exons 2–3—has been implicated in tumorigenesis across multiple malignancies, including breast cancer [34], ovarian cancer [35], and cutaneous squamous cell carcinoma [60]. In contrast, this study identified a distinct circRNA isoform, hsa_circ_0068608 (circTFRC), spanning exons 2–18, which exhibited elevated expression in GC and independently influenced ferroptosis and tumor development, without reliance on either *TFRC* mRNA or hsa_circ_0068631. These findings establish circTFRC as a previously unrecognized regulatory element in GC pathogenesis.

Although circRNAs are recognized for their roles in miRNA sequestration, protein binding, and translation of functional peptides, their direct involvement in regulating mRNA homeostasis remains unclear [12, 61]. An emerging aspect of circRNA function involves the assembly of ribonucleoprotein (RNP) complexes through their scaffold-like capacity to coordinate RBPs and mRNA substrates [45, 51]. In this context, circTFRC was shown to directly associate with *SCD1* mRNA. This interaction enhanced *SCD1* mRNA stability and translation efficiency via ELAVL1 recruitment. ELAVL1, a multifaceted RNA-binding protein involved in various post-transcriptional regulatory processes, engages numerous competing RNA targets within the cell [62, 63]. Possessing 14 putative ELAVL1-binding motifs, circTFRC appears to act as a molecular carrier that augments ELAVL1 proximity to specific transcripts, including *SCD1* mRNA, which exhibits relatively low intrinsic affinity for ELAVL1 within its 3’UTR. This scaffolding mechanism may be essential for shifting ELAVL1 binding preference toward *SCD1*, thereby stabilizing its transcript. Functionally, *SCD1*, a rate-limiting enzyme in the desaturation of saturated to monounsaturated fatty acids, governs lipid metabolic flux and contributes to membrane lipid homeostasis [64, 65]. Elevated *SCD1* expression is frequently observed in malignancies, where it supports tumor growth and ferroptosis resistance through lipid composition reprogramming [66, 67]. The present data indicate that circTFRC-mediated *SCD1* induction suppresses ferroptosis and accelerates GC progression and dissemination, illustrating the substantial regulatory impact of the circTFRC/*SCD1* mRNA axis on ferroptosis vulnerability and metastatic behavior.

This study identified a significant antitumor response in preclinical models following circTFRC silencing using in vivo-optimized siRNAs. The therapeutic potential of circTFRC arises from its capacity to modulate cancer cell susceptibility to ferroptosis. Given its mechanism of action involves direct base-pairing with *SCD1* mRNA, targeting circTFRC with siRNAs to disrupt this RNA-RNA interaction may offer a complementary strategy for suppressing tumor progression and dissemination. With RNA-based therapies gaining momentum, as reflected by the expanding pipeline of siRNA and antisense oligonucleotide candidates in clinical trials [68, 69], RNA-RNA interactions present a compelling framework for the identification of novel therapeutic targets [70, 71].

## Conclusions

This study first establishes that the interaction between circTFRC and *SCD1* mRNA plays a central role in protecting GC cells from ferroptosis and promoting tumor progression. Interruption of this interaction may enhance the therapeutic efficacy of ferroptosis-based interventions in future GC treatment strategies.

## Supplementary information

Supplementary Materials

Original data (full uncropped western blots)

Original data (qPCR data)

## Data Availability

All data supporting the findings of this study are available within the article, its Supplementary Information files, and from the corresponding author(s) upon reasonable request.
