# Supplementary material for: Circular RNA TFRC/SCD1 mRNA interaction regulates ferroptosis and metastasis in gastric cancer
Source: Cell Death Dis. 2025 Jun 5;16(1):436. doi: 10.1038/s41419-025-07759-x (PMC12141735; doi:10.1038/s41419-025-07759-x)
Supplement: Supplementary file 1 — Supplementary Materials [file 41419_2025_7759_MOESM1_ESM.pdf]

Supplementary Figures and Figure legends

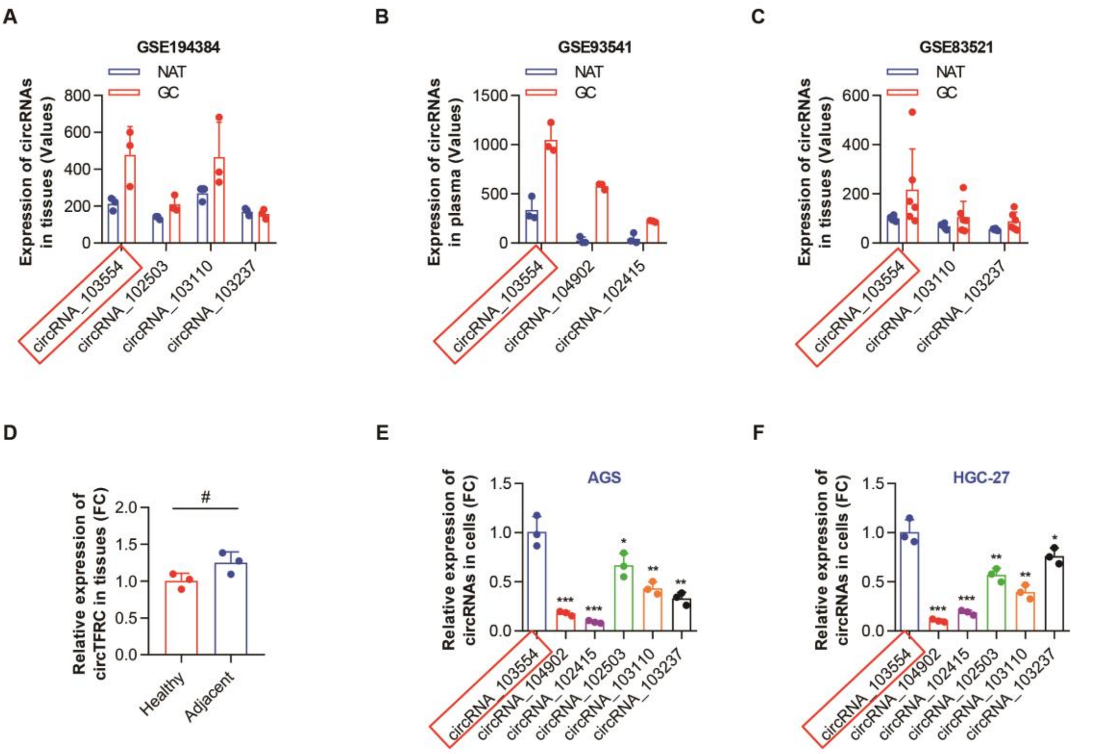

Figure S1. Comparison of the expression abundance of circTFRC and other circRNAs in GC. **(A, B, C)** The analysis of data from three microarray dataset (GSE194384, GSE93541 and GSE83521) showing the expression abundance of circRNA_103554 (circTFRC) and other known upregulated circRNAs in GC tissues or patient plasma. **(D)** qRT–PCR showing the expression levels of circRNA_103554 (circTFRC) in three normal-appearing tissues adjacent to GC and three matched normal gastric mucosal samples from healthy donors. **(E, F)** qRT–PCR showing the expression levels of circRNA_103554 (circTFRC) and other known upregulated circRNAs in AGS and HGC-27 cells. The data are shown as the mean ± SD. The *P* values were determined by a two-tailed paired Student's t test (A, C) or unpaired Student's t test (B, D–F); **P*<0.05, ***P*<0.01, ****P*<0.001. Related to **Figure 1**.

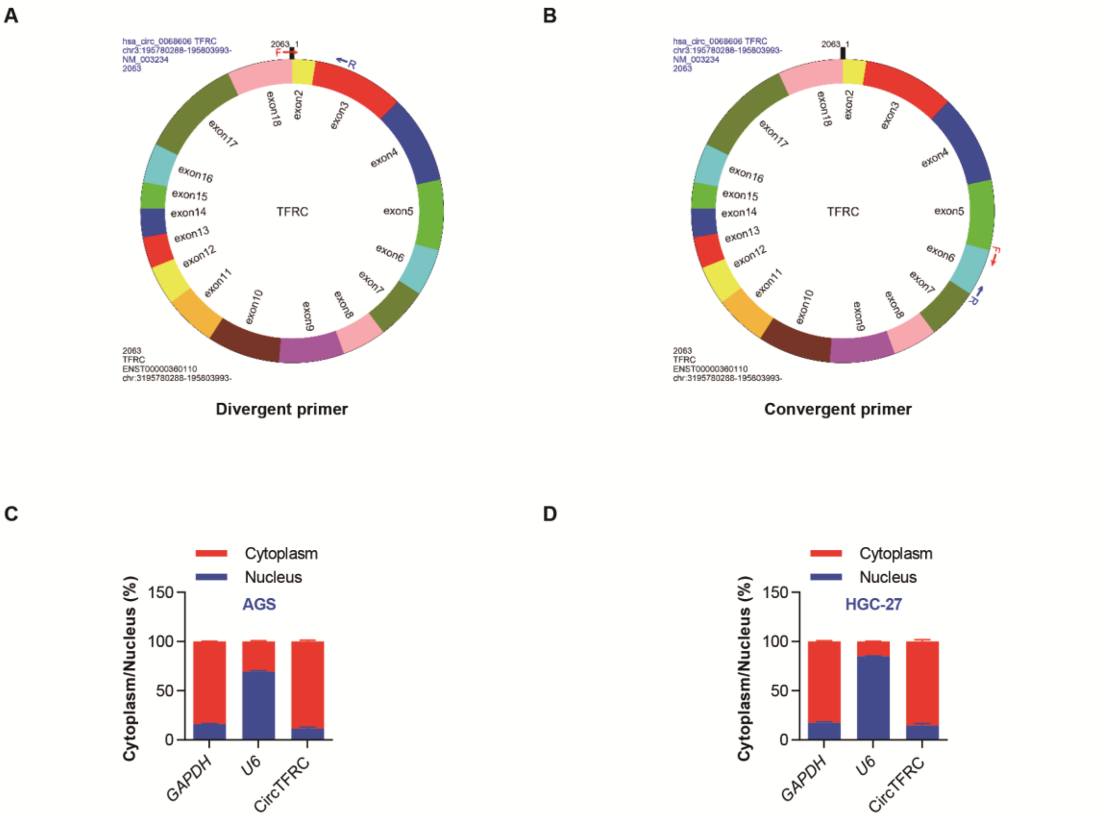

Figure S2. Primer design and subcellular localization of circTFRC. **(A, B)** Schematic representation of the design of divergent primers (DP) and convergent primers (CP) for circTFRC using circPrimer software 2.0. **(C, D)** Cytoplasmic and nuclear mRNA fractionation experiments showing the location of circTFRC in AGS and HGC-27 cells. *U6* and *GAPDH* were used as positive controls in the nucleus and cytoplasm, respectively. The data are shown as the mean ± SD. Related to **Figure 2**.

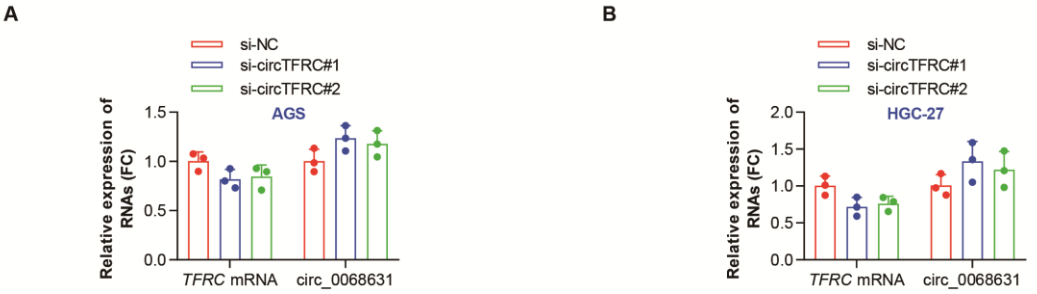

Figure S3. The expression of RNAs in GC cells with circTFRC knockdown. (A) qRT–PCR showing the expression levels of *TFRC* mRNA and circ_0068631 in AGS cells under control conditions (si-NC) or upon circTFRC knockdown (si-circTFRC). **(B)** qRT–PCR showing the expression levels of *TFRC* mRNA and circ_0068631 in HGC-27 cells under control conditions (si-NC) or upon circTFRC knockdown (si-circTFRC). The data are shown as the mean ± SD. The *P* values were determined by a two-tailed one-way ANOVA (A, B). Related to **Figure 3**.

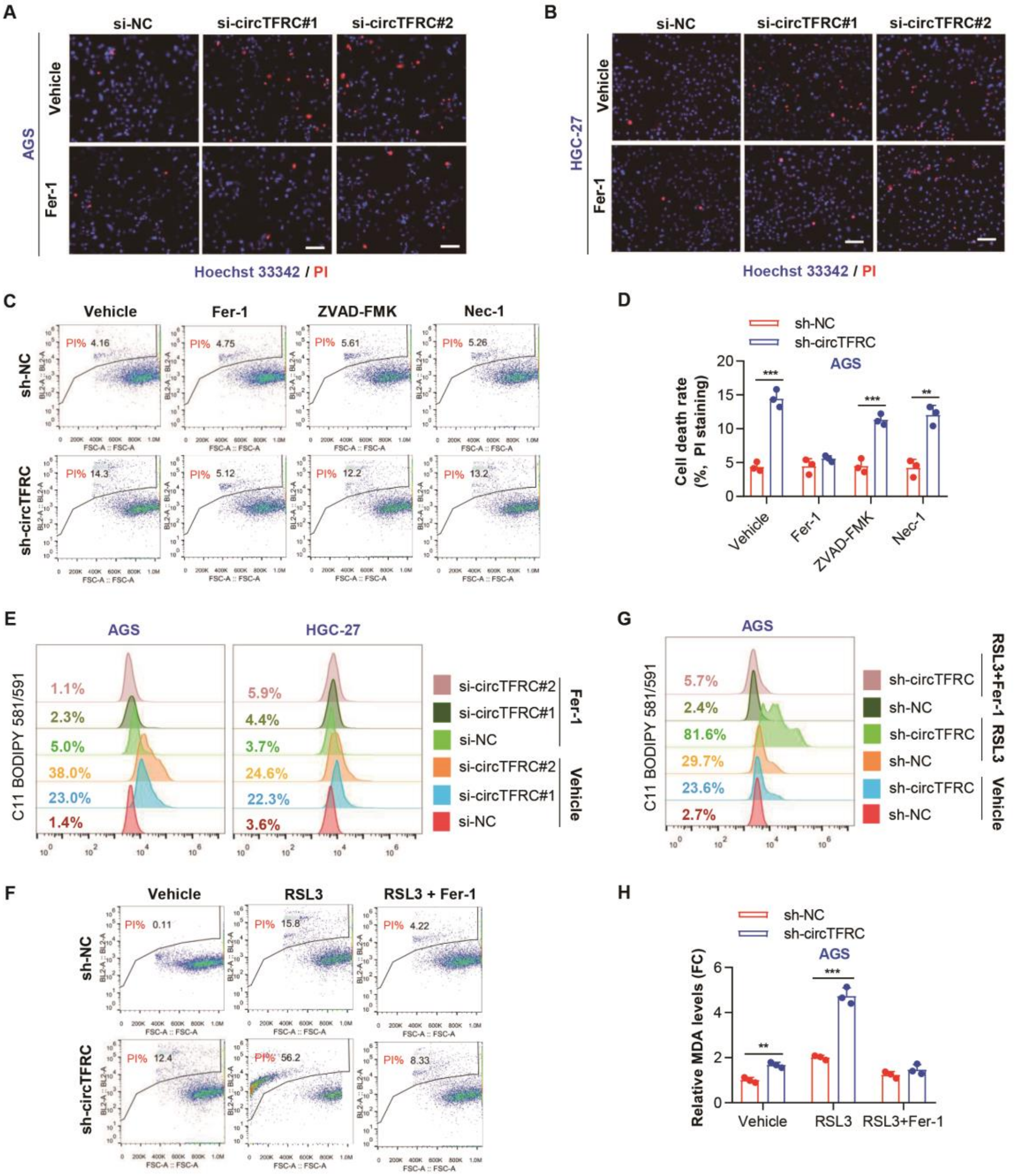

Figure S4. CircTFRC knockdown promoted the ferroptosis of GC cells. (A, B) Propidium iodide (PI) staining (fluorescence image) showing the cell death rates of control (si-NC) and circTFRC knockdown (si-circTFRC) AGS and HGC-27 cells in the absence or presence of ferrostatin-1 (0.75 μM, 16 hours). Scale bar, 100 μm. **(C, D)** PI staining (flow cytometry) showing the cell death rates of control (sh-NC) and circTFRC knockdown (sh-circTFRC) AGS cells in the absence or presence of ferrostatin-1 (0.75 μM, 16 hours), or ZVAD-FMK (10 μM, 16 hours) or Nec-1(10 μM, 16 hours). Nec-1: necrostatin-1. **(E)** Flow cytometry showing the lipid ROS levels (stained with C11 BODIPY 581/591) in control (si-NC) and circTFRC knockdown (si-circTFRC) AGS and HGC-27 cells in the absence or presence of ferrostatin-1 (0.75 μM, 16 hours). ROS: reactive oxygen species. **(F)** PI staining (flow cytometry) showing the cell death rates of control (sh-NC) and circTFRC knockdown (sh-circTFRC) AGS cells following treatment with RSL3 (1 μM) in the absence or presence of ferrostatin-1 (0.75 μM) for 16 hours. **(G)** Flow cytometry showing the lipid

ROS levels (stained with C11 BODIPY 581/591) in control (sh-NC) and circTFRC knockdown (sh-circTFRC) AGS cells following treatment with RSL3 (1 μM) in the absence or presence of ferrostatin-1 (0.75 μM) for 16 hours. **(H)** ELISA assays showing the relative MDA levels in control (sh-NC) and circTFRC knockdown (sh-circTFRC) AGS cells following treatment with RSL3 (1 μM) in the absence or presence of ferrostatin-1 (0.75 μM) for 16 hours. MDA: malondialdehyde. Data are presented as mean ± SD. *P*-values were calculated using a two-tailed unpaired Student's t-test (D, H); ***P* < 0.01, ****P* < 0.001. Related to **Figure 4**.

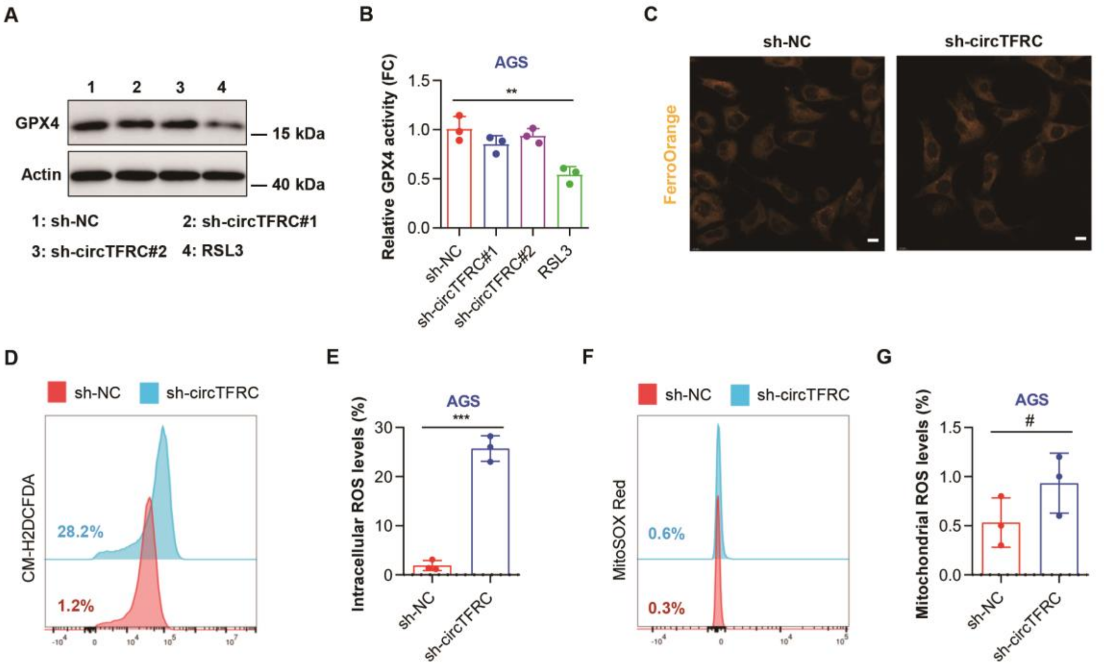

Figure S5. CircTFRC regulated ferroptosis through a unique mechanism in GC cells. (A) Western blotting showing GPX4 protein levels in AGS cells under control conditions (sh-NC) or circTFRC knockdown (sh-circTFRC) or following treatment with RSL3 (1 μM, 16 hours), with Actin as a loading control. **(B)** Relative glutathione peroxidase activity were quantified in AGS cells under control conditions (sh-NC) or circTFRC knockdown (sh-circTFRC) or following treatment with RSL3 (1 μM, 16 hours). **(C)** FerroOrange staining showing the intracellular Fe^{2+} levels in AGS cells under control conditions (sh-NC) or circTFRC knockdown (sh-circTFRC). Scale bar, 10 μm. **(D, E)** Flow cytometry showing the intracellular ROS levels (stained with CM-H2DCFDA) in AGS cells under control conditions (sh-NC) or circTFRC knockdown (sh-circTFRC). **(F, G)** Flow cytometry showing the mitochondrial ROS levels (stained with MitoSOX Red) in AGS cells under control conditions (sh-NC) or circTFRC knockdown (sh-circTFRC). Data are presented as mean ± SD. *P*-values were calculated using a two-tailed unpaired Student's t-test (B, E, G); ***P* < 0.01, ****P* < 0.001. Related to **Figure 5**.

A

Circular RNA	Tag Name	% Identity	Alignment Length	Mismatches	Gap Openings	Tag Start	Tag End	Circular RNA Start	Circular RNA ENd
hsa_circ_0068606	Kishore_MNase_ELAVL1_Par_Clip_chr3_Minus_195791221_195791261_41	100.00	41	0	0	1	41	1260	1300
hsa_circ_0068606	Kishore_MNase_ELAVL1_Par_Clip_chr3_Minus_195789735_195789777_43	100.00	43	0	0	1	43	1375	1417
hsa_circ_0068606	Kishore_MNase_ELAVL1_Par_Clip_chr3_Minus_195787067_195787107_41	100.00	41	0	0	1	41	1503	1543
hsa_circ_0068606	Kishore_MNase_ELAVL1_Par_Clip_chr3_Minus_195785448_195785488_41	100.00	41	0	0	1	41	1575	1615
hsa_circ_0068606	Kishore_MNase_ELAVL1_Par_Clip_chr3_Minus_195785195_195785237_43	100.00	42	0	0	2	43	1619	1660
hsa_circ_0068606	Kishore_MNase_ELAVL1_Par_Clip_chr3_Minus_195782107_195782147_41	100.00	41	0	0	1	41	1726	1766
hsa_circ_0068606	Kishore_MNase_ELAVL1_Par_Clip_chr3_Minus_195782062_195782103_42	100.00	42	0	0	1	42	1770	1811
hsa_circ_0068606	Kishore_MNase_ELAVL1_Par_Clip_chr3_Minus_195781956_195782006_51	100.00	51	0	0	1	51	1867	1917
hsa_circ_0068606	Kishore_MNase_ELAVL1_Par_Clip_chr3_Minus_195780351_195780391_41	100.00	41	0	0	1	41	1961	2001
hsa_circ_0068606	Kishore_MNase_ELAVL1_Par_Clip_chr3_Minus_195802047_195802087_41	100.00	41	0	0	1	41	204	244
hsa_circ_0068606	Kishore_MNase_ELAVL1_Par_Clip_chr3_Minus_195800861_195800957_97	100.00	97	0	0	1	97	301	397
hsa_circ_0068606	Kishore_MNase_ELAVL1_Par_Clip_chr3_Minus_195800813_195800853_41	100.00	41	0	0	1	41	405	445
hsa_circ_0068606	Kishore_MNase_ELAVL1_Par_Clip_chr3_Minus_195798298_195798340_43	100.00	43	0	0	1	43	637	679
hsa_circ_0068606	Kishore_MNase_ELAVL1_Par_Clip_chr3_Minus_195802181_195802221_41	100.00	41	0	0	1	41	70	110

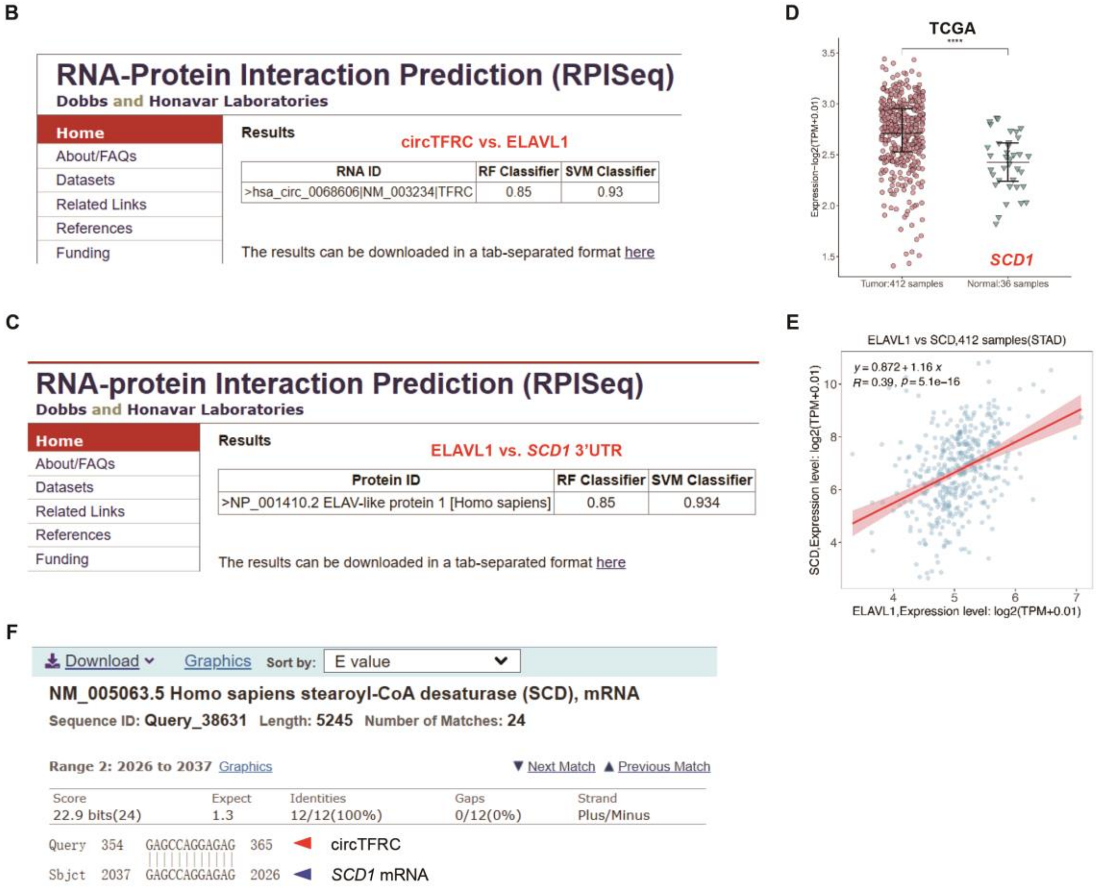

Figure S6. Prediction of the interaction between circTFRC and ELAVL1 or *SCD1* mRNA. (A) The binding sites between circTFRC and ELAVL1 predicted by the CircInteractome database. **(B)** The interaction between circTFRC and ELAVL1 predicted by the RNA–Protein Interaction Prediction (RPISeq) analysis. **(C)** The interaction between ELAVL1 and *SCD1* 3'UTR predicted by the RNA–Protein Interaction Prediction (RPISeq) analysis. **(D)** The differential expression of *SCD1* mRNA between GC tissues and normal tissues based on the analysis of TCGA database. **(E)** The correlation of *SCD1* mRNA expression and *ELAVL* mRNA expression in GC tissues based on the analysis of TCGA database. **(F)** Identification of highly reverse complementary regions in the sequences of circTFRC and *SCD1* 3'UTR using the Basic Local Alignment Search Tool (BLAST). The data are shown as the mean ± SD. The *P* values were determined by a two-tailed unpaired Student's t test (D); ****P<0.0001. Related to **Figure 5**.

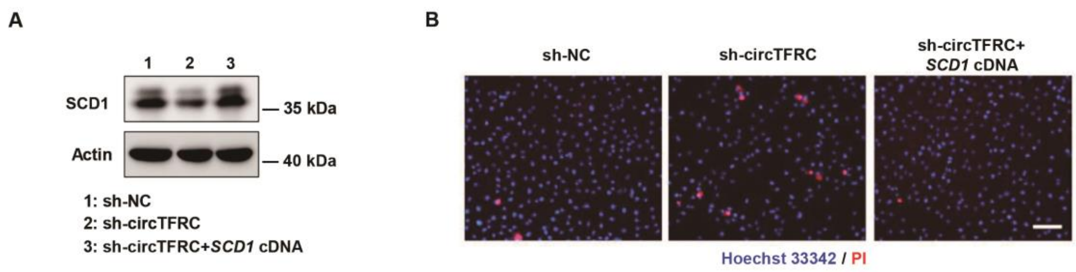

Figure S7. CircTFRC exerted oncogenic effect via SCD1 in GC cells. (A) Representative Western blot of SCD1 protein in GC cells under control conditions (sh-NC) or upon circTFRC knockdown (sh-circTFRC) or sh-circTFRC + *SCD1* vector cotransfection. **(B)** Propidium iodide (PI) staining showing the cell death rates of AGS cells under control conditions (sh-NC), or upon circTFRC knockdown (sh-circTFRC) or sh-circTFRC + *SCD1* vector cotransfection. Scale bar, 100 μm. Related to **Figure 6**.

Supplementary Tables

Table S1 Primers used in this article

Gene names	Forward primers (5'→3')	Reverse primers (5'→3')
circRNA_103554 (circTFRC) divergent primer	TGATCGTGTCATGAGAGTTCTTC	TATCGCCATCTACTTGCCGA
circRNA_103554 (circTFRC) convergent primer	CGCTCAAAACTCGGTGATCA	TGCAGCCTTACTATACGCCA
TFRC	GGCTACTTGGGCTATTGTAAAGG	CAGTTTCTCCGACAACTTTCTCT
SCD1	AAACCTGGCTTGCTGATG	GGGGGCTAATGTTCTTGTCA
ELAVL1	GTCCTCGTGGATCAGACTAC	TCATGTGATCGACGCCCATG
CKAP5	GGACACAAAGGACATTTCTGCAC	TTGGTTCCAGTATTCCCTGCG
GAPDH	GGTCGGAGTCAACGGATTTG	ATGAGCCCCAGCCTTCTCCAT
U6	CGCTTCGGCAGCACATATAC	TTCACGAATTTGCGTGTCATC
circRNA_102503 (circURI1)	GTCCATACTAATGAAGTCAC	CAAGGTGCTGAGTCTTTCTC
circRNA_103110 (circNRIP1)	TTCTCAGAAAGCAGAGGCTCAG	GGCTGTGTTTCTCCCAAATGTT
circRNA_103237 (circ-RanGAP1)	AGATTCTGGACCCTAACACTGG	CTCTTGCCTTTGAAACTCAGCT
circRNA_104902 (circ_0088300)	CCCAGATTGGAATCTATCTGTAGCA	ATTACACTTTTCAAATCATCCAGCA
circRNA_102415 (circ_0007376)	ATCGACTCCATGGCCAACTC	AAGCCCCGGAGAACAGC
circ_0068631	GCTGACAATAACACAAAGGCC	CCACACAGAAGAACCAATCAAGA
circZNF609	AAACCGGAGCCAGAGGAAGG	CAGCTATGTTCTCAGACCTGC

Table S2. The differentially expressed circRNAs in GSE194384 microarray dataset

circRNAs ID	logFC	AveExpr	t	P.Value	adj.P.Val	B
hsa_circRNA_005921	2.360187	8.263788	2.497057	0.035253	0.187671	-3.83026
hsa_circRNA_101017	2.248508	9.020831	11.70385	1.45E-06	0.013689	5.316379
hsa_circRNA_102064	1.98163	7.540658	2.970554	0.016543	0.153485	-3.10192
hsa_circRNA_033388	1.931448	9.27921	8.2562	2.31E-05	0.021944	3.111752
hsa_circRNA_009618	1.919589	10.14714	9.749741	6.29E-06	0.020838	4.195324
hsa_circRNA_033392	1.900201	9.350485	7.272086	6.06E-05	0.03165	2.262608
hsa_circRNA_102368	1.783266	10.39054	9.221264	9.77E-06	0.021781	3.83739
hsa_circRNA_403817	1.77023	7.70054	3.433467	0.00803	0.130929	-2.3974
hsa_circRNA_102738	1.766555	7.001345	2.977347	0.016366	0.153442	-3.09148
hsa_circRNA_405566	1.724012	7.765667	4.379343	0.001993	0.086965	-1.03442
hsa_circRNA_027934	1.691434	8.486473	4.080456	0.003054	0.101084	-1.45127
hsa_circRNA_000799	1.685846	11.80134	6.26873	0.00018	0.043806	1.265224
hsa_circRNA_105039	1.682999	8.463672	4.811822	0.001099	0.071404	-0.45654
hsa_circRNA_000479	1.669693	8.720431	3.701894	0.00534	0.117667	-1.99825
hsa_circRNA_407173	1.666274	9.854319	8.207949	2.42E-05	0.021944	3.072823
hsa_circRNA_052621	1.656809	9.310065	7.546734	4.59E-05	0.03165	2.511659
hsa_circRNA_000372	1.636436	8.679728	10.69147	3.02E-06	0.013689	4.772348
hsa_circRNA_001168	1.626884	7.477858	5.372627	0.000529	0.057445	0.246896
hsa_circRNA_001589	1.62549	10.97573	6.077188	0.000225	0.047839	1.058134
hsa_circRNA_004662	1.622287	9.810397	7.367182	5.50E-05	0.03165	2.349947
hsa_circRNA_058192	1.620374	9.432821	5.657701	0.000371	0.054491	0.584687
hsa_circRNA_002701	1.586144	10.13484	6.944867	8.54E-05	0.03285	1.952866
hsa_circRNA_104323	1.580444	8.026245	6.928887	8.68E-05	0.03285	1.937367
hsa_circRNA_034537	1.574696	7.931206	7.178721	6.68E-05	0.03165	2.175699
hsa_circRNA_000210	1.567621	9.093801	3.840394	0.004343	0.108464	-1.79582
hsa_circRNA_036567	1.535099	10.40361	9.512189	7.65E-06	0.020838	4.037659
hsa_circRNA_023610	1.52212	10.18122	5.19883	0.000661	0.060962	0.034449
hsa_circRNA_043398	1.501192	6.25575	2.918009	0.01798	0.155499	-3.18271
hsa_circRNA_055879	1.490965	9.588041	11.14888	2.15E-06	0.013689	5.027232
hsa_circRNA_102710	1.489755	8.049868	7.5029	4.79E-05	0.03165	2.472562
hsa_circRNA_015298	1.47953	6.396115	3.99973	0.003434	0.103366	-1.5662
hsa_circRNA_105038	1.473935	7.977283	4.182311	0.002636	0.094296	-1.30766
hsa_circRNA_002447	1.470802	7.301999	7.390731	5.37E-05	0.03165	2.371392
hsa_circRNA_081069	1.470186	8.459447	8.4508	1.93E-05	0.021781	3.266088
hsa_circRNA_058819	1.463256	7.380333	6.851139	9.44E-05	0.034738	1.861449
hsa_circRNA_000486	1.447049	9.531135	8.445808	1.94E-05	0.021781	3.262182
hsa_circRNA_079614	1.437732	8.116373	2.66779	0.026804	0.173369	-3.56804
hsa_circRNA_405852	1.436828	7.837076	4.289417	0.002263	0.087978	-1.15837
hsa_circRNA_100785	1.430541	8.764476	7.711944	3.89E-05	0.03118	2.656848
hsa_circRNA_101340	1.428063	7.666314	8.676678	1.57E-05	0.021781	3.440009
hsa_circRNA_100278	1.427944	10.5802	7.3053	5.86E-05	0.03165	2.293247

hsa_circRNA_102039	1.42499	11.02546	3.719551	0.005201	0.116014	-1.9723
hsa_circRNA_102103	1.424432	8.915593	3.166106	0.012158	0.143545	-2.80238
hsa_circRNA_404096	1.421492	8.225714	8.369849	2.08E-05	0.021781	3.2024
hsa_circRNA_104288	1.419385	8.467569	5.285742	0.000591	0.059114	0.14131
hsa_circRNA_025593	1.415466	8.464581	4.640675	0.001387	0.076441	-0.68157
hsa_circRNA_102060	1.412664	6.639022	2.593422	0.030199	0.180278	-3.68242
hsa_circRNA_104334	1.407054	7.770485	2.455352	0.037696	0.192648	-3.89405
hsa_circRNA_402239	1.406277	8.702386	7.016089	7.92E-05	0.032666	2.02152
hsa_circRNA_401798	1.397493	6.598058	2.950561	0.017076	0.154027	-3.13265
hsa_circRNA_102755	1.374609	8.965953	3.065583	0.014237	0.147937	-2.9561
hsa_circRNA_104333	1.369757	8.011437	2.293014	0.048929	0.21138	-4.14081
hsa_circRNA_001596	1.365427	11.65	2.977084	0.016373	0.153442	-3.09189
hsa_circRNA_401782	1.361063	10.74573	3.627419	0.005975	0.121817	-2.10814
hsa_circRNA_067938	1.357486	7.839328	3.304808	0.009794	0.137197	-2.5915
hsa_circRNA_005131	1.352576	7.863374	8.957045	1.23E-05	0.021781	3.648361
hsa_circRNA_005778	1.350836	9.464429	4.831145	0.001071	0.070472	-0.43144
hsa_circRNA_000760	1.349049	8.66101	2.91299	0.018124	0.155906	-3.19043
hsa_circRNA_101504	1.344595	10.19518	3.596896	0.006257	0.122886	-2.15337
hsa_circRNA_000487	1.343591	9.895952	7.52181	4.70E-05	0.03165	2.489458
hsa_circRNA_405944	1.335243	8.478176	4.703625	0.001273	0.075999	-0.59824
hsa_circRNA_404345	1.332396	7.454188	3.623627	0.006009	0.121817	-2.11375
hsa_circRNA_084527	1.329091	5.924578	7.021249	7.87E-05	0.032666	2.026467
hsa_circRNA_076989	1.326614	10.17666	5.802481	0.000312	0.053227	0.751229
hsa_circRNA_104332	1.322762	8.337084	2.298814	0.048476	0.210758	-4.13204
hsa_circRNA_101675	1.320076	7.188476	3.719933	0.005198	0.116014	-1.97174
hsa_circRNA_002120	1.311727	8.262182	5.497272	0.000453	0.05605	0.396213
hsa_circRNA_104664	1.310083	8.853947	5.736336	0.000337	0.054165	0.675557
hsa_circRNA_053339	1.309006	7.568365	6.709503	0.00011	0.03702	1.720965
hsa_circRNA_101364	1.308813	7.635752	6.686261	0.000113	0.03702	1.69764
hsa_circRNA_005990	1.303357	8.937575	4.891939	0.000988	0.069328	-0.35286
hsa_circRNA_104666	1.303097	9.838478	7.151184	6.87E-05	0.03165	2.149844
hsa_circRNA_077879	1.294123	9.255352	2.948421	0.017134	0.154027	-3.13594
hsa_circRNA_007250	1.293659	10.91104	6.083848	0.000223	0.047839	1.065429
hsa_circRNA_100279	1.286342	10.09403	7.028809	7.81E-05	0.032666	2.033709
hsa_circRNA_404623	1.281039	7.549462	5.719749	0.000344	0.054165	0.656471
hsa_circRNA_400817	1.27929	7.66079	7.801499	3.57E-05	0.030345	2.734135
hsa_circRNA_103312	1.27557	8.034958	4.436317	0.00184	0.084156	-0.95655
hsa_circRNA_000762	1.26624	7.155384	2.671847	0.02663	0.173369	-3.56179
hsa_circRNA_092535	1.260826	8.213793	4.067758	0.00311	0.101571	-1.46929
hsa_circRNA_101584	1.260406	9.580403	5.350865	0.000544	0.057445	0.220566
hsa_circRNA_100583	1.259768	9.216076	2.393093	0.041663	0.199326	-3.98901
hsa_circRNA_403730	1.256541	6.704869	3.667098	0.005627	0.120482	-2.04951
hsa_circRNA_068465	1.256313	9.301389	5.703531	0.000351	0.054264	0.637768

hsa_circRNA_013729	1.254059	11.05805	2.692732	0.025754	0.172546	-3.52964
hsa_circRNA_000286	1.248008	8.060093	5.554648	0.000422	0.055635	0.464097
hsa_circRNA_001565	1.234964	9.274018	4.583488	0.0015	0.078692	-0.75784
hsa_circRNA_102681	1.234056	7.869514	5.401256	0.000511	0.057403	0.281416
hsa_circRNA_101505	1.231819	9.915065	3.560764	0.00661	0.124312	-2.20706
hsa_circRNA_004672	1.231084	8.859822	3.370779	0.008844	0.134454	-2.49177
hsa_circRNA_104658	1.219144	7.048365	4.612411	0.001441	0.078124	-0.7192
hsa_circRNA_104667	1.216873	10.21433	6.220866	0.00019	0.045173	1.213998
hsa_circRNA_101314	1.214595	9.57638	6.567752	0.000129	0.03702	1.577501
hsa_circRNA_402961	1.206044	7.490331	5.799294	0.000313	0.053227	0.747599
hsa_circRNA_010884	1.199809	9.765995	4.303509	0.002218	0.087978	-1.13886
hsa_circRNA_032136	1.198412	7.565465	6.396415	0.000156	0.0416	1.400192
hsa_circRNA_403876	1.194825	9.485012	4.665893	0.00134	0.076324	-0.64811
hsa_circRNA_004559	1.194064	7.754885	5.668819	0.000366	0.054491	0.597595
hsa_circRNA_102680	1.191411	8.139713	4.775883	0.001154	0.073059	-0.5034
hsa_circRNA_001376	1.188289	7.100833	5.271264	0.000602	0.059114	0.123595
hsa_circRNA_405577	1.183762	7.38806	3.014033	0.015444	0.150655	-3.03515
hsa_circRNA_043397	1.181022	6.956757	2.440461	0.038609	0.19394	-3.9168
hsa_circRNA_064743	1.17572	7.838983	4.574997	0.001517	0.079169	-0.7692
hsa_circRNA_092572	1.175713	8.407343	5.885109	0.000282	0.053227	0.844785
hsa_circRNA_101538	1.172742	9.099601	6.970069	8.31E-05	0.03285	1.97724
hsa_circRNA_058097	1.167718	7.584681	4.391133	0.00196	0.086873	-1.01826
hsa_circRNA_072714	1.166257	5.517342	8.482869	1.87E-05	0.021781	3.291119
hsa_circRNA_102135	1.161714	8.79763	2.881957	0.01904	0.157891	-3.23819
hsa_circRNA_102027	1.158873	8.444676	5.543193	0.000428	0.055635	0.450587
hsa_circRNA_077007	1.157458	10.68263	3.018804	0.015328	0.150481	-3.02783
hsa_circRNA_007938	1.155968	7.129283	5.779293	0.00032	0.053854	0.72478
hsa_circRNA_100465	1.154317	6.331633	5.117589	0.000734	0.061699	-0.06656
hsa_circRNA_090122	1.151063	9.284924	3.501688	0.007233	0.127682	-2.29517
hsa_circRNA_031147	1.14392	9.220479	5.624312	0.000387	0.054491	0.545803
hsa_circRNA_102137	1.143702	8.092708	3.266237	0.010399	0.138473	-2.64998
hsa_circRNA_092556	1.139569	12.03053	2.769369	0.022783	0.166885	-3.4116
hsa_circRNA_102526	1.137915	8.672062	3.331171	0.009402	0.136341	-2.5516
hsa_circRNA_061036	1.13755	7.833818	5.833369	0.0003	0.053227	0.786329
hsa_circRNA_000464	1.13682	8.841004	3.969416	0.00359	0.103366	-1.6096
hsa_circRNA_100313	1.135824	10.78642	2.670565	0.026685	0.173369	-3.56377
hsa_circRNA_023339	1.135498	9.632487	5.301041	0.00058	0.059114	0.159992
hsa_circRNA_103777	1.13288	8.137482	3.006352	0.015632	0.151182	-3.04694
hsa_circRNA_060762	1.132413	7.285981	5.45146	0.000479	0.056863	0.341627
hsa_circRNA_104289	1.129527	7.497046	5.148988	0.000705	0.061259	-0.02739
hsa_circRNA_075386	1.126683	8.431634	6.032049	0.000237	0.048909	1.008513
hsa_circRNA_103554	1.125595	8.280443	4.373273	0.00201	0.087152	-1.04274
hsa_circRNA_403839	1.123162	9.366872	6.211409	0.000192	0.045173	1.203836

hsa_circRNA_090302	1.120426	7.412071	2.548358	0.032464	0.18465	-3.75162
hsa_circRNA_001352	1.116932	7.323458	2.824004	0.020881	0.161923	-3.32743
hsa_circRNA_105040	1.114866	7.68117	3.96956	0.003589	0.103366	-1.6094
hsa_circRNA_102532	1.11391	8.016269	3.275733	0.010247	0.138146	-2.63557
hsa_circRNA_104031	1.109376	9.243531	4.086844	0.003026	0.101084	-1.44222
hsa_circRNA_102733	1.108037	8.250945	5.189371	0.000669	0.060962	0.022744
hsa_circRNA_101541	1.104894	8.705993	7.336631	5.67E-05	0.03165	2.322017
hsa_circRNA_101436	1.102588	9.050176	3.87867	0.004103	0.107364	-1.74033
hsa_circRNA_101542	1.101949	8.546434	6.322923	0.000169	0.042833	1.322805
hsa_circRNA_405609	1.101735	8.041887	2.670127	0.026703	0.173369	-3.56444
hsa_circRNA_038288	1.099779	9.140109	2.48917	0.035703	0.188508	-3.84233
hsa_circRNA_002491	1.096098	8.389022	2.822843	0.02092	0.16213	-3.32922
hsa_circRNA_406106	1.094662	8.86469	3.605321	0.006178	0.122614	-2.14088
hsa_circRNA_100745	1.089757	7.504431	5.062975	0.000788	0.063884	-0.13508
hsa_circRNA_405330	1.089459	10.20351	3.195418	0.011613	0.142223	-2.75769
hsa_circRNA_102529	1.088381	7.036923	3.391726	0.008563	0.13326	-2.4602
hsa_circRNA_101475	1.087579	7.21207	4.844949	0.001052	0.070198	-0.41354
hsa_circRNA_100801	1.086356	7.656221	7.139062	6.96E-05	0.03165	2.138431
hsa_circRNA_014352	1.086276	10.44109	5.442737	0.000485	0.056863	0.331195
hsa_circRNA_101790	1.086149	9.308648	3.499469	0.007258	0.127682	-2.29849
hsa_circRNA_102737	1.084148	8.915412	3.986206	0.003503	0.103366	-1.58555
hsa_circRNA_405113	1.079776	9.007327	4.419808	0.001883	0.084325	-0.97906
hsa_circRNA_405138	1.075846	7.483667	5.624284	0.000387	0.054491	0.54577
hsa_circRNA_043509	1.073652	7.441534	3.876628	0.004116	0.107364	-1.74328
hsa_circRNA_068601	1.072353	8.049522	4.610164	0.001446	0.078124	-0.72219
hsa_circRNA_100786	1.070854	8.115699	5.277376	0.000598	0.059114	0.131078
hsa_circRNA_400729	1.07062	6.875925	6.632871	0.00012	0.03702	1.643767
hsa_circRNA_102517	1.06448	7.632623	2.545847	0.032595	0.18469	-3.75547
hsa_circRNA_403802	1.060705	7.700706	3.035976	0.014918	0.149261	-3.00148
hsa_circRNA_101363	1.060547	7.600524	5.098186	0.000753	0.062505	-0.09085
hsa_circRNA_406030	1.058828	7.319009	6.095381	0.00022	0.047839	1.078046
hsa_circRNA_102525	1.056996	8.509538	2.950879	0.017067	0.154027	-3.13216
hsa_circRNA_104137	1.054498	11.18441	5.385067	0.000521	0.057445	0.261913
hsa_circRNA_104185	1.054178	6.780983	3.431965	0.008048	0.130929	-2.39966
hsa_circRNA_073248	1.053142	9.292059	6.161918	0.000204	0.047021	1.150432
hsa_circRNA_101539	1.052277	8.936756	6.699671	0.000111	0.03702	1.711107
hsa_circRNA_102678	1.051908	7.973485	4.251365	0.002388	0.090927	-1.2112
hsa_circRNA_000287	1.051596	7.8539	5.841025	0.000297	0.053227	0.795005
hsa_circRNA_002793	1.051063	7.639407	4.965042	0.000896	0.066036	-0.25919
hsa_circRNA_405627	1.046336	8.655516	4.077051	0.003069	0.101103	-1.4561
hsa_circRNA_017215	1.046136	9.343968	3.874041	0.004132	0.107364	-1.74703
hsa_circRNA_042965	1.045578	7.843217	4.871122	0.001016	0.069488	-0.3797
hsa_circRNA_102683	1.043977	8.383217	5.170227	0.000686	0.060962	-0.00099

hsa_circRNA_103499	1.042375	8.826452	3.617414	0.006066	0.122185	-2.12295
hsa_circRNA_027093	1.041637	9.149997	4.239353	0.00243	0.090927	-1.22793
hsa_circRNA_102558	1.040799	7.833265	4.644776	0.001379	0.076441	-0.67612
hsa_circRNA_001814	1.039682	6.706931	3.308948	0.009732	0.136896	-2.58523
hsa_circRNA_020273	1.038731	6.624271	5.613362	0.000392	0.054491	0.533012
hsa_circRNA_044248	1.038285	8.274045	2.357645	0.044105	0.203587	-4.04291
hsa_circRNA_030162	1.036062	11.80025	7.136897	6.97E-05	0.03165	2.13639
hsa_circRNA_104257	1.035956	6.348521	4.687435	0.001301	0.076324	-0.61961
hsa_circRNA_100747	1.035576	7.425594	5.165954	0.000689	0.060962	-0.0063
hsa_circRNA_044879	1.035028	7.612791	3.064475	0.014262	0.147937	-2.95779
hsa_circRNA_001588	1.033431	11.39213	2.285651	0.049511	0.21208	-4.15192
hsa_circRNA_100311	1.0333	11.37188	2.832003	0.020616	0.161064	-3.31511
hsa_circRNA_000120	1.0313	10.53504	2.99151	0.016003	0.152015	-3.06973
hsa_circRNA_086474	1.028398	10.63243	5.25855	0.000612	0.059531	0.108011
hsa_circRNA_102807	1.026593	9.334739	4.728389	0.00123	0.075101	-0.56564
hsa_circRNA_092364	1.026513	8.966483	5.695422	0.000355	0.054264	0.6284
hsa_circRNA_104843	1.026211	6.100428	2.506016	0.034749	0.186658	-3.81654
hsa_circRNA_000164	1.023719	7.711346	6.575273	0.000128	0.03702	1.585186
hsa_circRNA_102677	1.023317	8.117837	4.689362	0.001297	0.076324	-0.61707
hsa_circRNA_101083	1.021995	7.837875	4.521211	0.001635	0.081329	-0.8415
hsa_circRNA_102604	1.021811	9.144057	5.429634	0.000493	0.056863	0.315501
hsa_circRNA_005751	1.021642	7.748496	2.525506	0.033678	0.185229	-3.78667
hsa_circRNA_400742	1.021229	7.882228	6.553389	0.000131	0.03702	1.562803
hsa_circRNA_009181	1.02096	9.02385	4.433146	0.001848	0.084156	-0.96087
hsa_circRNA_102560	1.020649	8.730172	6.648837	0.000118	0.03702	1.65992
hsa_circRNA_404517	1.013537	8.283108	4.466391	0.001764	0.082988	-0.91566
hsa_circRNA_100628	1.012061	6.165813	4.756504	0.001185	0.073988	-0.52875
hsa_circRNA_101474	1.011857	7.895041	3.225729	0.011076	0.141373	-2.71154
hsa_circRNA_003940	1.011561	8.393219	5.620026	0.000389	0.054491	0.540799
hsa_circRNA_402986	1.010608	7.845948	5.19013	0.000668	0.060962	0.023684
hsa_circRNA_406034	1.008543	6.468444	5.016478	0.000838	0.065182	-0.19381
hsa_circRNA_405767	1.007091	6.558396	3.205117	0.011438	0.142223	-2.74292
hsa_circRNA_400038	1.006177	7.891378	3.753045	0.004946	0.114933	-1.92319
hsa_circRNA_104032	1.003256	10.15073	3.484431	0.007426	0.128657	-2.32099
hsa_circRNA_101506	1.002656	9.26931	3.30799	0.009746	0.136958	-2.58668
hsa_circRNA_004926	1.002495	6.567243	4.141246	0.002797	0.097153	-1.36537
hsa_circRNA_100537	1.000709	8.481254	2.713051	0.02493	0.170623	-3.49835
hsa_circRNA_104272	1.000543	6.52437	6.304156	0.000173	0.042833	1.302915
hsa_circRNA_092377	1.000123	9.656752	5.513683	0.000444	0.055894	0.415684
hsa_circRNA_023908	1.000006	9.333585	3.099386	0.0135	0.146343	-2.90434
hsa_circRNA_403556	-1.0057	8.802231	-2.30207	0.048223	0.21028	-4.12712
hsa_circRNA_100778	-1.0095	7.883487	-3.14811	0.012506	0.144804	-2.82986
hsa_circRNA_101001	-1.06163	7.943691	-3.71949	0.005201	0.116014	-1.97239

hsa_circRNA_000139	-1.06709	8.522123	-2.33249	0.045924	0.206656	-4.08107
hsa_circRNA_407246	-1.23446	6.955515	-3.25472	0.010587	0.139155	-2.66748
hsa_circRNA_077736	-1.26321	7.355031	-2.42087	0.039844	0.196458	-3.94669
hsa_circRNA_017549	-1.46598	7.70873	-3.42636	0.008118	0.131425	-2.40808
hsa_circRNA_061825	-2.2123	7.945972	-8.82832	1.37E-05	0.021781	3.553717

Table S3. The differentially expressed circRNAs in GSE93541 microarray dataset

circRNAs ID	logFC	AveExpr	t	P.Value	adj.P.Val	B
hsa_circRNA_104902	5.549918	6.395979	6.409715	0.000719	0.009633	-0.14517
hsa_circRNA_104081	5.379094	5.197306	17.24891	2.77E-06	0.000693	5.564941
hsa_circRNA_100647	5.127569	5.844993	6.832409	0.000513	0.008468	0.225974
hsa_circRNA_000082	5.056468	5.945115	15.15818	5.86E-06	0.000888	4.871626
hsa_circRNA_100883	5.04177	9.082862	3.292856	0.016899	0.066209	-3.6126
hsa_circRNA_101522	5.022606	7.054242	5.453828	0.001655	0.015685	-1.06337
hsa_circRNA_001547	4.80241	5.639542	9.82981	6.96E-05	0.002937	2.378418
hsa_circRNA_100560	4.762872	5.632243	6.575831	0.000628	0.009319	0.003076
hsa_circRNA_104986	4.451131	5.761801	5.090469	0.002337	0.018623	-1.44395
hsa_circRNA_103814	4.434023	5.681234	12.42768	1.84E-05	0.001581	3.752427
hsa_circRNA_104907	4.411472	6.16879	4.739302	0.003316	0.02341	-1.82964
hsa_circRNA_101704	4.395244	4.841887	12.5625	1.73E-05	0.001581	3.814544
hsa_circRNA_400004	4.301286	5.152855	8.995348	0.000114	0.003639	1.851741
hsa_circRNA_001459	4.26431	7.651127	10.54959	4.67E-05	0.002407	2.796221
hsa_circRNA_100748	4.212109	5.611901	5.204993	0.002093	0.0175	-1.32201
hsa_circRNA_101412	4.040645	8.636371	14.78575	6.77E-06	0.000912	4.734631
hsa_circRNA_102922	3.902368	5.945268	3.220858	0.018479	0.070956	-3.7092
hsa_circRNA_103002	3.770017	5.591002	3.919177	0.00803	0.041848	-2.80226
hsa_circRNA_100719	3.758885	7.201555	3.067254	0.022419	0.08094	-3.91737
hsa_circRNA_103212	3.757863	5.944571	5.078194	0.002365	0.018741	-1.45713
hsa_circRNA_104038	3.750359	5.576448	3.247928	0.017866	0.06952	-3.67281
hsa_circRNA_102415	3.710927	5.940576	3.663015	0.010807	0.050461	-3.12689
hsa_circRNA_101405	3.679184	5.934563	6.552603	0.00064	0.009319	-0.01746
hsa_circRNA_100040	3.524407	7.962445	2.528365	0.045328	0.131188	-4.6651
hsa_circRNA_104694	3.439457	6.196708	3.736609	0.009913	0.047739	-3.03263
hsa_circRNA_101839	3.432159	7.205079	2.577043	0.042471	0.126046	-4.5968
hsa_circRNA_101305	3.376097	6.7664	5.641945	0.001393	0.014186	-0.87343
hsa_circRNA_100115	3.3682	6.192718	2.830451	0.030404	0.100637	-4.2432
hsa_circRNA_105019	3.252407	6.251331	3.735645	0.009924	0.047739	-3.03386
hsa_circRNA_102082	3.24236	10.65089	18.95479	1.60E-06	0.000693	6.048886
hsa_circRNA_101427	3.219474	6.557064	2.569878	0.042879	0.126827	-4.60685
hsa_circRNA_102379	3.217535	6.537761	2.9668	0.025487	0.087898	-4.05493
hsa_circRNA_400027	3.17686	9.874688	8.289287	0.00018	0.004916	1.366021
hsa_circRNA_101404	3.161732	8.148588	5.304436	0.001904	0.016742	-1.21762
hsa_circRNA_102046	3.155135	7.212284	3.1167	0.021059	0.077792	-3.85006
hsa_circRNA_101753	3.111119	9.202917	6.495486	0.00067	0.009544	-0.06823
hsa_circRNA_104029	3.080117	7.235535	5.611234	0.001433	0.014419	-0.90412
hsa_circRNA_100879	3.058006	5.884435	3.614837	0.011441	0.052219	-3.18902
hsa_circRNA_104110	2.96896	7.195974	2.516925	0.046028	0.132558	-4.68117
hsa_circRNA_000855	2.869953	8.892158	13.8434	9.90E-06	0.001155	4.367214
hsa_circRNA_100606	2.864535	6.175698	3.420949	0.014442	0.060498	-3.4424

hsa_circRNA_104381	2.794117	6.041408	3.374345	0.015287	0.062477	-3.50408
hsa_circRNA_102113	2.747358	9.828945	6.972381	0.00046	0.008143	0.344562
hsa_circRNA_104374	2.718753	7.109213	3.429679	0.01429	0.060147	-3.43088
hsa_circRNA_103727	2.690488	7.430931	2.583528	0.042105	0.125602	-4.58771
hsa_circRNA_103929	2.645205	7.851223	6.03847	0.000982	0.011505	-0.4882
hsa_circRNA_101945	2.606853	6.603082	2.456955	0.049892	0.139777	-4.76541
hsa_circRNA_101092	2.572469	7.439534	4.400894	0.00472	0.02941	-2.21861
hsa_circRNA_000200	2.561462	10.34396	10.91588	3.85E-05	0.002248	2.997103
hsa_circRNA_102600	2.559545	8.66866	6.534364	0.00065	0.009324	-0.03364
hsa_circRNA_102445	2.525952	12.84816	7.347545	0.000347	0.007026	0.652389
hsa_circRNA_103012	2.51223	7.13617	3.037901	0.023272	0.082823	-3.95746
hsa_circRNA_100799	2.474349	6.72164	3.309981	0.016545	0.065544	-3.58973
hsa_circRNA_103749	2.403072	9.640275	9.073915	0.000109	0.003571	1.903423
hsa_circRNA_102441	2.387629	6.912386	3.448285	0.013971	0.059664	-3.40636
hsa_circRNA_104310	2.385998	12.74989	10.21541	5.61E-05	0.002649	2.606182
hsa_circRNA_104050	2.374597	11.27209	11.01411	3.66E-05	0.002211	3.049702
hsa_circRNA_102399	2.369437	6.953069	4.525015	0.004139	0.026831	-2.07394
hsa_circRNA_103723	2.348896	9.95965	4.821452	0.003051	0.022073	-1.7378
hsa_circRNA_104313	2.299962	8.672599	3.483551	0.013387	0.058166	-3.36002
hsa_circRNA_104635	2.297165	7.141666	4.340896	0.005033	0.03039	-2.28938
hsa_circRNA_101407	2.285528	8.52755	3.983108	0.007468	0.039726	-2.72277
hsa_circRNA_002086	2.281682	10.26546	7.192589	0.000389	0.007434	0.526978
hsa_circRNA_102838	2.272879	11.90226	9.728673	7.38E-05	0.003004	2.317109
hsa_circRNA_101943	2.247856	9.913325	5.235861	0.002032	0.017284	-1.28945
hsa_circRNA_100888	2.20019	6.624833	2.756098	0.033509	0.107104	-4.34652
hsa_circRNA_100104	2.160606	7.361002	9.30313	9.48E-05	0.003457	2.051645
hsa_circRNA_102397	2.159984	7.640676	5.576219	0.001479	0.014716	-0.93926
hsa_circRNA_400040	2.136656	12.88406	7.942023	0.000227	0.005605	1.112159
hsa_circRNA_101440	2.111057	7.082708	6.419524	0.000713	0.009633	-0.13633
hsa_circRNA_102810	2.102392	8.016356	6.76901	0.000539	0.008688	0.17157
hsa_circRNA_104513	2.098685	9.905888	11.38645	3.03E-05	0.002041	3.244425
hsa_circRNA_000881	2.083647	12.82592	6.744176	0.000549	0.008688	0.15014
hsa_circRNA_101306	2.057336	7.418293	3.402454	0.014771	0.061289	-3.46684
hsa_circRNA_104099	2.051506	11.38357	5.126079	0.002258	0.018217	-1.40583
hsa_circRNA_000993	2.048863	10.10631	6.32799	0.000769	0.010052	-0.21928
hsa_circRNA_102631	2.039795	9.789093	8.587488	0.000148	0.004409	1.575959
hsa_circRNA_000042	2.030713	9.477708	5.87801	0.001129	0.012205	-0.64167
hsa_circRNA_102442	2.025088	10.51807	6.478702	0.00068	0.009597	-0.08322
hsa_circRNA_100984	2.015618	6.147512	2.636617	0.039234	0.119695	-4.51334
hsa_circRNA_101887	1.98106	8.107677	3.076099	0.022169	0.080534	-3.90531
hsa_circRNA_100882	1.97699	9.771699	10.4427	4.95E-05	0.002463	2.736155
hsa_circRNA_103085	1.96987	9.091516	2.466847	0.049232	0.138593	-4.75151
hsa_circRNA_102374	1.969544	10.47162	10.74827	4.21E-05	0.002361	2.90612

hsa_circRNA_102446	1.957439	9.064083	5.213382	0.002076	0.0175	-1.31315
hsa_circRNA_001040	1.946104	10.31297	8.34328	0.000173	0.004819	1.404573
hsa_circRNA_100997	1.93874	8.407016	4.733866	0.003334	0.023446	-1.83576
hsa_circRNA_103801	1.922821	10.50599	5.437671	0.00168	0.015685	-1.0799
hsa_circRNA_104200	1.903873	8.170936	10.1695	5.75E-05	0.002649	2.57955
hsa_circRNA_100891	1.890809	7.900134	4.295685	0.005285	0.031476	-2.34307
hsa_circRNA_001389	1.887989	9.553138	6.152423	0.000891	0.01099	-0.38114
hsa_circRNA_101373	1.866249	9.060177	9.055832	0.00011	0.003571	1.891568
hsa_circRNA_002039	1.848158	7.409816	3.042865	0.023125	0.082469	-3.95067
hsa_circRNA_102385	1.846646	8.961994	6.10549	0.000928	0.011124	-0.42504
hsa_circRNA_103456	1.825122	7.376121	6.365996	0.000745	0.009814	-0.18472
hsa_circRNA_102728	1.823128	7.650097	5.421706	0.001705	0.015685	-1.09628
hsa_circRNA_102171	1.806955	7.624583	3.863973	0.008554	0.043794	-2.87139
hsa_circRNA_104864	1.805437	7.947371	7.151953	0.000401	0.007556	0.49369
hsa_circRNA_100859	1.804105	6.697952	3.925658	0.007971	0.041789	-2.79417
hsa_circRNA_001264	1.799789	8.13657	5.152445	0.002201	0.0179	-1.37773
hsa_circRNA_104616	1.790622	9.233678	3.125772	0.020819	0.077397	-3.83774
hsa_circRNA_103137	1.78664	11.03265	7.491054	0.000312	0.00674	0.766429
hsa_circRNA_104510	1.784374	8.471294	4.276394	0.005397	0.032032	-2.36607
hsa_circRNA_101030	1.782183	7.740915	4.346468	0.005003	0.030313	-2.28278
hsa_circRNA_101063	1.777336	6.871707	4.77353	0.003202	0.02298	-1.79126
hsa_circRNA_000911	1.768351	13.1492	7.655017	0.000278	0.006345	0.894306
hsa_circRNA_000046	1.766822	9.317355	3.238793	0.01807	0.069973	-3.68508
hsa_circRNA_103608	1.752198	10.18141	5.750829	0.001264	0.013093	-0.76564
hsa_circRNA_000864	1.748896	12.12696	7.952063	0.000226	0.005605	1.119645
hsa_circRNA_104401	1.743464	10.99593	6.545159	0.000644	0.009319	-0.02406
hsa_circRNA_104148	1.741904	7.869837	3.439677	0.014117	0.059864	-3.4177
hsa_circRNA_101136	1.739729	8.604194	6.83632	0.000511	0.008468	0.229317
hsa_circRNA_100397	1.724886	8.180239	6.140527	0.0009	0.011024	-0.39225
hsa_circRNA_102041	1.721285	7.5571	3.814437	0.009056	0.044825	-2.93382
hsa_circRNA_100117	1.708388	8.764601	6.566486	0.000633	0.009319	-0.00518
hsa_circRNA_101248	1.701494	8.386806	4.661306	0.003592	0.024821	-1.91777
hsa_circRNA_102045	1.701277	8.809551	4.882579	0.002869	0.02102	-1.67011
hsa_circRNA_102551	1.690221	6.940558	3.819851	0.008999	0.044767	-2.92698
hsa_circRNA_100470	1.689887	8.138617	2.817272	0.030931	0.101804	-4.26148
hsa_circRNA_104803	1.66898	7.422129	2.972662	0.025296	0.087898	-4.04687
hsa_circRNA_100048	1.668478	7.501766	3.155445	0.020056	0.075479	-3.79751
hsa_circRNA_100832	1.663845	7.402915	4.547322	0.004043	0.026689	-2.04819
hsa_circRNA_103554	1.661797	9.150712	5.534251	0.001537	0.015036	-0.98159
hsa_circRNA_102924	1.659112	9.018112	6.427195	0.000709	0.009633	-0.12942
hsa_circRNA_001405	1.645692	11.30917	5.729694	0.001288	0.013264	-0.78644
hsa_circRNA_100422	1.644272	9.714228	8.530783	0.000153	0.004409	1.536592
hsa_circRNA_103555	1.643711	12.24415	5.441088	0.001675	0.015685	-1.0764

hsa_circRNA_000684	1.623346	10.10367	4.778571	0.003186	0.022957	-1.78562
hsa_circRNA_103134	1.601497	10.64736	4.602007	0.003819	0.026061	-1.98538
hsa_circRNA_001241	1.591908	10.56678	4.936834	0.002718	0.020427	-1.61048
hsa_circRNA_101861	1.586486	8.50022	4.937019	0.002718	0.020427	-1.61028
hsa_circRNA_100446	1.567078	7.335682	4.437371	0.00454	0.028906	-2.17585
hsa_circRNA_104400	1.546802	10.93791	8.206657	0.00019	0.005115	1.306551
hsa_circRNA_104139	1.546641	6.736447	3.296612	0.01682	0.066185	-3.60758
hsa_circRNA_100147	1.540636	8.714112	4.40979	0.004675	0.029236	-2.20817
hsa_circRNA_001654	1.536069	10.97618	7.460572	0.000319	0.00674	0.742373
hsa_circRNA_104126	1.522054	10.49393	6.912142	0.000482	0.008354	0.293782
hsa_circRNA_101525	1.498969	13.44098	5.149154	0.002208	0.0179	-1.38123
hsa_circRNA_104643	1.482877	7.272089	2.831021	0.030381	0.100637	-4.24241
hsa_circRNA_100694	1.466876	6.690825	3.053551	0.022813	0.081982	-3.93607
hsa_circRNA_100827	1.461519	7.837549	8.051257	0.000211	0.00543	1.193136
hsa_circRNA_101706	1.46062	9.260293	4.021752	0.00715	0.038881	-2.67502
hsa_circRNA_103687	1.432145	7.093633	4.200408	0.005863	0.034108	-2.45723
hsa_circRNA_001175	1.423593	10.60086	3.84717	0.00872	0.044131	-2.89253
hsa_circRNA_101656	1.419755	11.08722	4.366464	0.004897	0.030149	-2.25915
hsa_circRNA_103825	1.41671	6.811039	3.355696	0.01564	0.063248	-3.52884
hsa_circRNA_101368	1.394656	6.924927	3.097359	0.02158	0.078721	-3.87636
hsa_circRNA_001587	1.390826	9.03649	3.649094	0.010986	0.050756	-3.14481
hsa_circRNA_104598	1.388693	10.44453	6.00031	0.001015	0.011625	-0.52441
hsa_circRNA_102292	1.376125	8.146184	4.411252	0.004668	0.029236	-2.20645
hsa_circRNA_101282	1.375793	9.981873	4.838632	0.002998	0.021785	-1.71872
hsa_circRNA_000997	1.36037	6.992552	2.459026	0.049753	0.139611	-4.7625
hsa_circRNA_104342	1.354903	10.85889	3.999711	0.00733	0.03927	-2.70223
hsa_circRNA_103791	1.340246	7.224721	3.775561	0.009473	0.046325	-2.98306
hsa_circRNA_001409	1.33815	8.381732	3.532325	0.012624	0.055782	-3.29621
hsa_circRNA_102774	1.331207	8.668461	4.670967	0.003556	0.024709	-1.90681
hsa_circRNA_101589	1.326484	8.973969	3.524785	0.012739	0.056045	-3.30605
hsa_circRNA_105013	1.316627	9.664067	6.190144	0.000863	0.010797	-0.34605
hsa_circRNA_104193	1.30487	9.672484	6.552133	0.00064	0.009319	-0.01788
hsa_circRNA_001350	1.30269	10.30822	3.601163	0.011628	0.052612	-3.20672
hsa_circRNA_101957	1.301071	9.336284	4.149038	0.006204	0.035273	-2.51935
hsa_circRNA_101788	1.300164	7.153486	2.793016	0.031926	0.103716	-4.29517
hsa_circRNA_000104	1.284464	8.26609	4.062848	0.006828	0.037787	-2.62449
hsa_circRNA_104315	1.276183	12.72203	7.412069	0.000331	0.006899	0.703911
hsa_circRNA_101905	1.269697	9.100077	4.234676	0.005647	0.033073	-2.41601
hsa_circRNA_104251	1.267408	7.504704	3.877059	0.008426	0.043395	-2.85496
hsa_circRNA_102690	1.264224	9.281949	2.686031	0.036748	0.113887	-4.44425
hsa_circRNA_101308	1.257923	7.051478	2.481414	0.048276	0.136341	-4.73104
hsa_circRNA_001109	1.25478	9.112412	3.169179	0.019713	0.074712	-3.77893
hsa_circRNA_101695	1.244113	10.05227	5.054149	0.002421	0.01906	-1.48301

hsa_circRNA_101674	1.243521	8.317972	4.061168	0.006841	0.037787	-2.62655
hsa_circRNA_102741	1.241902	10.31034	5.957322	0.001054	0.011903	-0.56541
hsa_circRNA_101958	1.235182	11.45281	6.155444	0.000889	0.01099	-0.37833
hsa_circRNA_100230	1.2137	9.775942	5.412662	0.00172	0.015685	-1.10557
hsa_circRNA_001240	1.211232	7.753097	3.773319	0.009498	0.046325	-2.98591
hsa_circRNA_400103	1.210608	8.642583	3.47437	0.013536	0.058669	-3.37207
hsa_circRNA_104652	1.20762	9.421423	5.929225	0.00108	0.011995	-0.59234
hsa_circRNA_102359	1.205974	13.43588	3.907293	0.00814	0.042293	-2.8171
hsa_circRNA_103444	1.195837	12.61765	5.429577	0.001693	0.015685	-1.0882
hsa_circRNA_103211	1.194055	10.38249	3.427464	0.014328	0.060164	-3.4338
hsa_circRNA_000644	1.193201	7.652275	2.744375	0.034029	0.107583	-4.36285
hsa_circRNA_101648	1.192601	7.789033	2.666641	0.037703	0.116228	-4.47134
hsa_circRNA_104075	1.191124	9.184365	4.518268	0.004168	0.026831	-2.08175
hsa_circRNA_101501	1.188039	8.639603	4.316378	0.005168	0.031092	-2.31846
hsa_circRNA_101914	1.187483	9.853515	2.76897	0.032948	0.106245	-4.3286
hsa_circRNA_102747	1.185859	8.206335	2.96639	0.025501	0.087898	-4.05549
hsa_circRNA_101881	1.178999	8.040391	4.710354	0.003415	0.02392	-1.86225
hsa_circRNA_101058	1.17664	7.245171	3.209569	0.018741	0.071649	-3.72441
hsa_circRNA_002008	1.173511	7.118944	2.655801	0.038248	0.117495	-4.4865
hsa_circRNA_103832	1.163886	7.017759	6.034563	0.000986	0.011505	-0.4919
hsa_circRNA_001143	1.163505	8.328367	2.550891	0.043981	0.128997	-4.63349
hsa_circRNA_103627	1.154528	9.772016	3.813825	0.009062	0.044825	-2.93459
hsa_circRNA_103887	1.141187	9.059462	4.52134	0.004155	0.026831	-2.07819
hsa_circRNA_000926	1.138317	9.638092	3.348959	0.01577	0.063439	-3.53779
hsa_circRNA_000671	1.114389	9.688598	5.777035	0.001235	0.012867	-0.73993
hsa_circRNA_104134	1.091884	10.88248	4.553476	0.004017	0.026643	-2.0411
hsa_circRNA_102331	1.084962	8.407386	2.598365	0.041281	0.123772	-4.56691
hsa_circRNA_000780	1.082441	9.894931	3.85503	0.008642	0.044117	-2.88264
hsa_circRNA_000942	1.080112	8.723235	4.410144	0.004673	0.029236	-2.20775
hsa_circRNA_103089	1.078741	8.170922	4.54238	0.004064	0.026689	-2.05389
hsa_circRNA_104203	1.077142	8.251745	3.015077	0.023959	0.084925	-3.98869
hsa_circRNA_000250	1.07426	8.183096	4.973196	0.002622	0.020225	-1.57076
hsa_circRNA_101711	1.066344	9.122374	4.596689	0.00384	0.026061	-1.99147
hsa_circRNA_101689	1.060893	7.882647	5.201985	0.002099	0.0175	-1.32518
hsa_circRNA_000638	1.052596	8.327637	5.051665	0.002427	0.01906	-1.48569
hsa_circRNA_100122	1.041117	7.991761	3.390722	0.014984	0.06173	-3.48237
hsa_circRNA_102093	1.033845	7.712946	2.535619	0.044889	0.130236	-4.65492
hsa_circRNA_103288	1.026486	7.521128	4.044916	0.006967	0.03836	-2.64651
hsa_circRNA_103405	1.019378	10.76383	3.83794	0.008814	0.044257	-2.90415
hsa_circRNA_101969	1.015857	8.408336	4.844794	0.00298	0.02174	-1.71189
hsa_circRNA_104880	1.014573	7.882902	2.806501	0.031369	0.102284	-4.27643
hsa_circRNA_104905	1.004297	8.665074	2.575417	0.042563	0.126106	-4.59908
hsa_circRNA_102393	-1.00187	11.1049	-5.15577	0.002194	0.0179	-1.37419

hsa_circRNA_101201	-1.02233	11.95817	-5.95969	0.001052	0.011903	-0.56314
hsa_circRNA_101408	-1.02541	9.387605	-6.01632	0.001001	0.01161	-0.5092
hsa_circRNA_100492	-1.03145	12.18291	-4.96423	0.002645	0.020227	-1.58054
hsa_circRNA_000868	-1.03705	8.141597	-2.71573	0.035336	0.110884	-4.40279
hsa_circRNA_000481	-1.04014	10.98471	-5.5206	0.001556	0.015141	-0.99541
hsa_circRNA_103781	-1.04793	11.19655	-5.28046	0.001947	0.016881	-1.24266
hsa_circRNA_102567	-1.05015	8.264362	-4.03415	0.007051	0.038583	-2.65976
hsa_circRNA_101287	-1.05462	9.687434	-5.45592	0.001652	0.015685	-1.06122
hsa_circRNA_101766	-1.05857	9.778505	-5.91011	0.001098	0.012066	-0.61072
hsa_circRNA_102034	-1.07623	10.16392	-5.84203	0.001166	0.01252	-0.67654
hsa_circRNA_000987	-1.08204	11.05607	-3.60208	0.011615	0.052612	-3.20553
hsa_circRNA_103589	-1.08235	7.879965	-3.46601	0.013674	0.058992	-3.38305
hsa_circRNA_103348	-1.08857	10.30148	-4.29785	0.005273	0.031476	-2.34049
hsa_circRNA_101227	-1.09357	9.668417	-6.46623	0.000687	0.009618	-0.09438
hsa_circRNA_100908	-1.09487	8.184905	-3.34114	0.015922	0.063799	-3.54819
hsa_circRNA_103757	-1.098	8.028276	-3.00566	0.02425	0.085607	-4.00159
hsa_circRNA_103488	-1.0983	8.498409	-2.9906	0.024721	0.086748	-4.02225
hsa_circRNA_100089	-1.09867	7.422387	-2.94615	0.026173	0.089861	-4.08333
hsa_circRNA_000956	-1.10251	12.80335	-5.18747	0.002128	0.017577	-1.34054
hsa_circRNA_103605	-1.10591	10.40807	-4.35562	0.004954	0.030149	-2.27196
hsa_circRNA_104268	-1.10781	11.16026	-6.20318	0.000854	0.010797	-0.33397
hsa_circRNA_100565	-1.11961	9.057929	-5.54401	0.001523	0.015036	-0.97172
hsa_circRNA_104756	-1.12887	12.86055	-4.52238	0.00415	0.026831	-2.077
hsa_circRNA_102033	-1.14035	9.089415	-5.78679	0.001224	0.012832	-0.73037
hsa_circRNA_400064	-1.14156	10.1787	-5.29971	0.001912	0.016742	-1.22254
hsa_circRNA_400014	-1.14637	7.829811	-3.99922	0.007334	0.03927	-2.70284
hsa_circRNA_100754	-1.1492	9.292497	-3.84759	0.008716	0.044131	-2.892
hsa_circRNA_102439	-1.15069	8.581893	-3.68681	0.010508	0.049668	-3.09633
hsa_circRNA_100367	-1.15311	13.35649	-6.84915	0.000506	0.008468	0.240265
hsa_circRNA_102432	-1.15813	6.945872	-4.54101	0.00407	0.026689	-2.05547
hsa_circRNA_100360	-1.16261	7.534079	-3.21789	0.018547	0.071064	-3.7132
hsa_circRNA_103297	-1.16424	6.731778	-4.5792	0.003911	0.026336	-2.01152
hsa_circRNA_102707	-1.16627	8.300597	-5.08933	0.00234	0.018623	-1.44517
hsa_circRNA_102605	-1.17509	8.166445	-2.88883	0.028183	0.095268	-4.16238
hsa_circRNA_102061	-1.1754	13.4	-5.99964	0.001016	0.011625	-0.52504
hsa_circRNA_102894	-1.17702	7.132943	-4.31335	0.005185	0.031092	-2.32205
hsa_circRNA_104495	-1.18065	10.77874	-5.36537	0.001798	0.016142	-1.15433
hsa_circRNA_101527	-1.18942	6.675447	-3.72306	0.010071	0.04805	-3.04992
hsa_circRNA_103148	-1.19361	10.51198	-4.25223	0.00554	0.032554	-2.39496
hsa_circRNA_102414	-1.19655	9.258725	-4.92137	0.00276	0.020566	-1.62743
hsa_circRNA_103164	-1.20519	10.50857	-6.97498	0.000459	0.008143	0.346747
hsa_circRNA_104475	-1.21024	10.48257	-6.04457	0.000977	0.011505	-0.48243
hsa_circRNA_101372	-1.2334	7.188922	-5.30224	0.001908	0.016742	-1.2199

hsa_circRNA_101712	-1.23449	7.996127	-3.54557	0.012425	0.05522	-3.27893
hsa_circRNA_101850	-1.23566	10.10628	-5.92647	0.001082	0.011995	-0.59499
hsa_circRNA_102702	-1.24549	7.215583	-3.32966	0.016149	0.064517	-3.56349
hsa_circRNA_100257	-1.24993	8.55401	-5.15211	0.002202	0.0179	-1.37808
hsa_circRNA_100713	-1.26387	6.706161	-2.61831	0.040199	0.121992	-4.53897
hsa_circRNA_101246	-1.26387	7.68	-4.98869	0.002582	0.020006	-1.55389
hsa_circRNA_102587	-1.27164	12.40142	-5.70111	0.001321	0.013528	-0.81467
hsa_circRNA_101555	-1.2824	10.6623	-5.39841	0.001743	0.015813	-1.12023
hsa_circRNA_103710	-1.28478	6.752868	-3.45445	0.013867	0.059511	-3.39825
hsa_circRNA_100587	-1.28564	11.19527	-7.22982	0.000379	0.007434	0.557329
hsa_circRNA_102613	-1.28908	6.715979	-3.67988	0.010594	0.049734	-3.10523
hsa_circRNA_001594	-1.29719	11.41145	-7.57099	0.000295	0.006539	0.829092
hsa_circRNA_100044	-1.2994	7.759571	-2.61006	0.040643	0.122912	-4.55052
hsa_circRNA_103998	-1.32555	7.800555	-5.02133	0.002501	0.01946	-1.51848
hsa_circRNA_000679	-1.32822	8.092713	-6.73782	0.000552	0.008688	0.144648
hsa_circRNA_104833	-1.33186	9.825332	-5.43572	0.001683	0.015685	-1.08191
hsa_circRNA_101924	-1.33196	9.781552	-6.19483	0.00086	0.010797	-0.34171
hsa_circRNA_102355	-1.35055	8.857688	-4.94943	0.002684	0.020427	-1.5967
hsa_circRNA_102885	-1.35612	7.561602	-4.70595	0.003431	0.023932	-1.86721
hsa_circRNA_100533	-1.35903	9.637213	-5.82488	0.001183	0.012553	-0.69321
hsa_circRNA_100157	-1.36245	7.523738	-2.55184	0.043926	0.128997	-4.63216
hsa_circRNA_100873	-1.37582	6.811903	-2.50286	0.046905	0.133763	-4.70092
hsa_circRNA_101175	-1.37761	7.493963	-3.10807	0.021289	0.078151	-3.8618
hsa_circRNA_102456	-1.38155	8.513366	-7.20113	0.000387	0.007434	0.53395
hsa_circRNA_104650	-1.41053	10.3614	-8.08987	0.000205	0.005369	1.221514
hsa_circRNA_100356	-1.44688	8.641862	-5.20572	0.002091	0.0175	-1.32124
hsa_circRNA_000764	-1.44881	11.58459	-7.74835	0.00026	0.006072	0.965975
hsa_circRNA_102645	-1.45061	8.554544	-4.88861	0.002852	0.020981	-1.66346
hsa_circRNA_103356	-1.45101	7.464269	-2.49523	0.047388	0.134577	-4.71163
hsa_circRNA_101906	-1.45467	12.36357	-6.62739	0.000603	0.009099	0.04845
hsa_circRNA_100657	-1.47142	7.155229	-4.35993	0.004931	0.030149	-2.26687
hsa_circRNA_103510	-1.47759	8.665042	-5.90532	0.001103	0.012066	-0.61533
hsa_circRNA_100192	-1.48896	11.40364	-7.8069	0.00025	0.005987	1.010524
hsa_circRNA_102546	-1.50476	9.648253	-7.90469	0.000233	0.005672	1.084243
hsa_circRNA_103526	-1.50737	6.792178	-3.46574	0.013678	0.058992	-3.3834
hsa_circRNA_000554	-1.52145	7.99022	-3.3476	0.015796	0.063439	-3.53959
hsa_circRNA_101902	-1.52746	8.680298	-5.59609	0.001453	0.014535	-0.9193
hsa_circRNA_105036	-1.52812	7.34418	-3.50989	0.012969	0.056906	-3.32552
hsa_circRNA_100815	-1.5373	9.337978	-6.22005	0.000842	0.010759	-0.31836
hsa_circRNA_001038	-1.53999	11.59671	-8.58002	0.000149	0.004409	1.570788
hsa_circRNA_104572	-1.55134	7.613636	-3.44236	0.014071	0.059864	-3.41416
hsa_circRNA_001059	-1.55489	10.40868	-8.12312	0.000201	0.005329	1.245844
hsa_circRNA_101798	-1.56641	11.77861	-7.47252	0.000317	0.00674	0.751813

hsa_circRNA_104866	-1.59127	7.881401	-4.36933	0.004882	0.030149	-2.25577
hsa_circRNA_100168	-1.59324	8.441172	-7.36128	0.000344	0.007026	0.663389
hsa_circRNA_104227	-1.59956	7.402371	-4.44648	0.004496	0.028733	-2.16521
hsa_circRNA_103655	-1.60318	9.447569	-6.12395	0.000913	0.011103	-0.40774
hsa_circRNA_104807	-1.62285	6.748783	-5.53903	0.00153	0.015036	-0.97676
hsa_circRNA_001396	-1.63747	8.193598	-6.44079	0.000701	0.009633	-0.11719
hsa_circRNA_100051	-1.64425	7.126014	-3.99982	0.007329	0.03927	-2.7021
hsa_circRNA_104285	-1.6451	7.118732	-4.56747	0.003959	0.026512	-2.02499
hsa_circRNA_104220	-1.6536	7.572731	-5.49245	0.001597	0.015452	-1.02398
hsa_circRNA_102562	-1.66774	12.99133	-9.06275	0.00011	0.003571	1.89611
hsa_circRNA_100498	-1.67707	9.573647	-6.63369	0.0006	0.009099	0.053973
hsa_circRNA_102014	-1.68845	6.487523	-3.02876	0.023545	0.083624	-3.96996
hsa_circRNA_100323	-1.69066	7.031167	-5.89168	0.001116	0.012135	-0.62847
hsa_circRNA_102777	-1.70248	6.177901	-3.40029	0.01481	0.061306	-3.4697
hsa_circRNA_001379	-1.72081	9.542568	-8.58061	0.000149	0.004409	1.5712
hsa_circRNA_100478	-1.72758	6.587262	-4.56206	0.003981	0.026512	-2.03122
hsa_circRNA_103740	-1.73073	8.275979	-6.70737	0.000566	0.008706	0.118254
hsa_circRNA_100272	-1.74589	7.866474	-6.752	0.000546	0.008688	0.156897
hsa_circRNA_100084	-1.79253	8.849438	-8.52848	0.000154	0.004409	1.534984
hsa_circRNA_102644	-1.79712	9.865538	-9.60674	7.92E-05	0.003081	2.242298
hsa_circRNA_101541	-1.81001	7.628312	-6.42948	0.000708	0.009633	-0.12737
hsa_circRNA_103339	-1.82173	8.924236	-7.64894	0.000279	0.006345	0.88961
hsa_circRNA_103951	-1.82243	10.44916	-7.08292	0.000423	0.007793	0.436754
hsa_circRNA_100640	-1.83014	8.74891	-7.09718	0.000418	0.007791	0.448557
hsa_circRNA_104764	-1.84497	6.482009	-3.87075	0.008487	0.043582	-2.86288
hsa_circRNA_101539	-1.85843	8.097173	-9.80892	7.04E-05	0.002937	2.365812
hsa_circRNA_104625	-1.87085	9.628834	-9.15729	0.000103	0.003571	1.957775
hsa_circRNA_000552	-1.87089	7.095601	-2.536	0.044866	0.130236	-4.65438
hsa_circRNA_103384	-1.90407	8.536246	-6.40704	0.000721	0.009633	-0.14759
hsa_circRNA_103265	-1.92371	7.118506	-3.13624	0.020546	0.076708	-3.82355
hsa_circRNA_101826	-1.9279	7.052882	-4.11783	0.006423	0.036277	-2.5573
hsa_circRNA_102213	-1.92922	9.214334	-3.6381	0.01113	0.051272	-3.15898
hsa_circRNA_102520	-1.94644	6.925176	-4.11189	0.006465	0.0364	-2.56453
hsa_circRNA_100476	-1.95658	7.300972	-3.07122	0.022306	0.080867	-3.91196
hsa_circRNA_103039	-1.96592	7.92087	-6.95806	0.000465	0.008144	0.332524
hsa_circRNA_103899	-2.03658	7.168985	-6.23409	0.000832	0.010711	-0.30539
hsa_circRNA_101592	-2.03998	8.304161	-5.93016	0.001079	0.011995	-0.59144
hsa_circRNA_101901	-2.04377	9.04945	-6.10635	0.000927	0.011124	-0.42424
hsa_circRNA_104169	-2.07445	9.036487	-5.35923	0.001808	0.016152	-1.16069
hsa_circRNA_100655	-2.079	8.095546	-11.6632	2.64E-05	0.001851	3.38453
hsa_circRNA_101272	-2.10244	6.451917	-2.63654	0.039238	0.119695	-4.51345
hsa_circRNA_102570	-2.10929	9.246963	-11.7162	2.58E-05	0.001851	3.410923
hsa_circRNA_101425	-2.16265	5.850865	-3.64928	0.010984	0.050756	-3.14457

hsa_circRNA_104595	-2.1684	11.28862	-7.54688	0.0003	0.00657	0.810253
hsa_circRNA_104701	-2.19739	5.996749	-3.59448	0.011721	0.052759	-3.21538
hsa_circRNA_104527	-2.20141	7.181895	-6.70473	0.000567	0.008706	0.115965
hsa_circRNA_104270	-2.21582	9.21155	-8.91834	0.00012	0.003749	1.800639
hsa_circRNA_102417	-2.22991	9.069491	-7.03684	0.000438	0.007987	0.398475
hsa_circRNA_101170	-2.23862	6.50314	-2.46095	0.049624	0.139473	-4.7598
hsa_circRNA_100600	-2.37566	6.004984	-2.82612	0.030576	0.101016	-4.24921
hsa_circRNA_105030	-2.37579	5.909369	-3.17007	0.019691	0.074712	-3.77773
hsa_circRNA_400087	-2.388	10.42334	-11.2015	3.33E-05	0.002157	3.148622
hsa_circRNA_101871	-2.39707	6.800583	-3.23267	0.018209	0.070227	-3.6933
hsa_circRNA_000585	-2.39917	7.664507	-10.6458	4.44E-05	0.002361	2.849716
hsa_circRNA_002143	-2.47386	8.533508	-8.34929	0.000173	0.004819	1.408852
hsa_circRNA_100970	-2.61177	10.12782	-9.38612	9.02E-05	0.003359	2.104394
hsa_circRNA_101318	-2.65134	5.77202	-3.63608	0.011156	0.051272	-3.16158
hsa_circRNA_000094	-2.66547	7.484044	-3.68329	0.010552	0.049668	-3.10084
hsa_circRNA_103050	-2.74207	5.788513	-2.55734	0.043603	0.128535	-4.62443
hsa_circRNA_100327	-2.93303	6.852084	-2.89468	0.027971	0.0951	-4.1543
hsa_circRNA_100389	-2.93921	6.383972	-2.58139	0.042226	0.125602	-4.59071
hsa_circRNA_102720	-3.16338	9.194669	-14.2727	8.30E-06	0.001038	4.538422
hsa_circRNA_103312	-3.16357	5.628597	-2.99751	0.024504	0.086329	-4.01276
hsa_circRNA_101055	-3.16414	7.282061	-6.73309	0.000554	0.008688	0.140554
hsa_circRNA_101128	-3.18574	9.213483	-7.18802	0.000391	0.007434	0.523244
hsa_circRNA_100999	-3.18728	6.27751	-3.09812	0.021559	0.078721	-3.87533
hsa_circRNA_100001	-3.28398	11.24796	-15.0596	6.09E-06	0.000888	4.835804
hsa_circRNA_100835	-3.30461	5.975784	-3.72527	0.010045	0.04805	-3.04711
hsa_circRNA_002117	-3.36742	6.729583	-3.35579	0.015638	0.063248	-3.52871
hsa_circRNA_103309	-3.373	9.944317	-11.951	2.30E-05	0.001751	3.526266
hsa_circRNA_104698	-3.577	5.397056	-4.43164	0.004568	0.02895	-2.18256
hsa_circRNA_103503	-3.63113	5.869535	-5.19252	0.002118	0.017575	-1.3352
hsa_circRNA_101478	-3.6541	9.024019	-11.9718	2.28E-05	0.001751	3.536335
hsa_circRNA_102830	-3.67326	5.457791	-9.67511	7.61E-05	0.003028	2.284369
hsa_circRNA_102328	-3.67605	5.610733	-3.59716	0.011683	0.052726	-3.2119
hsa_circRNA_102228	-3.68003	7.082197	-3.30053	0.016739	0.066068	-3.60234
hsa_circRNA_100611	-3.68697	5.790624	-6.87325	0.000497	0.008442	0.26079
hsa_circRNA_100228	-3.71244	5.280078	-9.20992	0.0001	0.003571	1.991825
hsa_circRNA_100568	-3.76127	5.905435	-6.39457	0.000728	0.009658	-0.15885
hsa_circRNA_102049	-3.77175	11.48629	-10.401	5.06E-05	0.002463	2.712561
hsa_circRNA_103230	-3.89122	5.60305	-5.44065	0.001676	0.015685	-1.07685
hsa_circRNA_102245	-3.99436	6.229643	-5.41669	0.001713	0.015685	-1.10143
hsa_circRNA_100079	-3.99564	5.695494	-6.30721	0.000783	0.010151	-0.23824
hsa_circRNA_103559	-4.0028	8.387412	-5.8184	0.00119	0.012553	-0.69952
hsa_circRNA_102711	-4.0247	5.668945	-4.35474	0.004959	0.030149	-2.273
hsa_circRNA_102051	-4.04827	9.52041	-12.3612	1.90E-05	0.001581	3.721504

hsa_circRNA_104948	-4.07577	5.426717	-7.98733	0.00022	0.005591	1.145872
hsa_circRNA_102334	-4.1205	6.41315	-4.62538	0.003727	0.025595	-1.95867
hsa_circRNA_104022	-4.18985	6.321461	-5.12147	0.002268	0.018217	-1.41076
hsa_circRNA_103560	-4.28706	9.604164	-13.3862	1.20E-05	0.001305	4.177432
hsa_circRNA_102010	-4.29386	5.963205	-3.9809	0.007487	0.039726	-2.72551
hsa_circRNA_103572	-4.40748	5.296428	-9.40851	8.90E-05	0.003359	2.118539
hsa_circRNA_002172	-4.43314	6.920996	-4.22495	0.005708	0.033314	-2.42769
hsa_circRNA_102400	-4.53199	6.229522	-3.92021	0.008021	0.041848	-2.80097
hsa_circRNA_100790	-4.63258	6.105117	-5.62192	0.001419	0.014362	-0.89343
hsa_circRNA_104689	-4.63864	5.10938	-12.6452	1.67E-05	0.001581	3.852269
hsa_circRNA_103642	-4.64262	5.386064	-9.96674	6.44E-05	0.002861	2.460374
hsa_circRNA_102737	-4.7646	4.876145	-21.3052	8.08E-07	0.000472	6.618553
hsa_circRNA_000166	-4.81538	7.484159	-5.42593	0.001699	0.015685	-1.09194
hsa_circRNA_100653	-4.82363	7.33824	-4.42913	0.00458	0.02895	-2.1855
hsa_circRNA_102829	-4.87849	5.663758	-7.62698	0.000283	0.006363	0.872621
hsa_circRNA_104575	-4.91963	7.116115	-9.94068	6.54E-05	0.002861	2.444866
hsa_circRNA_104645	-5.03405	5.010874	-15.8741	4.49E-06	0.000786	5.122897
hsa_circRNA_102619	-5.06291	6.946808	-6.059	0.000965	0.011496	-0.46879
hsa_circRNA_001676	-5.17355	10.49776	-9.11949	0.000106	0.003571	1.933196
hsa_circRNA_002144	-5.23506	6.719414	-6.87921	0.000494	0.008442	0.265856
hsa_circRNA_100656	-5.28526	6.457318	-7.33926	0.000349	0.007026	0.645743
hsa_circRNA_101119	-5.28647	5.137083	-16.4004	3.71E-06	0.000786	5.298139
hsa_circRNA_104418	-5.30139	5.144543	-10.6414	4.45E-05	0.002361	2.84727
hsa_circRNA_104417	-5.32558	5.727543	-11.0707	3.56E-05	0.002211	3.079753
hsa_circRNA_102719	-5.42154	5.204615	-24.334	3.71E-07	0.000325	7.220418
hsa_circRNA_102672	-5.5409	5.642697	-16.0414	4.22E-06	0.000786	5.179441
hsa_circRNA_104084	-5.57175	9.193132	-29.767	1.14E-07	0.000199	8.028536
hsa_circRNA_104419	-5.58596	6.185534	-7.78104	0.000254	0.006014	0.990887
hsa_circRNA_103109	-5.69509	6.275104	-7.32259	0.000353	0.007032	0.632353
hsa_circRNA_102928	-6.25992	6.675856	-17.8435	2.27E-06	0.000693	5.741184
hsa_circRNA_400029	-6.63571	5.811702	-13.2612	1.27E-05	0.001305	4.124138
hsa_circRNA_103111	-6.83191	6.9527	-6.72972	0.000556	0.008688	0.137637
hsa_circRNA_001826	-7.03118	6.009439	-17.2738	2.75E-06	0.000693	5.572475
hsa_circRNA_103110	-7.14727	6.944374	-6.97129	0.00046	0.008143	0.343647

Table S4. The potential circTFRC-binding proteins predicted by ENCORI database

RBP	geneID	geneName	geneType	clusterNum	clipExpNum	clipIDnum
ELAVL1	NM_003234	TFRC	circRNA	34	28	188
TARDBP	NM_003234	TFRC	circRNA	21	28	161
HNRNPC	NM_003234	TFRC	circRNA	34	22	163
RNPS1	NM_003234	TFRC	circRNA	31	16	291
U2AF2	NM_003234	TFRC	circRNA	29	15	235
FUS	NM_003234	TFRC	circRNA	11	14	52
RBFOX2	NM_003234	TFRC	circRNA	5	14	20
PTBP1	NM_003234	TFRC	circRNA	26	13	56
U2AF1	NM_003234	TFRC	circRNA	23	11	82
UPF1	NM_003234	TFRC	circRNA	15	11	96
G3BP1	NM_003234	TFRC	circRNA	10	9	47
ALYREF	NM_003234	TFRC	circRNA	23	8	71
ELAVL3	NM_003234	TFRC	circRNA	7	8	15
LIN28B	NM_003234	TFRC	circRNA	25	8	80
RBMX	NM_003234	TFRC	circRNA	22	8	40
IGF2BP1	NM_003234	TFRC	circRNA	12	7	35
IGF2BP3	NM_003234	TFRC	circRNA	9	7	26
YTHDC1	NM_003234	TFRC	circRNA	6	7	13
ACIN1	NM_003234	TFRC	circRNA	18	6	68
DDX3X	NM_003234	TFRC	circRNA	17	6	35
NXF1	NM_003234	TFRC	circRNA	10	6	14
PCBP2	NM_003234	TFRC	circRNA	9	6	31
SCAF4	NM_003234	TFRC	circRNA	21	6	57
SCAF8	NM_003234	TFRC	circRNA	18	6	53
YBX1	NM_003234	TFRC	circRNA	12	6	44
YTHDF1	NM_003234	TFRC	circRNA	13	6	30
YTHDF2	NM_003234	TFRC	circRNA	9	6	16
HNRNPA1	NM_003234	TFRC	circRNA	21	5	34
IGF2BP2	NM_003234	TFRC	circRNA	19	5	38
KHDRBS2	NM_003234	TFRC	circRNA	3	5	9
NUDT21	NM_003234	TFRC	circRNA	11	5	19
RC3H1	NM_003234	TFRC	circRNA	4	5	12
SLBP	NM_003234	TFRC	circRNA	10	5	12
SRSF1	NM_003234	TFRC	circRNA	19	5	38
TIA1	NM_003234	TFRC	circRNA	6	5	14
TIAL1	NM_003234	TFRC	circRNA	4	5	9
CHTOP	NM_003234	TFRC	circRNA	6	4	24
CPSF6	NM_003234	TFRC	circRNA	10	4	17
CSTF2	NM_003234	TFRC	circRNA	15	4	25
DHX9	NM_003234	TFRC	circRNA	5	4	20
EWSR1	NM_003234	TFRC	circRNA	31	4	56

FAM120A	NM_003234	TFRC	circRNA	4	4	12
GRSF1	NM_003234	TFRC	circRNA	1	4	8
HNRNPL	NM_003234	TFRC	circRNA	7	4	11
ILF3	NM_003234	TFRC	circRNA	9	4	23
LARP1	NM_003234	TFRC	circRNA	3	4	10
PRPF8	NM_003234	TFRC	circRNA	23	4	63
RBM7	NM_003234	TFRC	circRNA	11	4	27
SND1	NM_003234	TFRC	circRNA	19	4	66
CSTF2T	NM_003234	TFRC	circRNA	10	3	12
CTCF	NM_003234	TFRC	circRNA	18	3	48
DDX5	NM_003234	TFRC	circRNA	4	3	11
DDX54	NM_003234	TFRC	circRNA	6	3	7
DGCR8	NM_003234	TFRC	circRNA	3	3	7
DHX36	NM_003234	TFRC	circRNA	9	3	46
EIF4A3	NM_003234	TFRC	circRNA	26	3	48
EXOSC10	NM_003234	TFRC	circRNA	4	3	7
HNRNPA2B1	NM_003234	TFRC	circRNA	16	3	39
HNRNPM	NM_003234	TFRC	circRNA	2	3	3
HNRNPU	NM_003234	TFRC	circRNA	22	3	35
METTL3	NM_003234	TFRC	circRNA	6	3	8
MOV10	NM_003234	TFRC	circRNA	3	3	31
MSI1	NM_003234	TFRC	circRNA	6	3	15
MSI2	NM_003234	TFRC	circRNA	15	3	25
MTDH	NM_003234	TFRC	circRNA	20	3	88
NOLC1	NM_003234	TFRC	circRNA	5	3	7
PUM1	NM_003234	TFRC	circRNA	4	3	4
RBM20	NM_003234	TFRC	circRNA	2	3	5
RPS3	NM_003234	TFRC	circRNA	6	3	7
SNRPA	NM_003234	TFRC	circRNA	5	3	7
SP1	NM_003234	TFRC	circRNA	3	3	4
TAF15	NM_003234	TFRC	circRNA	4	3	5
TRIM25	NM_003234	TFRC	circRNA	2	3	5
ZNF800	NM_003234	TFRC	circRNA	4	3	4
AKAP1	NM_003234	TFRC	circRNA	2	2	3
AQR	NM_003234	TFRC	circRNA	15	2	63
BCLAF1	NM_003234	TFRC	circRNA	10	2	16
CDK1	NM_003234	TFRC	circRNA	1	2	2
CENPC	NM_003234	TFRC	circRNA	1	2	2
CPSF4	NM_003234	TFRC	circRNA	2	2	2
DAP3	NM_003234	TFRC	circRNA	8	2	13
DDX42	NM_003234	TFRC	circRNA	5	2	6
DROSHA	NM_003234	TFRC	circRNA	2	2	2
FIP1L1	NM_003234	TFRC	circRNA	7	2	9

FMR1	NM_003234	TFRC	circRNA	8	2	12
GRWD1	NM_003234	TFRC	circRNA	15	2	32
HDLBP	NM_003234	TFRC	circRNA	19	2	63
KHDRBS1	NM_003234	TFRC	circRNA	8	2	11
LIN28A	NM_003234	TFRC	circRNA	13	2	14
MARF1	NM_003234	TFRC	circRNA	3	2	7
METTL1	NM_003234	TFRC	circRNA	1	2	3
NIPBL	NM_003234	TFRC	circRNA	2	2	2
NUDT16L1	NM_003234	TFRC	circRNA	8	2	14
PABPC4	NM_003234	TFRC	circRNA	3	2	4
QKI	NM_003234	TFRC	circRNA	3	2	5
RBFOX1	NM_003234	TFRC	circRNA	6	2	54
RBM10	NM_003234	TFRC	circRNA	12	2	20
RBM12	NM_003234	TFRC	circRNA	3	2	3
RBM15	NM_003234	TFRC	circRNA	8	2	11
RBM15B	NM_003234	TFRC	circRNA	3	2	5
RBM22	NM_003234	TFRC	circRNA	12	2	20
RBM4	NM_003234	TFRC	circRNA	4	2	5
RBM47	NM_003234	TFRC	circRNA	16	2	20
SFPQ	NM_003234	TFRC	circRNA	4	2	5
SOX2	NM_003234	TFRC	circRNA	11	2	14
SRRM4	NM_003234	TFRC	circRNA	4	2	4
SRSF2	NM_003234	TFRC	circRNA	2	2	3
SRSF3	NM_003234	TFRC	circRNA	8	2	11
SRSF7	NM_003234	TFRC	circRNA	15	2	19
SRSF9	NM_003234	TFRC	circRNA	2	2	2
TFIP11	NM_003234	TFRC	circRNA	5	2	5
TRA2B	NM_003234	TFRC	circRNA	1	2	2
WDR4	NM_003234	TFRC	circRNA	4	2	9
XRN2	NM_003234	TFRC	circRNA	6	2	8
YBX3	NM_003234	TFRC	circRNA	5	2	7
ZCCHC14	NM_003234	TFRC	circRNA	9	2	11
AATF	NM_003234	TFRC	circRNA	1	1	1
ATXN2	NM_003234	TFRC	circRNA	3	1	5
CPSF1	NM_003234	TFRC	circRNA	4	1	4
CPSF7	NM_003234	TFRC	circRNA	17	1	18
DDX21	NM_003234	TFRC	circRNA	1	1	1
EIF3A	NM_003234	TFRC	circRNA	3	1	5
FTO	NM_003234	TFRC	circRNA	12	1	13
FUBP3	NM_003234	TFRC	circRNA	1	1	1
FXR1	NM_003234	TFRC	circRNA	8	1	9
FXR2	NM_003234	TFRC	circRNA	1	1	1
G3BP2	NM_003234	TFRC	circRNA	1	1	1

GEMIN5	NM_003234	TFRC	circRNA	1	1	2
GNL3	NM_003234	TFRC	circRNA	6	1	6
GTF2F1	NM_003234	TFRC	circRNA	1	1	1
HLTF	NM_003234	TFRC	circRNA	1	1	1
HNRNPD	NM_003234	TFRC	circRNA	1	1	1
KHSRP	NM_003234	TFRC	circRNA	2	1	2
LARP4B	NM_003234	TFRC	circRNA	1	1	2
MBNL1	NM_003234	TFRC	circRNA	2	1	2
MTA1	NM_003234	TFRC	circRNA	5	1	5
NAT10	NM_003234	TFRC	circRNA	5	1	6
NONO	NM_003234	TFRC	circRNA	2	1	2
OAS1	NM_003234	TFRC	circRNA	1	1	1
POLR2A	NM_003234	TFRC	circRNA	3	1	3
PRPF4	NM_003234	TFRC	circRNA	1	1	1
RBM27	NM_003234	TFRC	circRNA	1	1	1
RBPMS	NM_003234	TFRC	circRNA	1	1	1
RNF10	NM_003234	TFRC	circRNA	1	1	1
RO60	NM_003234	TFRC	circRNA	1	1	1
RTCB	NM_003234	TFRC	circRNA	2	1	2
SERBP1	NM_003234	TFRC	circRNA	3	1	3
SF3A3	NM_003234	TFRC	circRNA	2	1	2
SF3B1	NM_003234	TFRC	circRNA	3	1	3
SF3B4	NM_003234	TFRC	circRNA	2	1	2
SLTM	NM_003234	TFRC	circRNA	1	1	1
SMNDC1	NM_003234	TFRC	circRNA	1	1	1
SRSF6	NM_003234	TFRC	circRNA	1	1	1
SSB	NM_003234	TFRC	circRNA	1	1	1
TNRC6A	NM_003234	TFRC	circRNA	1	1	1
TOP3B	NM_003234	TFRC	circRNA	6	1	6
TRA2A	NM_003234	TFRC	circRNA	9	1	9
UTP3	NM_003234	TFRC	circRNA	1	1	1
WDR33	NM_003234	TFRC	circRNA	1	1	1
YTHDF3	NM_003234	TFRC	circRNA	3	1	5
ZC3H11A	NM_003234	TFRC	circRNA	1	1	1
ZC3H7B	NM_003234	TFRC	circRNA	7	1	18

Table S5. The potential circTFRC-binding proteins predicted by two RBPDB database

Score	Relative Score	RBP Name	Start	End	Matching sequence
13.856096	91%	SNRPA	973	982	GGGAAUGCUG
11.070935	100%	A2BP1	222	227	UGCAUG
10.97654	89%	SFRS2	1391	1399	AGGAGAGGA
9.4281438	100%	ybx2-a	154	159	AACAUC
9.3741518	100%	ybx2-a	154	159	AACAUC
8.9484945	100%	NONO	1380	1384	AGGGA
8.9484945	100%	NONO	1832	1836	AGGGA
8.9484945	100%	NONO	859	863	AGGGA
8.9484945	100%	NONO	395	399	AGGGA
8.883996	87%	SNRPA	1162	1168	UGGAGAU
8.7846348	100%	sap-49	1346	1351	GUGUGA
8.7178165	100%	PABPC1	1337	1341	AAAAA
8.7178165	100%	PABPC1	1774	1778	AAAAA
8.7178165	100%	PABPC1	1232	1236	AAAAA
8.7178165	100%	PABPC1	1773	1777	AAAAA
8.7178165	100%	PABPC1	1534	1538	AAAAA
8.7178165	100%	PABPC1	144	148	AAAAA
8.6696024	100%	RBMY1A1	1012	1016	CUCAA
8.6696024	100%	RBMY1A1	1642	1646	CUCAA
8.6696024	100%	RBMY1A1	1531	1535	CUCAA
8.6471013	100%	a2bp1	223	227	GCAUG
8.6272192	100%	RBMY1A1	1012	1016	CUCAA
8.6272192	100%	RBMY1A1	1642	1646	CUCAA
8.6272192	100%	RBMY1A1	1531	1535	CUCAA
7.6563361	88%	RBMY1A1	1208	1212	CACAA
7.6563361	88%	RBMY1A1	1223	1227	CACAA
7.6146688	90%	PABPC1	792	798	ACUAACC
7.6146688	90%	PABPC1	957	963	ACUAACA
7.5622424	86%	sap-49	1601	1606	GCGUGA
7.5622424	86%	sap-49	854	859	UUGUGA
7.5200189	88%	PTBP1	1934	1948	AGAACUUUCAUUCUU
7.3693752	100%	FUS	376	379	GGUG
7.3693752	100%	FUS	1095	1098	GGUG
7.3693752	100%	FUS	1887	1890	GGUG
7.3693752	100%	FUS	250	253	GGUG
7.3693752	100%	FUS	1652	1655	GGUG
7.3693752	100%	FUS	1691	1694	GGUG
7.3693752	100%	FUS	1237	1240	GGUG
7.3693752	100%	FUS	1971	1974	GGUG
7.2294196	100%	Pum2	1555	1558	UGUA

7.2294196	100%	Pum2	79	82	UGUA
7.2294196	100%	Pum2	917	920	UGUA
7.2294196	100%	Pum2	201	204	UGUA
7.2294196	100%	Pum2	1320	1323	UGUA
7.2294196	100%	Pum2	180	183	UGUA
7.2294196	100%	Pum2	1239	1242	UGUA
7.2294196	100%	Pum2	1178	1181	UGUA
7.2294196	100%	Pum2	497	500	UGUA
7.2294196	100%	Pum2	252	255	UGUA
7.0865209	100%	SFRS9	563	567	AGGAC
7.0865209	100%	SFRS9	668	672	AGGAC
7.0865209	100%	SFRS9	1396	1400	AGGAC
6.9329262	94%	ACO1	1375	1380	CAGUGA
6.6325519	93%	SFRS9	1384	1388	AGGAG
6.6325519	93%	SFRS9	42	46	AGGAG
6.6325519	93%	SFRS9	1391	1395	AGGAG
6.6279899	100%	MBNL1	845	848	UGCU
6.6279899	100%	MBNL1	978	981	UGCU
6.6279899	100%	MBNL1	354	357	UGCU
6.6279899	100%	MBNL1	1254	1257	UGCU
6.6279899	100%	MBNL1	1268	1271	UGCU
6.6279899	100%	MBNL1	12	15	UGCU
6.6279899	100%	MBNL1	945	948	UGCU
6.6279899	100%	MBNL1	924	927	UGCU
6.6279899	100%	MBNL1	1955	1958	UGCU
6.6279899	100%	MBNL1	1196	1199	UGCU
6.6279899	100%	MBNL1	1175	1178	UGCU
6.6279899	100%	MBNL1	131	134	UGCU
6.6279899	100%	MBNL1	1757	1760	UGCU
6.6279899	100%	MBNL1	609	612	UGCU
6.6279899	100%	MBNL1	1492	1495	UGCU
6.6279899	100%	MBNL1	612	615	UGCU
6.6279899	100%	MBNL1	1865	1868	UGCU
6.4668404	100%	EIF4B	1245	1248	GGAA
6.4668404	100%	EIF4B	62	65	GGAA
6.4668404	100%	EIF4B	891	894	GGAA
6.4668404	100%	EIF4B	1834	1837	GGAA
6.4668404	100%	EIF4B	974	977	GGAA
6.4668404	100%	EIF4B	637	640	GGAA
6.4668404	100%	EIF4B	32	35	GGAA
6.4668404	100%	EIF4B	714	717	GGAA
6.4668404	100%	EIF4B	1362	1365	GGAA

6.4668404	100%	EIF4B	39	42	GGAA
6.33985	100%	KHSRP	1752	1755	GUCC
6.33985	100%	KHSRP	408	411	GUCC
6.33985	100%	KHSRP	2055	2058	GUCC
6.33985	100%	KHSRP	1512	1515	GUCC
6.33985	100%	KHSRP	50	53	GUCC
6.2357076	100%	YTHDC1	975	980	GAAUGC
6.1783203	93%	MBNL1	503	506	CGCU
6.1783203	93%	MBNL1	771	774	CGCU
6.1783203	93%	MBNL1	1416	1419	CGCU
6.1783203	93%	MBNL1	1640	1643	CGCU
5.898417	92%	QKI	150	159	UCUUAACAUC
5.8302315	82%	SFRS9	1464	1468	AGCAC
5.8302315	82%	SFRS9	1409	1413	AGCAC
5.7148754	91%	YTHDC1	351	356	GAGUGC
5.6537433	88%	Vts1	772	778	GCUGGUC
5.5294388	88%	YTHDC1	1697	1702	GAAUCC
5.5294388	88%	YTHDC1	638	643	GAAUCC
5.2761516	82%	QKI	956	965	GACUAACAAC
5.2682554	100%	RBMX	464	467	CCAG
5.2682554	100%	RBMX	213	216	CCAG
5.2682554	100%	RBMX	2057	2060	CCAG
5.2682554	100%	RBMX	643	646	CCAG
5.2682554	100%	RBMX	282	285	CCAG
5.2682554	100%	RBMX	1389	1392	CCAG
5.2682554	100%	RBMX	323	326	CCAG
5.2682554	100%	RBMX	1907	1910	CCAG
5.2682554	100%	RBMX	953	956	CCAG
5.2682554	100%	RBMX	1374	1377	CCAG
5.2682554	100%	RBMX	485	488	CCAG
5.2682554	100%	RBMX	187	190	CCAG
5.2682554	100%	RBMX	344	347	CCAG
5.2682554	100%	RBMX	578	581	CCAG
5.2682554	100%	RBMX	5	8	CCAG
5.2566459	81%	EIF4B	1397	1400	GGAC
5.2566459	81%	EIF4B	1460	1463	GGAC
5.2566459	81%	EIF4B	564	567	GGAC
5.2566459	81%	EIF4B	819	822	GGAC
5.2566459	81%	EIF4B	1904	1907	GGAC
5.2566459	81%	EIF4B	1261	1264	GGAC
5.2566459	81%	EIF4B	702	705	GGAC
5.2566459	81%	EIF4B	1950	1953	GGAC

5.2566459	81%	EIF4B	1966	1969	GGAC
5.2566459	81%	EIF4B	669	672	GGAC
5.1999416	81%	Vts1	355	361	GCUGGAG
5.1779664	83%	YTHDC1	534	539	GCAUCC
4.9986159	94%	RBMX	2022	2025	CCAU
4.9986159	94%	RBMX	1483	1486	CCAU
4.9986159	94%	RBMX	1921	1924	CCAU
4.9986159	94%	RBMX	1754	1757	CCAU
4.9986159	94%	RBMX	1104	1107	CCAU
4.9986159	94%	RBMX	798	801	CCAU
4.9986159	94%	RBMX	698	701	CCAU
4.939432	96%	SFRS13A	1211	1217	AAAGGCC
4.751282	92%	SFRS13A	1323	1329	AAAGGGG
4.6667232	88%	RBMX	2019	2022	CCAC
4.6667232	88%	RBMX	380	383	CCAC
4.6667232	88%	RBMX	695	698	CCAC
4.6667232	88%	RBMX	490	493	CCAC
4.6202877	100%	SFRS1	1786	1789	AGGA
4.6202877	100%	SFRS1	1384	1387	AGGA
4.6202877	100%	SFRS1	713	716	AGGA
4.6202877	100%	SFRS1	1361	1364	AGGA
4.6202877	100%	SFRS1	42	45	AGGA
4.6202877	100%	SFRS1	563	566	AGGA
4.6202877	100%	SFRS1	727	730	AGGA
4.6202877	100%	SFRS1	82	85	AGGA
4.6202877	100%	SFRS1	890	893	AGGA
4.6202877	100%	SFRS1	1391	1394	AGGA
4.6202877	100%	SFRS1	668	671	AGGA
4.6202877	100%	SFRS1	1396	1399	AGGA
4.6202877	100%	SFRS1	2036	2039	AGGA
4.5678334	89%	SFRS13A	420	426	AAAGGCU
4.5678334	89%	SFRS13A	172	178	AAAGGCU
4.5139002	88%	SFRS13A	1394	1400	AGAGGAC
4.5057134	88%	SFRS13A	888	894	AAAGGAA
4.5002791	88%	SFRS13A	136	142	AAAGAGA
4.4205509	95%	KHDRBS3	2043	2048	CCUAAU
4.4188893	94%	KHDRBS3	436	441	AUUAAU
4.4035906	100%	ELAVL1	27	30	GUUU
4.4035906	100%	ELAVL1	478	481	GUUU
4.4035906	100%	ELAVL1	905	908	GUUU
4.4035906	100%	ELAVL1	649	652	GUUU
4.4035906	100%	ELAVL1	2015	2018	GUUU

4.4035906	100%	ELAVL1	315	318	GUUU
4.4035906	100%	ELAVL1	659	662	GUUU
4.4035906	100%	ELAVL1	1091	1094	GUUU
4.4035906	100%	ELAVL1	1683	1686	GUUU
4.4027117	83%	RBMX	1117	1120	CCCG
4.3806082	85%	SFRS13A	1536	1542	AAAGAUG
4.3806082	85%	SFRS13A	1632	1638	AAAGACA
4.3806082	85%	SFRS13A	307	313	AAAGAUG
4.3576159	85%	SFRS13A	1234	1240	AAAGGUG
4.3168304	84%	SFRS13A	1776	1782	AAAGAUU
4.2938381	84%	SFRS13A	1853	1859	AAAGGUU
4.2774947	91%	KHDRBS3	885	890	CAUAAA
4.2658288	91%	KHDRBS3	1758	1763	GCUAAU
4.1881759	90%	SFRS1	1459	1462	UGGA
4.1881759	90%	SFRS1	1162	1165	UGGA
4.1881759	90%	SFRS1	701	704	UGGA
4.1881759	90%	SFRS1	1060	1063	UGGA
4.1881759	90%	SFRS1	818	821	UGGA
4.1881759	90%	SFRS1	1292	1295	UGGA
4.1881759	90%	SFRS1	366	369	UGGA
4.1881759	90%	SFRS1	1693	1696	UGGA
4.1881759	90%	SFRS1	1988	1991	UGGA
4.1881759	90%	SFRS1	162	165	UGGA
4.1881759	90%	SFRS1	1903	1906	UGGA
4.1881759	90%	SFRS1	61	64	UGGA
4.1881759	90%	SFRS1	636	639	UGGA
4.1881759	90%	SFRS1	234	237	UGGA
4.1881759	90%	SFRS1	1949	1952	UGGA
4.1881759	90%	SFRS1	38	41	UGGA
4.1881759	90%	SFRS1	930	933	UGGA
4.1881759	90%	SFRS1	357	360	UGGA
4.1881759	90%	SFRS1	1526	1529	UGGA
4.1881759	90%	SFRS1	443	446	UGGA
4.1881759	90%	SFRS1	1244	1247	UGGA
4.1881759	90%	SFRS1	1097	1100	UGGA
4.1881759	90%	SFRS1	349	352	UGGA
4.1881759	90%	SFRS1	1808	1811	UGGA
4.1423015	81%	SFRS13A	725	731	AGAGGAU
4.1421431	89%	KHDRBS3	1911	1916	ACUAAA
4.1421431	89%	KHDRBS3	1770	1775	ACUAAA
4.1227726	88%	KHDRBS3	445	450	GAUAAA
4.1227726	88%	KHDRBS3	141	146	GAUAAA

4.0923883	87%	KHDRBS3	169	174	AUUAAA
4.0923883	87%	KHDRBS3	787	792	AUUAAA
4.0918075	80%	SFRS13A	765	771	AGAGGUC
4.0442951	86%	KHDRBS3	1581	1586	UUUAAA
4.0442951	86%	KHDRBS3	1874	1879	CUUAAA
4.0442951	86%	KHDRBS3	303	308	CUUAAA
4.0442951	86%	KHDRBS3	417	422	UUUAAA
4.0207339	86%	KHDRBS3	1203	1208	AAUAAC
3.8372892	82%	KHDRBS3	792	797	ACUAAC
3.8372892	82%	KHDRBS3	957	962	ACUAAC
3.826364	86%	ELAVL1	1580	1583	AUUU
3.826364	86%	ELAVL1	1295	1298	AUUU
3.826364	86%	ELAVL1	1571	1574	AUUU
3.826364	86%	ELAVL1	996	999	AUUU
3.826364	86%	ELAVL1	686	689	AUUU
3.826364	86%	ELAVL1	852	855	AUUU
3.826364	86%	ELAVL1	968	971	AUUU
3.826364	86%	ELAVL1	1612	1615	AUUU
3.826364	86%	ELAVL1	1789	1792	AUUU
3.826364	86%	ELAVL1	416	419	AUUU
3.826364	86%	ELAVL1	1780	1783	AUUU
3.826364	86%	ELAVL1	1916	1919	AUUU
3.826364	86%	ELAVL1	552	555	AUUU
3.826364	86%	ELAVL1	1762	1765	AUUU
3.8261165	82%	KHDRBS3	1320	1325	UGUAAA
3.8179186	82%	KHDRBS3	1149	1154	GAUAAC
3.789196	81%	KHDRBS3	1083	1088	UCUAAC
3.7394411	80%	KHDRBS3	151	156	CUUAAC

Table S6. The ferroptosis-suppressor genes in the FerrDb V2 database

id	rcd	symbol	testin	confidence	uniprotac	pmid
1	Ferroptosis	SLC7A11	Human	Validated	Q9UPY5	22632970
2	Ferroptosis	GPX4	Human, mice	Validated	P36969	24439385
3	Ferroptosis	AKR1C1	Human	Validated	Q04828	24844246
4	Ferroptosis	AKR1C2	Human	Validated	P52895	24844246
5	Ferroptosis	AKR1C3	Human	Validated	P42330	24844246
6	Ferroptosis	GPX4	Human, mice	Validated	P36969	25402683
7	Ferroptosis	RB1	Human, mice	Validated	P06400	25444922
8	Ferroptosis	HSPB1	Human, mice	Validated	P04792	25728673
9	Ferroptosis	HSF1	Human, mice	Validated	Q00613	25728673
10	Ferroptosis	SLC7A11	Human, mice	Validated	Q9UPY5	25799988
11	Ferroptosis	GPX4	Mice	Validated	P36969	25824823
12	Ferroptosis	GCLC	Mice	Validated	P48506	26166707
13	Ferroptosis	SLC7A11	Human	Validated	Q9UPY5	26218928
14	Ferroptosis	NFE2L2	Human, mice	Validated	Q16236	26403645
15	Ferroptosis	SQSTM1	Human, mice	Validated	Q13501	26403645
16	Ferroptosis	NQO1	Human, mice	Validated	P15559	26403645
17	Ferroptosis	HMOX1	Human, mice	Validated	P09601	26403645
18	Ferroptosis	FTH1	Human, mice	Validated	P02794	26403645
19	Ferroptosis	MUC1	Human	Validated	P15941	26930718
20	Ferroptosis	SLC3A2	Mice	Validated	P08195	26945935
21	Ferroptosis	MT1G	Human, mice	Validated	P13640	27015352
22	Ferroptosis	NFE2L2	Human	Deduced	Q16236	27015352
23	Ferroptosis	SLC40A1	Human	Validated	Q9NP59	27441659
24	Ferroptosis	SLC7A11	Human	Validated	Q9UPY5	27441659
25	Ferroptosis	GPX4	Human	Validated	P36969	27441659
26	Ferroptosis	SLC7A11	Human, mice	Validated	Q9UPY5	27477897
27	Ferroptosis	CISD1	Human	Validated	Q9NZ45	27510639
28	Ferroptosis	SLC7A11	Human	Validated	Q9UPY5	27705786
29	Ferroptosis	FANCD2	Mice	Validated	Q9BXW9	27773819
30	Ferroptosis	GPX4	Human	Validated	P36969	27964880
31	Ferroptosis	NFE2L2	Mice	Validated	Q16236	28012440
32	Ferroptosis	FTMT	Mice, fly	Validated	Q8N4E7	28066232
33	Ferroptosis	HSPA5	Human, mice	Validated	P11021	28130223
34	Ferroptosis	ATF4	Human	Validated	P18848	28130223
35	Ferroptosis	SLC7A11	Mice	Validated	Q9UPY5	28195347
36	Ferroptosis	GPX4	Mice	Validated	P36969	28212525
37	Ferroptosis	GPX4	Human	Validated	P36969	28297659
38	Ferroptosis	HMOX1	Mice	Validated	P09601	28515173
39	Ferroptosis	ATF4	Human	Validated	P18848	28553953
40	Ferroptosis	NFE2L2	Human, rat	Validated	Q16236	28805788
41	Ferroptosis	TP53	Human, mice	Validated	P04637	28813679

42	Ferroptosis	SLC7A11	Human	Validated	Q9UPY5	28813679
43	Ferroptosis	HELLS	Human, mice	Validated	Q9NRZ9	28900510
44	Ferroptosis	SCD	Human	Validated	O00767	28900510
45	Ferroptosis	FADS2	Human	Validated	O95864	28900510
46	Ferroptosis	SRC	Human	Validated	P12931	28972104
47	Ferroptosis	STAT3	Human	Validated	P40763	28972104
48	Ferroptosis	NFE2L2	Human, mice	Validated	Q16236	28985506
49	Ferroptosis	PML	Human	Validated	P29590	29081404
50	Ferroptosis	MTOR	Mice	Validated	P42345	29127238
51	Ferroptosis	NFS1	Human	Validated	Q9Y697	29168506
52	Ferroptosis	TP63	Human	Validated	Q9H3D4	29212036
53	Ferroptosis	SLC7A11	Human	Validated	Q9UPY5	29274359
54	Ferroptosis	TP53	Human	Validated	P04637	29346757
55	Ferroptosis	CDKN1A	Human	Validated	P38936	29346757
56	Ferroptosis	MIR137	Human, mice	Validated	_NA_	29348676
57	Ferroptosis	SLC40A1	Mice	Validated	Q9NP59	29436589
58	Ferroptosis	GPX4	Mice	Validated	P36969	29436589
59	Ferroptosis	GPX4	Human	Deduced	P36969	29463878
60	Ferroptosis	ENPP2	Rat	Validated	Q13822	29551679
61	Ferroptosis	VDAC2	Human	Validated	P45880	29569437
62	Ferroptosis	FH	Human	Validated	P07954	29917289
63	Ferroptosis	CISD2	Human	Validated	Q8N5K1	29928961
64	Ferroptosis	SLC40A1	Human	Validated	Q9NP59	29949159
65	Ferroptosis	MIR9-1	Human	Validated	_NA_	30035324
66	Ferroptosis	MIR9-2	Human	Validated	_NA_	30035324
67	Ferroptosis	MIR9-3	Human	Validated	_NA_	30035324
68	Ferroptosis	CBS	Human, mice	Validated	P35520	30258181
69	Ferroptosis	NFE2L2	Human, mice	Validated	Q16236	30339884
70	Ferroptosis	SQSTM1	Human	Validated	Q13501	30339884
71	Ferroptosis	GPX4	Human	Validated	P36969	30524291
72	Ferroptosis	ISCU	Human	Validated	Q9H1K1	30557609
73	Ferroptosis	FTH1	Human	Validated	P02794	30557609
74	Ferroptosis	ACSL3	Human	Validated	O95573	30686757
75	Ferroptosis	OTUB1	Human, mice	Validated	Q96FW1	30709928
76	Ferroptosis	CD44	Human	Validated	P16070	30709928
77	Ferroptosis	LINC00336	Human	Validated	Q6ZUF6	30787392
78	Ferroptosis	STAT3	Human	Validated	P40763	30811078
79	Ferroptosis	BRD4	Human	Validated	O60885	30988278
80	Ferroptosis	PRDX6	Human	Validated	P30041	31036877
81	Ferroptosis	MIR17	Human	Validated	_NA_	31160087
82	Ferroptosis	SCD	Human, mice	Validated	O00767	31270077
83	Ferroptosis	SESN2	Human, mice	Validated	P58004	31323261
84	Ferroptosis	NF2	Human, mice	Validated	P35240	31341276

85	Ferroptosis	ARNTL	Human, mice	Validated	O00327	31355331
86	Ferroptosis	HIF1A	Human, mice	Validated	Q16665	31355331
87	Ferroptosis	JUN	Human, mice	Deduced	P05412	31394193
88	Ferroptosis	CA9	Human	Validated	Q16790	31442913
89	Ferroptosis	HSPA5	Human, mice	Validated	P11021	31456633
90	Ferroptosis	TMBIM4	Human, mice	Validated	Q9HC24	31507082
91	Ferroptosis	HSPA5	Human, mice	Predicted	P11021	31519193
92	Ferroptosis	PLIN2	Human, mice	Screened	Q99541	31520166
93	Ferroptosis	MIR212	Mice	Validated	_NA_	31533781
94	Ferroptosis	Fer1HCH	Drosophila	Predicted	H1UUD2	31568497
95	Ferroptosis	AIFM2	Human, mice	Validated	Q9BRQ8	31634899
96	Ferroptosis	AIFM2	Human, mice	Validated	Q9BRQ8	31634900
97	Ferroptosis	LAMP2	Human	Validated	P13473	31672277
98	Ferroptosis	ZFP36	Human, mice	Validated	P26651	31679460
99	Ferroptosis	GPX4	Human, mice, rat	Validated	P36969	31685805
100	Ferroptosis	PROM2	Human	Validated	Q8N271	31735663
101	Ferroptosis	CHMP5	Human, mice	Validated	Q9NZZ3	31761326
102	Ferroptosis	CHMP6	Human, mice	Validated	Q96FZ7	31761326
103	Ferroptosis	AKR1C1	Human	Validated	Q04828	31780644
104	Ferroptosis	AKR1C2	Human	Validated	P52895	31780644
105	Ferroptosis	AKR1C3	Human	Validated	P42330	31780644
106	Ferroptosis	CBS	Human	Validated	P35520	31819185
107	Ferroptosis	NFE2L2	Human	Validated	Q16236	31819185
108	Ferroptosis	CAV1	Human, mice	Validated	Q03135	31877357
109	Ferroptosis	GCH1	Human	Validated	P30793	31989025
110	Ferroptosis	SIRT3	Human	Validated	Q9NTG7	33377976
111	Ferroptosis	DAZAP1	_NA_	Validated	Q96EP5	33358859
112	Ferroptosis	PIR	Human, mice	Validated	O00625	33373853
113	Ferroptosis	GCLC	Human	Validated	P48506	33357455
114	Ferroptosis	FTL	Rat	Validated	P02792	33333054
115	Ferroptosis	HCAR1	Human, mice	Validated	Q9BXC0	33296645
116	Ferroptosis	SLC16A1	Human, mice	Validated	P53985	33296645
117	Ferroptosis	RRM2	Human	Validated	P31350	33372599
118	Ferroptosis	SCD	Human	Validated	O00767	33264619
119	Ferroptosis	NR4A1	Human	Deduced	P22736	33271455
120	Ferroptosis	PIK3CA	Human	Deduced	P42336	33229547
121	Ferroptosis	RPTOR	Human	Deduced	Q8N122	33229547
122	Ferroptosis	SREBF1	Human	Validated	P36956	33229547
123	Ferroptosis	SREBF2	Human	Validated	Q12772	33203734
124	Ferroptosis	FZD7	Human, mice	Validated	O75084	33172933
125	Ferroptosis	NFE2L2	Mice	Validated	Q16236	33197769
126	Ferroptosis	NFE2L2	Mice	Validated	Q16236	33152439
127	Ferroptosis	P4HB	Human	Deduced	P07237	33124817

128	Ferroptosis	NT5DC2	_NA_	Deduced	Q9H857	33124817
129	Ferroptosis	BCAT2	Human	Validated	O15382	33097833
130	Ferroptosis	HSF1	Human, mice	Validated	Q00613	33098823
131	Ferroptosis	PLA2G6	Human, mice	Validated	O60733	33087576
132	Ferroptosis	MIR424	Human	Validated	_NA_	33038905
133	Ferroptosis	PARK7	Human, mice	Validated	Q99497	33024240
134	Ferroptosis	FXN	Human	Validated	Q16595	33017621
135	Ferroptosis	SUV39H1	Human	Validated	O43463	33643820
136	Ferroptosis	ATF2	Human	Validated	P15336	33008584
137	Ferroptosis	CDKN1A	Human	Predicted	P38936	32979260
138	Ferroptosis	FTH1	Rat	Validated	P02794	32959272
139	Ferroptosis	NFE2L2	Mice	Validated	Q16236	33014271
140	Ferroptosis	STAT3	Mice	Validated	P40763	33014271
141	Ferroptosis	ACOT1	Mice	Validated	Q86TX2	32934217
142	Ferroptosis	NFE2L2	Rat	Validated	Q16236	32937103
143	Ferroptosis	ALDH3A2	Mice	Validated	P51648	32458004
144	Ferroptosis	NFE2L2	Mice	Validated	Q16236	32907551
145	Ferroptosis	STK11	Human	Validated	Q15831	32883948
146	Ferroptosis	FNDC5	Human, mice	Deduced	Q8NAU1	32997405
147	Ferroptosis	CircIL4R	Human	Validated	_NA_	32808701
148	Ferroptosis	CDH1	Human, mice	Validated	P12830	32896720
149	Ferroptosis	NFE2L2	Human	Validated	Q16236	32898818
150	Ferroptosis	MIR214	Human, mice	Validated	_NA_	32827544
151	Ferroptosis	NEDD4L	Human	Validated	Q96PU5	32811647
152	Ferroptosis	SQSTM1	Human	Validated	Q13501	32770451
153	Ferroptosis	TF	Human, mice	Validated	P02787	32374849
154	Ferroptosis	FTMT	Human	Validated	Q8N4E7	32810738
155	Ferroptosis	BRD2	Human	Deduced	P25440	32937365
156	Ferroptosis	BRD3	Human	Deduced	Q15059	32937365
157	Ferroptosis	BRD4	Human	Deduced	O60885	32937365
158	Ferroptosis	BRDT	Human	Deduced	Q58F21	32937365
159	Ferroptosis	SCD	Human, mice	Validated	O00767	32726752
160	Ferroptosis	SLC7A11	Human	Validated	Q9UPY5	32608563
161	Ferroptosis	DECR1	Human	Validated	Q16698	32686647
162	Ferroptosis	NFE2L2	Human	Validated	Q16236	32642794
163	Ferroptosis	GPX4	Rat	Validated	P36969	32634425
164	Ferroptosis	SLC7A11	Human	Validated	Q9UPY5	32621966
165	Ferroptosis	NFE2L2	Mice	Validated	Q16236	32601262
166	Ferroptosis	GLRX5	Human, mice	Validated	Q86SX6	32685019
167	Ferroptosis	GPX4	Human	Validated	P36969	32577235
168	Ferroptosis	NCOA3	Human	Deduced	Q9Y6Q9	32536370
169	Ferroptosis	NR5A2	Human	Deduced	O00482	32536370
170	Ferroptosis	GPX4	Human	Validated	P36969	32457486

171	Ferroptosis	MTOR	Human	Deduced	P42345	32457486
172	Ferroptosis	PANX2	Human	Validated	Q96RD6	32547072
173	Ferroptosis	RHEBP1	Human, mice	Validated	_NA_	32404875
174	Ferroptosis	TFAP2A	Human	Validated	P05549	32432738
175	Ferroptosis	CP	Human	Validated	P00450	32283255
176	Ferroptosis	SLC7A11	Human	Validated	Q9UPY5	32322334
177	Ferroptosis	ARF6	Human	Validated	P62330	32368394
178	Ferroptosis	GDF15	Human	Validated	Q99988	32209255
179	Ferroptosis	ABHD12	Human	Validated	Q8N2K0	32195565
180	Ferroptosis	PPP1R13L	Mice	Validated	Q8WUF5	32203170
181	Ferroptosis	TFAM	Human	Validated	Q00059	32186434
182	Ferroptosis	KDM3B	Human	Validated	Q7LBC6	32107878
183	Ferroptosis	RNF113A	Human	Validated	O15541	32152280
184	Ferroptosis	PARK7	Human	Validated	Q99497	32144268
185	Ferroptosis	AHCY	Human	Deduced	P23526	32144268
186	Ferroptosis	FXN	Human	Validated	Q16595	32169822
187	Ferroptosis	circ-TTBK2	Human	Validated	Q6IQ55	32196629
188	Ferroptosis	MIR522	Human	Validated	_NA_	32106859
189	Ferroptosis	IDH2	Human, mice	Validated	P48735	32089268
190	Ferroptosis	PPARA	Human	Validated	Q07869	32079652
191	Ferroptosis	NOS2	Mice	Validated	P35228	32080625
192	Ferroptosis	SIAH2	Human	Validated	O43255	32042051
193	Ferroptosis	RELA	Mice	Validated	Q04206	32015337
194	Ferroptosis	PRKAA2	Human	Validated	P54646	32029897
195	Ferroptosis	VDR	Mice	Validated	P11473	31996668
196	Ferroptosis	NEDD4	Human	Validated	P46934	31974380
197	Ferroptosis	FXN	Mice	Validated	Q16595	31974344
198	Ferroptosis	AIFM2	Human	Validated	Q9BRQ8	31964528
199	Ferroptosis	PRDX1	Human	Deduced	Q06830	31901729
200	Ferroptosis	AR	Human	Validated	P10275	31896509
201	Ferroptosis	CBS	Human	Validated	P35520	31819185
202	Ferroptosis	NFE2L2	Human	Validated	Q16236	31819185
203	Ferroptosis	CHMP5	Human	Validated	Q9NZZ3	31761326
204	Ferroptosis	CHMP6	Human	Validated	Q96FZ7	31761326
205	Ferroptosis	HMOX1	Mice	Validated	P09601	31740582
206	Ferroptosis	ZFP36	Human, mice	Validated	P26651	31679460
207	Ferroptosis	LAMP2	Human	Validated	P13473	31672277
208	Ferroptosis	MTF1	Human	Validated	Q14872	31320750
209	Ferroptosis	COPZ1	Human	Validated	P61923	33420375
210	Ferroptosis	NUPR1	Human	Validated	O60356	33510144
211	Ferroptosis	USP35	Human	Validated	Q9P2H5	33931967
212	Ferroptosis	HSF1	Human	Validated	Q00613	34223704
213	Ferroptosis	PROM2	Human	Validated	Q8N271	34223704

214	Ferroptosis	PLA2G6	Human	Validated	O60733	34131139
215	Ferroptosis	HIF1A	Human	Validated	Q16665	33895289
216	Ferroptosis	NEAT1	Human	Validated	_NA_	33730930
217	Ferroptosis	RRM2	Human	Validated	P31350	34648954
218	Ferroptosis	SLC7A11	Human	Validated	Q9UPY5	33991158
219	Ferroptosis	FTMT	Rat	Validated	Q8N4E7	33594527
220	Ferroptosis	PARP1	Human	Deduced	P09874	33722571
221	Ferroptosis	PARP2	Human	Deduced	Q9UGN5	33722571
222	Ferroptosis	PARP3	Human	Deduced	Q9Y6F1	33722571
223	Ferroptosis	PARP4	Human	Deduced	Q9UKK3	33722571
224	Ferroptosis	PARP6	Human	Deduced	Q2NL67	33722571
225	Ferroptosis	PARP8	Human	Deduced	Q8N3A8	33722571
226	Ferroptosis	PARP9	Human	Deduced	Q8IXQ6	33722571
227	Ferroptosis	PARP10	Human	Deduced	Q53GL7	33722571
228	Ferroptosis	PARP11	Human	Deduced	Q9NR21	33722571
229	Ferroptosis	PARP12	Human	Deduced	Q9H0J9	33722571
230	Ferroptosis	PARP14	Human	Deduced	Q460N5	33722571
231	Ferroptosis	PARP15	Human	Deduced	Q460N3	33722571
232	Ferroptosis	PARP16	Human	Deduced	Q8N5Y8	33722571
233	Ferroptosis	PDSS2	Human	Validated	Q86YH6	33929387
234	Ferroptosis	TXN	Mice	Validated	P10599	33634378
235	Ferroptosis	SENP1	Rat	Validated	Q9P0U3	33746578
236	Ferroptosis	PLA2G6	Human, mice	Validated	O60733	33542532
237	Ferroptosis	OIP5-AS1	Human	Validated	_NA_	34051661
238	Ferroptosis	MIR190A	Human	Validated	_NA_	34111670
239	Ferroptosis	FGF21	Mice	Validated	Q9NSA1	34530349
240	Ferroptosis	CREB1	Human	Deduced	P16220	33846793
241	Ferroptosis	CREB3	Human	Deduced	O43889	33846793
242	Ferroptosis	CREB5	Human	Deduced	Q02930	33846793
243	Ferroptosis	FTMT	Mice	Validated	Q8N4E7	33953171
244	Ferroptosis	GOT1	Human	Validated	P17174	34381026
245	Ferroptosis	TFRC	Mice	Validated	P02786	33955709
246	Ferroptosis	GPX4	Mice	Validated	P36969	34267193
247	Ferroptosis	MIR130B	Human	Validated	_NA_	34117611
248	Ferroptosis	BEX1	Human	Validated	Q9HBH7	33745298
254	Ferroptosis	ASAH2	Mice	Validated	Q9NR71	33547170
255	Ferroptosis	SCD	Mice	Validated	O00767	34030117
256	Ferroptosis	FABP4	Human	Validated	P15090	34030117
257	Ferroptosis	AKT1S1	Human	Deduced	Q96B36	33707434
258	Ferroptosis	MLST8	Human	Deduced	Q9BVC4	33707434
259	Ferroptosis	MTOR	Human	Deduced	P42345	33707434
260	Ferroptosis	RPTOR	Human	Deduced	Q8N122	33707434
261	Ferroptosis	CDH1	Human	Validated	P12830	34801472

262	Ferroptosis	SIRT1	Human	Validated	Q96EB6	34000515
263	Ferroptosis	TYRO3	Human, mice	Validated	Q06418	33855973
264	Ferroptosis	SIRT6	Human	Validated	Q8N6T7	34530350
265	Ferroptosis	TMSB4X	Human, rat	Deduced	P62328	34280397
266	Ferroptosis	TMSB4Y	Human, rat	Deduced	O14604	34280397
267	Ferroptosis	KIF20A	Human	Validated	O95235	33819186
268	Ferroptosis	ECH1	Human, mice	Validated	Q13011	33813878
272	Ferroptosis	circRHOT1	Human	Validated	_NA_	33686957
273	Ferroptosis	ETV4	Human	Validated	P43268	34283663
274	Ferroptosis	MEG8	Human	Validated	_NA_	33839417
275	Ferroptosis	VCP	Human	Validated	P55072	34033175
276	Ferroptosis	circ_0007142	Human	Validated	_NA_	33797091
277	Ferroptosis	ENPP2	Rats	Validated	Q13822	34296293
278	Ferroptosis	RBMS1	Human	Validated	P29558	34609966
279	Ferroptosis	KDM4A	Human	Validated	O75164	33689883
280	Ferroptosis	CBS	Human	Validated	P35520	34737229
281	Ferroptosis	MGST1	Human	Validated	P10620	33539732
282	Ferroptosis	circKIF4A	Human	Validated	_NA_	34153004
283	Ferroptosis	miR-7-5p	Human	Validated	_NA_	34361070
284	Ferroptosis	PRDX6	Mice	Validated	P30041	33894270
285	Ferroptosis	circ_0067934	Human	Validated	_NA_	34290668
286	Ferroptosis	MPC1	Human	Validated	Q9Y5U8	33741422
287	Ferroptosis	CHMP1A	Mice	Validated	Q9HD42	34426578
288	Ferroptosis	CAMKK2	Human	Validated	Q96RR4	34242660
289	Ferroptosis	SOX2	Human, mice	Validated	P48431	34385181
290	Ferroptosis	SRSF9	Human	Validated	Q13242	33609745
291	Ferroptosis	PROK2	Mice	Validated	Q9HC23	34244497
317	Ferroptosis	MIR4443	Human	Validated	_NA_	33781830
318	Ferroptosis	SIRT2	Mice	Validated	Q8IXJ6	34102645
319	Ferroptosis	circRNA1615	Mice	Validated	_NA_	34712388
320	Ferroptosis	MIR27A	Human	Validated	_NA_	34722314
321	Ferroptosis	MIR670	Human	Validated	_NA_	34323631
322	Ferroptosis	MEF2C	Human	Validated	Q06413	33984142
323	Ferroptosis	NF2	Human	Validated	P35240	33984142
324	Ferroptosis	CDH1	Human	Validated	P12830	33984142
325	Ferroptosis	HSPB1	Human	Validated	P04792	34791597
326	Ferroptosis	EZH2	Human	Validated	Q15910	34614259
327	Ferroptosis	PEDS1	Human	Validated	A5PLL7	33731874
328	Ferroptosis	SMPD1	Mice	Validated	P17405	34401974
329	Ferroptosis	ADAMTS13	Mice	Validated	Q76LX8	34666603
330	Ferroptosis	CDC25A	Human	Validated	P30304	34743185
331	Ferroptosis	G6PD	Human	Validated	P11413	34325001
332	Ferroptosis	SRSF9	Human	Validated	Q13242	34336668

333	Ferroptosis	CAV1	Human	Validated	Q03135	34874578
334	Ferroptosis	CircFNDC3B	Mice	Validated	_NA_	34434890
335	Ferroptosis	PPARD	Mice	Validated	Q03181	34649350
336	Ferroptosis	CISD2	Human	Validated	Q8N5K1	34485112
337	Ferroptosis	ENO3	Mice	Validated	P13929	33987359
338	Ferroptosis	SESN2	Mice	Validated	P58004	34482365
339	Ferroptosis	LCN2	Human	Validated	P80188	34921145
1264	Ferroptosis	MARCHF5	Rat	Validated	Q9NX47	34390730
1265	Ferroptosis	TRIB2	Human	Validated	Q92519	34315867
1266	Ferroptosis	DHODH	Human	Validated	Q02127	33981038
1267	Ferroptosis	SLC7A11	Human, mice	Validated	Q9UPY5	33681224
1268	Ferroptosis	MIR545	Human	Validated	_NA_	34954694
1269	Ferroptosis	OTUB1	Human	Validated	Q96FW1	34927544
1270	Ferroptosis	PDK4	Human	Validated	Q16654	33626342
1271	Ferroptosis	CircPVT1	Human	Validated	_NA_	34966683
1369	Ferroptosis	MIR9-3HG	Mice	Validated	_NA_	34953631
1370	Ferroptosis	ADIPOQ	Mice	Validated	Q15848	34859390
1371	Ferroptosis	circDTL	Human	Validated	_NA_	34621297
1372	Ferroptosis	GPX4	Mice	Validated	P36969	34133924
1373	Ferroptosis	mmu_circRNA_0000309	Mice	Validated	_NA_	34913724
1374	Ferroptosis	IL6	Human	Validated	P05231	34902522
1375	Ferroptosis	PTPN18	Human	Validated	Q99952	33642877
1376	Ferroptosis	FTH1	Human	Validated	P02794	34965856
1377	Ferroptosis	FTH1	Human	Validated	P02794	33997705
1378	Ferroptosis	FTL	Human	Validated	P02792	33997705
1379	Ferroptosis	LCN2	Human	Validated	P80188	34069743
1380	Ferroptosis	ABCC5	Human	Validated	O15440	34768109
1381	Ferroptosis	CISD3	Human	Validated	P0C7P0	34497268
1382	Ferroptosis	MS4A15	Mice	Validated	Q8N5U1	34663908
1383	Ferroptosis	LCN2	Human	Validated	P80188	34146401
1384	Ferroptosis	FURIN	Human, mice	Deduced	P09958	33640301
1385	Ferroptosis	circRHBG	Human	Validated	_NA_	34532485
1386	Ferroptosis	GALNT14	Human	Validated	Q96FL9	34643088
1387	Ferroptosis	KLHDC3	Human	Validated	Q9BQ90	34743205
1388	Ferroptosis	LINC01833	Human	Validated	_NA_	34728613
1389	Ferroptosis	circGFRA1	Human, mice	Validated	_NA_	34668628
1390	Ferroptosis	MAPKAP1	Mice	Deduced	Q9BPZ7	34949833
1391	Ferroptosis	MLST8	Mice	Deduced	Q9BVC4	34949833
1392	Ferroptosis	MTOR	Mice	Deduced	P42345	34949833
1393	Ferroptosis	PRR5	Mice	Deduced	P85299	34949833
1394	Ferroptosis	RICTOR	Mice	Deduced	Q6R327	34949833
1395	Ferroptosis	GSTM1	Human	Validated	P09488	34586745
1396	Ferroptosis	TERT	Mice	Validated	O14746	34716298

1397	Ferroptosis	circ0097009	Human	Validated	_NA_	33987373
1398	Ferroptosis	TMEM161B-DT	Human	Validated	_NA_	34689169
1399	Ferroptosis	circEPSTI1	Human	Validated	_NA_	33534779
1400	Ferroptosis	MIR18A	Human	Deduced	_NA_	33579899
1401	Ferroptosis	RARRES2	Human	Validated	Q99969	33757970
1402	Ferroptosis	USP11	Human	Validated	P51784	33531626

205 **Table S7. The ELAVL1-interacting mRNAs predicted by ENCORI database**

RBP	geneName	clusterNum	clipExpNum	clipIDnum	pancancerNum
ELAVL1	ACTB	5	39	248	16
ELAVL1	SFPQ	19	39	326	30
ELAVL1	HNRNPU	22	38	328	31
ELAVL1	LARP1	81	38	383	26
ELAVL1	SOD2	49	37	166	13
ELAVL1	BRD4	105	36	522	29
ELAVL1	DDX17	25	35	429	20
ELAVL1	HELLS	135	35	1145	29
ELAVL1	EIF2S3	25	35	262	24
ELAVL1	RDX	108	35	323	16
ELAVL1	CELF1	103	35	601	26
ELAVL1	ARF6	3	35	133	20
ELAVL1	PTP4A2	40	35	284	15
ELAVL1	PCBP2	33	35	464	24
ELAVL1	TNPO1	108	34	704	23
ELAVL1	TMED2	12	34	210	23
ELAVL1	HNRNPL	23	34	218	31
ELAVL1	MRPL19	36	34	248	28
ELAVL1	BIRC6	292	34	1261	24
ELAVL1	ARPP19	31	34	334	21
ELAVL1	MDM2	51	34	556	17
ELAVL1	SRSF1	6	34	197	32
ELAVL1	SLC7A1	110	34	434	23
ELAVL1	MARCHF6	100	34	744	21
ELAVL1	LUC7L2	91	34	667	26
ELAVL1	SETD5	115	34	818	24
ELAVL1	CAMTA1	234	34	468	17
ELAVL1	RCC2	50	34	259	31
ELAVL1	AC015813.2	22	34	354	11
ELAVL1	RPS10-NUDT3	177	34	563	17
ELAVL1	NUDT3	139	34	457	21
ELAVL1	MATR3	72	34	710	26
ELAVL1	MATR3	52	33	532	20
ELAVL1	DAZAP1	37	33	207	28
ELAVL1	TM9SF3	85	33	381	20
ELAVL1	ERC1	287	33	849	18
ELAVL1	RANBP1	4	33	64	25
ELAVL1	TNRC6B	157	33	517	27
ELAVL1	GINS1	44	33	216	31
ELAVL1	MED13	174	33	1467	22
ELAVL1	NUP155	94	33	428	29

ELAVL1	CSNK1A1	72	33	552	23
ELAVL1	NCL	15	33	186	31
ELAVL1	DR1	29	33	245	22
ELAVL1	CCNI	27	33	289	19
ELAVL1	TMPO	37	33	474	30
ELAVL1	HNRNPA2B1	46	33	485	29
ELAVL1	CANX	39	33	428	19
ELAVL1	SPCS3	13	33	212	19
ELAVL1	RLIM	37	33	249	24
ELAVL1	SRRM1	44	33	215	24
ELAVL1	SYNCRIP	44	33	468	29
ELAVL1	GATAD2B	99	33	604	28
ELAVL1	SKP2	51	33	185	30
ELAVL1	SREK1	52	33	350	24
ELAVL1	MSI2	301	33	1186	25
ELAVL1	DYRK1A	210	33	924	26
ELAVL1	TAOK1	142	33	722	24
ELAVL1	LSM12	25	33	125	26
ELAVL1	NFIA	348	33	1987	9
ELAVL1	SPDYA	49	33	231	19
ELAVL1	SRRM2	24	33	246	22
ELAVL1	RBPJ	136	33	637	17
ELAVL1	TOR1AIP2	45	33	379	21
ELAVL1	C16orf72	36	33	444	21
ELAVL1	ZNF703	4	33	151	18
ELAVL1	NF1	228	33	822	23
ELAVL1	ZNF121	24	33	243	31
ELAVL1	ATXN2	181	33	875	29
ELAVL1	SFT2D2	24	33	285	23
ELAVL1	PPP1CB	48	33	266	20
ELAVL1	AC008982.1	16	33	111	10
ELAVL1	FMC1-LUC7L2	89	33	542	9
ELAVL1	MARCKS	4	33	159	14
ELAVL1	CSDE1	57	32	724	20
ELAVL1	DCUN1D1	45	32	250	23
ELAVL1	MAP4	168	32	449	12
ELAVL1	ZFR	108	32	747	29
ELAVL1	GPBP1	115	32	426	25
ELAVL1	ELAVL1	22	32	139	32
ELAVL1	NUCKS1	51	32	710	28
ELAVL1	CDC42	61	32	275	18
ELAVL1	DPP8	60	32	200	20
ELAVL1	DLG1	244	32	949	22

ELAVL1	STARD7	20	32	168	25
ELAVL1	REST	40	32	182	20
ELAVL1	APLP2	69	32	220	13
ELAVL1	ATRX	223	32	691	21
ELAVL1	SEH1L	28	32	119	29
ELAVL1	KHSRP	10	32	140	32
ELAVL1	CBX5	47	32	416	31
ELAVL1	SCD	15	32	206	20
ELAVL1	NFAT5	144	32	720	20
ELAVL1	LONP2	77	32	174	20
ELAVL1	MAZ	4	32	106	29
ELAVL1	TNKS2	75	32	321	19
ELAVL1	LARP4B	158	32	1013	27
ELAVL1	TASOR2	98	32	597	25
ELAVL1	NUFIP2	43	32	467	23
ELAVL1	KPNB1	44	32	318	30
ELAVL1	PFN1	5	32	91	15
ELAVL1	PRKAR1A	35	32	210	20
ELAVL1	TMEM33	34	32	268	23
ELAVL1	DDX6	54	32	380	23
ELAVL1	CORO1C	52	32	175	23
ELAVL1	PTP4A1	15	32	229	20
ELAVL1	C6orf62	18	32	210	19
ELAVL1	ERBIN	157	32	706	21
ELAVL1	GOLPH3	68	32	405	26
ELAVL1	FAM172A	147	32	354	22
ELAVL1	GLS	103	32	683	19
ELAVL1	RO60	34	32	315	24
ELAVL1	PTBP3	99	32	401	24
ELAVL1	SET	10	32	144	30
ELAVL1	MED13L	437	32	2458	23
ELAVL1	HNRNPR	52	32	399	31
ELAVL1	HP1BP3	52	32	378	26
ELAVL1	MKLN1	221	32	855	16
ELAVL1	PHF10	25	32	162	28
ELAVL1	ZSWIM6	203	32	789	19
ELAVL1	RBM39	49	32	620	27
ELAVL1	ANKRD17	243	32	1323	24
ELAVL1	EIF5A	6	32	155	16
ELAVL1	ARGLU1	22	32	169	20
ELAVL1	SRPK2	274	32	1085	22
ELAVL1	TES	59	32	237	11
ELAVL1	TMOD3	84	32	401	18

ELAVL1	TMBIM6	28	32	263	17
ELAVL1	RERE	355	32	927	20
ELAVL1	G3BP1	47	32	393	28
ELAVL1	PARD3	455	32	1390	21
ELAVL1	LIN7C	13	32	176	26
ELAVL1	UHMK1	28	32	315	17
ELAVL1	MBNL1	196	32	969	15
ELAVL1	RMND5A	47	32	189	25
ELAVL1	ENAH	188	32	656	19
ELAVL1	ADK	498	32	2139	16
ELAVL1	SKI	74	32	287	20
ELAVL1	VMA21	10	32	175	28
ELAVL1	LARP4	85	32	575	24
ELAVL1	FUBP1	44	32	400	25
ELAVL1	PPP4R2	98	32	466	25
ELAVL1	UBXN7	65	32	250	26
ELAVL1	ARIH1	178	32	993	24
ELAVL1	GATAD2A	63	32	265	30
ELAVL1	CTNNB1	60	32	262	17
ELAVL1	PAFAH1B2	23	32	207	22
ELAVL1	PDE3A	51	32	150	11
ELAVL1	ATP2A2	77	32	319	24
ELAVL1	RSRC1	270	32	950	28
ELAVL1	RAB6A	81	32	303	20
ELAVL1	CSTF3	103	32	512	31
ELAVL1	BASP1	91	32	271	16
ELAVL1	HNRNPA0	9	32	138	31
ELAVL1	UBE2N	50	32	260	29
ELAVL1	PTPN11	86	32	343	28
ELAVL1	PITPNB	89	32	375	24
ELAVL1	DAZAP2	12	32	81	18
ELAVL1	SP1	27	32	232	22
ELAVL1	NAP1L1	65	32	731	24
ELAVL1	HDAC2	56	32	205	32
ELAVL1	STRN3	161	32	893	21
ELAVL1	HMGN2	7	32	72	26
ELAVL1	CAPZA2	63	32	214	17
ELAVL1	EXOSC6	2	32	182	25
ELAVL1	ARPC4-TTLL3	27	32	118	20
ELAVL1	CUX1	243	32	1010	19
ELAVL1	AC022826.2	150	32	504	4
ELAVL1	TAF15	88	32	347	29
ELAVL1	AC073283.3	71	32	323	5

ELAVL1	AC017083.3	116	32	380	11
ELAVL1	AL135905.2	13	32	225	0
ELAVL1	PAFAH1B1	105	31	463	18
ELAVL1	SEC63	107	31	341	23
ELAVL1	KPNA6	56	31	253	25
ELAVL1	NSUN2	41	31	142	27
ELAVL1	THRAP3	80	31	317	27
ELAVL1	PUM2	148	31	1016	27
ELAVL1	RC3H2	58	31	261	21
ELAVL1	WNK1	175	31	799	21
ELAVL1	NFYC	55	31	126	24
ELAVL1	EIF4G3	286	31	1052	23
ELAVL1	UBE2K	109	31	769	26
ELAVL1	SAR1A	26	31	242	15
ELAVL1	GSK3B	235	31	905	26
ELAVL1	XPO1	73	31	724	29
ELAVL1	PDS5B	219	31	845	24
ELAVL1	SRPK1	102	31	643	27
ELAVL1	ERH	24	31	158	26
ELAVL1	EIF5	15	31	123	19
ELAVL1	ACIN1	32	31	239	26
ELAVL1	FMR1	38	31	167	22
ELAVL1	ZFAND1	37	31	205	23
ELAVL1	GPI	25	31	147	21
ELAVL1	MTPN	53	31	238	18
ELAVL1	H2AZ2	20	31	258	28
ELAVL1	TMEM248	26	31	177	21
ELAVL1	PSMD11	44	31	213	29
ELAVL1	TMEM97	4	31	91	23
ELAVL1	DCUN1D4	78	31	269	19
ELAVL1	UBE2D3	69	31	428	21
ELAVL1	MLEC	14	31	172	21
ELAVL1	PTGES3	33	31	242	29
ELAVL1	MRPS27	80	31	225	25
ELAVL1	SUB1	36	31	211	22
ELAVL1	C5orf15	14	31	182	20
ELAVL1	UBE3A	111	31	423	24
ELAVL1	SRSF11	58	31	361	26
ELAVL1	PTBP2	143	31	704	17
ELAVL1	ATL2	93	31	369	22
ELAVL1	UFM1	20	31	145	19
ELAVL1	ZMYM2	175	31	1155	21
ELAVL1	AMD1	35	31	193	19

ELAVL1	BAZ2B	139	31	424	21
ELAVL1	SDC4	8	31	81	10
ELAVL1	ATXN1	274	31	769	16
ELAVL1	KDM5C	36	31	135	26
ELAVL1	ILF3	52	31	337	31
ELAVL1	ZRANB2	21	31	222	22
ELAVL1	MPRIP	109	31	366	19
ELAVL1	TMED7	12	31	149	24
ELAVL1	DYNC1LI2	28	31	212	20
ELAVL1	RC3H1	107	31	707	22
ELAVL1	KDELR2	25	31	213	16
ELAVL1	BRIP1	256	31	1983	29
ELAVL1	TRA2B	36	31	290	29
ELAVL1	WDR33	138	31	619	28
ELAVL1	ETNK1	79	31	820	27
ELAVL1	TMTC3	60	31	303	23
ELAVL1	TMX1	21	31	184	20
ELAVL1	ZFHX3	231	31	936	20
ELAVL1	CLTC	102	31	558	23
ELAVL1	TP53	14	31	130	22
ELAVL1	SERBP1	28	31	246	27
ELAVL1	CDKAL1	303	31	613	26
ELAVL1	PHIP	159	31	740	24
ELAVL1	CACUL1	89	31	319	18
ELAVL1	TIAL1	33	31	336	24
ELAVL1	C18orf25	73	31	243	23
ELAVL1	PPP1R15B	9	31	180	22
ELAVL1	ELK4	37	31	316	18
ELAVL1	RBM15	5	31	147	22
ELAVL1	DAGLB	41	31	243	12
ELAVL1	TRA2A	33	31	284	21
ELAVL1	TMEM65	77	31	321	16
ELAVL1	RNF169	57	31	239	21
ELAVL1	PBX3	184	31	818	14
ELAVL1	ANKRD11	233	31	985	21
ELAVL1	ATXN2L	20	31	96	28
ELAVL1	PTK2	312	31	1237	24
ELAVL1	ROBO1	439	31	1277	16
ELAVL1	ARL6IP1	14	31	159	29
ELAVL1	ATF7IP	158	31	697	24
ELAVL1	PTEN	124	31	715	15
ELAVL1	PAWR	158	31	889	20
ELAVL1	TBL1XR1	222	31	1300	26

ELAVL1	R3HDM2	157	31	804	21
ELAVL1	SOCS4	20	31	171	20
ELAVL1	C5orf24	14	31	166	19
ELAVL1	RAD51B	427	31	1638	15
ELAVL1	POMK	29	31	277	21
ELAVL1	JPT1	39	31	190	21
ELAVL1	NHLRC2	56	31	288	25
ELAVL1	TRIM33	186	31	1237	26
ELAVL1	CDC42SE1	9	31	100	19
ELAVL1	HELZ	219	31	1008	23
ELAVL1	TXNRD1	80	31	284	21
ELAVL1	TLK1	163	31	527	25
ELAVL1	TOP1	130	31	608	28
ELAVL1	RAB12	44	31	187	18
ELAVL1	CSNK1E	18	31	99	23
ELAVL1	NUDT19	24	31	147	27
ELAVL1	PPP3R1	85	31	315	28
ELAVL1	ARPC4	10	31	83	14
ELAVL1	MRPS6	96	31	585	18
ELAVL1	TMED7-TICAM2	47	31	282	18
ELAVL1	LSM14A	70	31	309	28
ELAVL1	AL589666.1	69	31	495	7
ELAVL1	AC104109.3	68	31	252	14
ELAVL1	AC008695.1	327	31	1051	1
ELAVL1	AL031681.2	28	31	177	0
ELAVL1	AP001931.2	100	31	454	0
ELAVL1	CDC27	84	30	326	26
ELAVL1	ZFX	73	30	388	23
ELAVL1	AP2B1	126	30	282	25
ELAVL1	SPAG9	153	30	412	19
ELAVL1	BAZ1B	90	30	339	30
ELAVL1	ZNF207	32	30	359	29
ELAVL1	WWTR1	73	30	209	14
ELAVL1	BCLAF1	45	30	362	28
ELAVL1	TRIO	293	30	1283	22
ELAVL1	FOXN3	86	30	234	14
ELAVL1	EIF2AK2	38	30	144	25
ELAVL1	PPP1R12A	183	30	669	22
ELAVL1	ZC3H11A	76	30	480	24
ELAVL1	QSER1	96	30	363	29
ELAVL1	WAPL	115	30	406	25
ELAVL1	PKN2	149	30	470	20
ELAVL1	EVI5	184	30	386	16

ELAVL1	CBFB	62	30	269	26
ELAVL1	FNDC3B	217	30	570	16
ELAVL1	MLLT10	233	30	920	24
ELAVL1	KAT6A	114	30	317	20
ELAVL1	ZMPSTE24	31	30	153	22
ELAVL1	B4GALT1	34	30	176	9
ELAVL1	FKBP1A	30	30	159	12
ELAVL1	SNX5	33	30	275	25
ELAVL1	PAPOLA	77	30	701	29
ELAVL1	RBM27	97	30	327	28
ELAVL1	ALKBH5	11	30	156	30
ELAVL1	HNRNPC	81	30	874	31
ELAVL1	ARCN1	22	30	181	19
ELAVL1	HNRNPH3	12	30	212	29
ELAVL1	DICER1	75	30	323	24
ELAVL1	YY1	56	30	240	30
ELAVL1	ARHGAP5	90	30	454	20
ELAVL1	STK4	96	30	284	22
ELAVL1	PRELID3B	8	30	111	24
ELAVL1	CSNK2A1	74	30	301	29
ELAVL1	PGK1	73	30	192	19
ELAVL1	SMS	13	30	61	23
ELAVL1	GSPT1	61	30	316	24
ELAVL1	GSR	27	30	136	16
ELAVL1	PTPRS	41	30	224	22
ELAVL1	LMBR1	179	30	656	27
ELAVL1	SPIN1	101	30	483	27
ELAVL1	TMEM245	105	30	418	19
ELAVL1	CCNY	159	30	466	22
ELAVL1	AKAP10	89	30	331	19
ELAVL1	CCND1	11	30	121	14
ELAVL1	EIF4G2	13	30	246	27
ELAVL1	NAA25	101	30	585	28
ELAVL1	SLC38A1	114	30	899	17
ELAVL1	CNOT2	154	30	924	21
ELAVL1	CPSF6	38	30	418	30
ELAVL1	SUDS3	42	30	207	31
ELAVL1	SRSF9	12	30	113	28
ELAVL1	QKI	168	30	742	19
ELAVL1	MRPS30	15	30	133	26
ELAVL1	CLINT1	80	30	287	22
ELAVL1	CNOT6	99	30	371	28
ELAVL1	TCERG1	82	30	518	29

ELAVL1	SLC25A36	52	30	345	24
ELAVL1	FXR1	83	30	459	25
ELAVL1	MOB1A	21	30	241	28
ELAVL1	GPD2	66	30	190	22
ELAVL1	CENPA	27	30	258	30
ELAVL1	ATF2	119	30	353	22
ELAVL1	SUMO1	36	30	122	27
ELAVL1	RAB14	34	30	179	22
ELAVL1	RBM25	82	30	424	25
ELAVL1	MTHFD1L	155	30	387	22
ELAVL1	SEPTIN7	96	30	275	20
ELAVL1	CBX3	18	30	140	26
ELAVL1	SMU1	34	30	151	28
ELAVL1	SRSF6	10	30	155	23
ELAVL1	SOX4	6	30	128	26
ELAVL1	SSR1	43	30	289	26
ELAVL1	NUP153	122	30	530	27
ELAVL1	DEK	51	30	350	28
ELAVL1	CNOT1	147	30	517	24
ELAVL1	PANK2	47	30	333	23
ELAVL1	OVOL2	26	30	182	13
ELAVL1	RAP1B	77	30	444	18
ELAVL1	CALU	38	30	250	20
ELAVL1	GNL3L	69	30	356	23
ELAVL1	TULP4	138	30	435	24
ELAVL1	ACLY	43	30	139	28
ELAVL1	EXOC4	268	30	707	17
ELAVL1	KHDC4	28	30	166	22
ELAVL1	XPO4	132	30	531	26
ELAVL1	LARS1	90	30	360	26
ELAVL1	VHL	11	30	136	26
ELAVL1	SORT1	60	30	186	14
ELAVL1	PUM1	179	30	745	26
ELAVL1	BTF3L4	35	30	215	26
ELAVL1	WDR36	54	30	206	25
ELAVL1	CTDSPL2	93	30	339	23
ELAVL1	LRPPRC	140	30	713	26
ELAVL1	SEPTIN11	75	30	313	21
ELAVL1	AEBP2	282	30	892	29
ELAVL1	TCF12	253	30	715	26
ELAVL1	TLE3	56	30	287	12
ELAVL1	ANP32A	53	30	255	30
ELAVL1	IQGAP1	55	30	183	16

ELAVL1	SS18	89	30	507	23
ELAVL1	COP1	249	30	945	26
ELAVL1	ANP32E	14	30	196	27
ELAVL1	TPM3	35	30	219	26
ELAVL1	SRP9	19	30	116	24
ELAVL1	CDC42BPA	267	30	667	16
ELAVL1	LBR	29	30	207	28
ELAVL1	CALM2	26	30	215	21
ELAVL1	RHBDD1	80	30	163	18
ELAVL1	MMS22L	169	30	664	27
ELAVL1	TMEM209	38	30	185	27
ELAVL1	NCAPG2	86	30	410	29
ELAVL1	KDM6A	272	30	884	23
ELAVL1	NONO	22	30	185	29
ELAVL1	RBMX	42	30	179	29
ELAVL1	NSD3	104	30	315	26
ELAVL1	NFIB	193	30	631	13
ELAVL1	PDCD4	36	30	159	12
ELAVL1	WWC2	126	30	379	18
ELAVL1	DST	191	30	492	14
ELAVL1	FARP1	64	30	176	20
ELAVL1	ASAP1	160	30	428	18
ELAVL1	CEBPG	11	30	139	26
ELAVL1	PDIA4	19	30	108	24
ELAVL1	ZFAND3	354	30	1602	24
ELAVL1	ATAD2	111	30	720	29
ELAVL1	SON	49	30	287	21
ELAVL1	GPBP1L1	55	30	237	20
ELAVL1	AGPAT3	60	30	238	21
ELAVL1	CMPK1	28	30	171	22
ELAVL1	FAM102B	40	30	152	19
ELAVL1	U2SURP	66	30	471	28
ELAVL1	WDR43	74	30	448	28
ELAVL1	ZNF148	166	30	666	24
ELAVL1	FOXK1	54	30	321	22
ELAVL1	PDZD8	104	30	320	22
ELAVL1	NUDT21	22	30	138	29
ELAVL1	MIDN	7	30	96	22
ELAVL1	MAPK1IP1L	26	30	219	27
ELAVL1	HNRNPH1	19	30	203	28
ELAVL1	CNBP	20	30	168	19
ELAVL1	YWHAG	23	30	228	29
ELAVL1	FRMD5	78	30	167	11

ELAVL1	JMJD1C	365	30	1312	20
ELAVL1	GNG12	61	30	230	18
ELAVL1	DCP2	47	30	255	24
ELAVL1	SP3	95	30	519	26
ELAVL1	LCLAT1	144	30	399	27
ELAVL1	MFSD4B	60	30	311	18
ELAVL1	DAG1	41	30	184	20
ELAVL1	SMARCC1	187	30	603	28
ELAVL1	MOB1B	79	30	194	21
ELAVL1	GRB2	81	30	335	22
ELAVL1	RFLNA	68	30	256	10
ELAVL1	ZNF664	41	30	217	27
ELAVL1	RCC1	36	30	275	25
ELAVL1	PAK2	47	30	317	28
ELAVL1	TTC3	115	30	419	23
ELAVL1	RBM12B	17	30	169	28
ELAVL1	EFNA5	349	30	1986	15
ELAVL1	ANKS1B	100	30	215	16
ELAVL1	LSAMP	72	30	158	12
ELAVL1	KPNA4	71	30	372	26
ELAVL1	SUMO2	23	30	149	27
ELAVL1	SF3B3	70	30	405	28
ELAVL1	HNRNPAB	10	30	80	30
ELAVL1	FAR1	80	30	451	23
ELAVL1	MRPL42	48	30	304	28
ELAVL1	OGA	42	30	403	16
ELAVL1	CTNND1	67	30	384	20
ELAVL1	LRBA	389	30	1061	22
ELAVL1	SLC5A3	52	30	406	21
ELAVL1	C5orf51	11	30	163	26
ELAVL1	ANKRD28	207	30	803	21
ELAVL1	NRAS	14	30	156	24
ELAVL1	FNIP1	138	30	566	18
ELAVL1	N4BP2L2	147	30	823	15
ELAVL1	EIF5AL1	2	30	78	6
ELAVL1	AC110275.1	48	30	278	2
ELAVL1	AL138752.2	111	30	282	12
ELAVL1	ZBED6	76	30	480	19
ELAVL1	AC138811.2	13	30	154	23
ELAVL1	AL136531.2	36	30	166	16
ELAVL1	AC091959.1	120	30	354	12
ELAVL1	UHRF1	31	30	136	31
ELAVL1	AC118553.2	126	30	391	0

ELAVL1	AC013717.1	107	30	487	0
ELAVL1	AC087721.2	85	30	366	0
