# Supplementary material for: Circular RNA TFRC/SCD1 mRNA interaction regulates ferroptosis and metastasis in gastric cancer
Source: Cell Death Dis. 2025 Jun 5;16(1):436. doi: 10.1038/s41419-025-07759-x (PMC12141735; doi:10.1038/s41419-025-07759-x)

# The original uncropped western blots of Figures 5B, 5G, 5H, 5M, S5A, and S7A.

**Figure 5B**

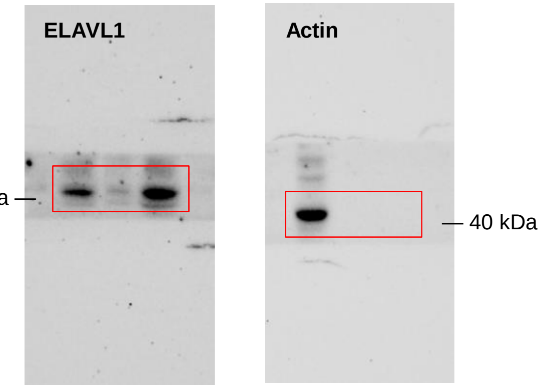


**Figure 5G**

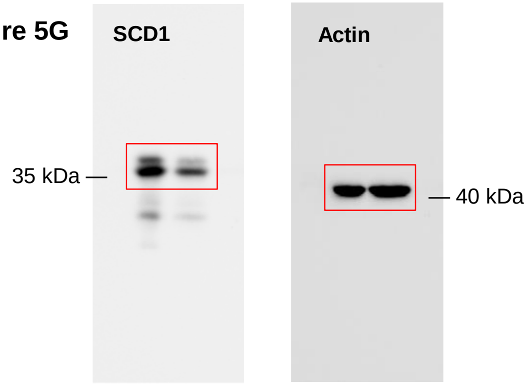


**Figure 5H**

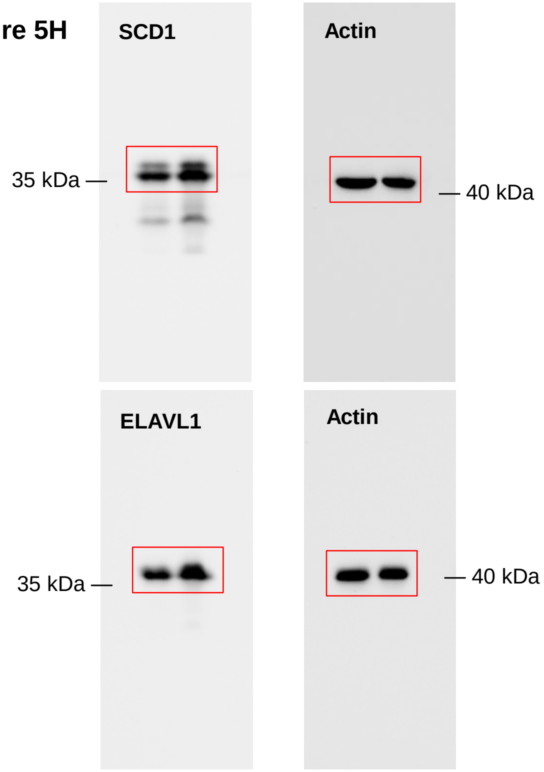


**Figure 5M**

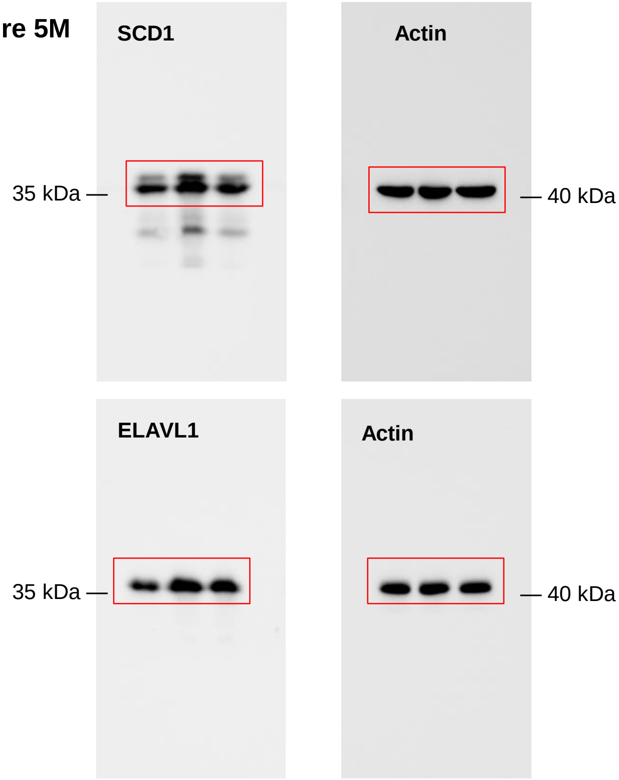


**Figure S5A**

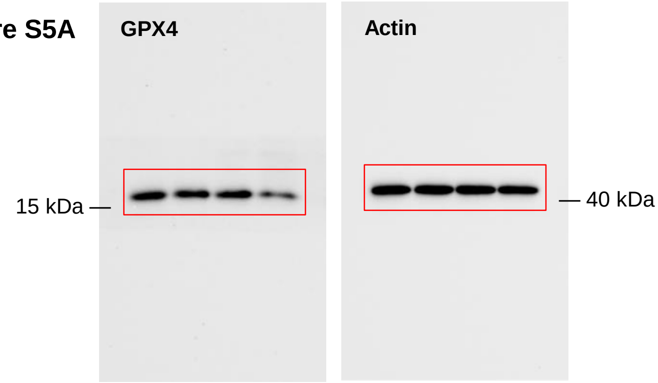


**Figure S7A**

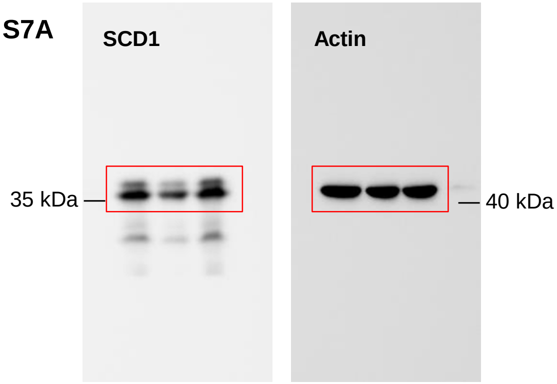

Supplement: Supplementary file 2 — Original data (full uncropped western blots) [file 41419_2025_7759_MOESM2_ESM.pdf]
