# Supplementary material for: Circular RNA TFRC/SCD1 mRNA interaction regulates ferroptosis and metastasis in gastric cancer
Source: Cell Death Dis. 2025 Jun 5;16(1):436. doi: 10.1038/s41419-025-07759-x (PMC12141735; doi:10.1038/s41419-025-07759-x)
Supplement: Supplementary file 3 — Original data (qPCR data) [file 41419_2025_7759_MOESM3_ESM.pdf]

Figure 1D

Adjacent	Tumor
0.331548892	2.22090815
0.460825846	2.760974282
0.968767005	2.236355788
0.219500106	1.810200786
1.370043437	0.903891576
0.116814684	6.20469711
0.954418573	2.797512877
0.693656606	2.906822821
0.923535419	5.379432128
0.759953678	3.432450102
0.90798981	4.372054861
1.796699932	1.236465198
3.067536346	0.946859081
2.406189428	2.678121909
0.956243494	2.954515949
0.846528204	3.097036317
0.325853081	3.857202711
1.300639942	1.428223374
3.072935441	1.158528503
1.726146565	2.880394679
3.229785724	3.294951953
0.343699971	2.197037202
0.76183077	2.914181591
0.853054598	1.329504169
1.014767858	3.133949591
0.761576013	0.741273491
0.74599826	2.867922262
0.748195268	0.652060541
0.989312418	2.173478642
0.546418344	2.865811515
0.946858144	2.314736489
0.141044433	2.877626248
0.479373357	1.722162924
1.492047576	2.885374258
0.325159985	1.874037379
0.913854058	3.306334601
0.303144557	2.822330397
0.901220567	2.121384186
1.1250631	1.741108269
0.786259704	3.332324883
0.498272176	5.064634745
0.88813458	1.469174661

Figure 1E

Healthy	GC
1.383588	0.820811
0.104674	2.618932
0.880202	0.586002
2.953665	3.929445
1.959302	0.627223
5.057782	0.583975
0.898535	7.185879
0.053252	4.894267
0.027145	2.34401
1.916928	0.701725
0.251602	4.901908
1.122842	1.555146
1.000249	2.561504
0.347039	3.77771
0.078132	2.022173
0.72802	0.922729
0.433462	5.678893
0.073051	2.659484
0.078027	9.183083
0.652521	4.090129
	7.566697
	3.706358
	4.436127
	5.391582
	3.711624
	3.138064
	3.233817
	4.239748
	3.439146
	4.391502
	2.502821
	3.402694
	2.412257
	3.946696
	2.024878
	2.200505
	0.851356
	2.27311
	6.452073
	2.22322

Figure 1F

˂ 5 cm	≥ 5 cm
6.185878727	9.183082775
5.678892946	7.894266605
4.901908239	6.452073327
4.391501751	5.566697412
4.239748096	5.391582119
3.70635779	4.436126725
3.439146219	4.090129204
3.402694268	3.946695504
3.233816623	3.929445053
3.138063914	3.777709745
2.502820718	3.711623709
2.412257283	2.659483705
2.344009716	2.618931907
2.273109732	2.561504471
2.223220059	2.200505137
2.022173305	2.024877652
1.555146459	
0.922728773	
0.85135618	
0.820811222	
0.70172543	
0.627223038	
0.586002429	
0.583975015	

Figure 1G

None	Metastasis
6.452073327	9.183082775
2.024877652	7.894266605
4.901908239	5.566697412
5.678892946	6.185878727
4.239748096	4.391501751
4.090129204	3.929445053
3.946695504	3.70635779
3.402694268	2.502820718
3.233816623	2.412257283
3.138063914	2.223220059
2.561504471	
2.200505137	
2.344009716	
2.273109732	
3.439146219	
2.022173305	
1.555146459	
0.922728773	
0.85135618	
0.820811222	
0.70172543	
0.627223038	
0.586002429	
0.583975015	
5.391582119	
4.436126725	
3.777709745	
3.711623709	
2.659483705	
2.618931907	

Figure 1J

GES-1	AGS	HGC-27	MKN-45	MKN-28
1.047294	3.394816	4.834388	1.401204	2.386
0.874583	4.542018	5.749089	1.848899	1.861292
1.091768	4.179509	6.291192	1.450617	2.032925

Figure 2B

	cDNA			gDNA		
**DP**	0.890899	0.981686	1.143402	0.004405	0.00397	0.005732
**CP**	0.97716	0.816014	1.254112	0.846745	1.132884	1.042466

Figure 2C

	cDNA			gDNA		
**DP**	1.122462	0.874583	1.018656	0.003578	0.006449	0.004656
**CP**	1	1.101905	0.907519	1.401204	1.0848899	1.250617

Figure 2G

	circTFRC			*TFRC* mRNA		
**0**	1.148698	0.959264	0.907519	1.01865581	1.047294123	0.9373545
**2**	1.197478705	1.021012126	1.132883885	0.720298431	0.827405623	0.6767368
**4**	1.021012126	0.773782497	0.858565436	0.391386708	0.50580972	0.4023901
**6**	0.901250463	0.901250463	0.773782497	0.355190935	0.452712381	0.3833321
**8**	0.846745312	0.972654947	0.895025071	0.3360311	0.367716716	0.2966157
**12**	0.687770909	0.790041312	0.920187651	0.296615659	0.388683203	0.3503009
**24**	0.5302478	0.7778861	0.6900336	0.1430646	0.10471937	0.2481098

Figure 2H

	circTFRC			*TFRC* mRNA		
**0**	1.104454001	1.052144848	0.860551437	0.926588062	1.071773463	1.0069556
**2**	1.09682498	1.208597056	1.037659659	0.543367431	0.50697974	0.476319
**4**	1.074252648	0.935191248	0.903335201	0.47963206	0.408951029	0.3737123
**6**	0.825496117	0.878633452	0.897095409	0.353553391	0.420448208	0.408951
**8**	0.723634619	0.759611332	0.995389679	0.411795509	0.366021424	0.3164391
**12**	0.854607174	0.842841545	0.73883972	0.363493129	0.314253344	0.2300469
**24**	0.77667	0.82667	0.69667	0.1398333	0.1824333	0.2432333

Figure 2I

	RNase R-			RNase R+		
**CircTFRC**	1.189207115	0.858565436	0.979420298	1.132883885	0.979420298	1.1566882
***TFRC* mRNA**	0.997692177	0.997692177	1.004631674	0.117984289	0.122994582	0.1238501
***GAPDH* mRNA**	1.016304932	1.009284801	0.974904856	0.024918022	0.024069222	0.024918

Figure 2J

	RNase R-			RNase R+		
**CircTFRC**	0.950439478	1.099362113	0.957050307	1.089370308	1.316462719	1.1716192
***TFRC* mRNA**	1.016304932	1.009284801	0.974904856	0.03803136	0.037248686	0.0385623
***GAPDH* mRNA**	0.995389679	1.002313162	1.002313162	0.007669421	0.007205595	0.0070573

Figure 3A

si-NC	si-circTFRC#1	si-circTFRC#2
1.032876	0.328356	0.224274
0.899171	0.293887	0.177186
1.076738	0.361817	0.15749

Figure 3B

si-NC	si-circTFRC#1	si-circTFRC#2
1.132884	0.360149	0.269807
0.986233	0.322343	0.213159
0.895025	0.261824	0.189465

Figure 5C

	IgG			anti-ELAVL1		
**circTFRC**	1.004632	1.107009	0.899171	18.54982	22.05958	15.92623
GAPDH	1.135504	0.749154	1.175548	1.347234	1.802501	1.458639
**circZNF609**	0.858565	1.197479	0.972655	10.17295	7.871653	8.613901

Figure 5E

	sh-NC			sh-circTFRC		
DAZAP1	0.943874	1.076738	0.983957	0.946058	0.852635	0.7615572
ARF6	0.90125	1.189207	0.933033	1.259921	1.135504	1.0919794
ATF2	1.122462	1.018656	0.874583	0.823374	0.922316	0.6658796
HELLS	1.140764	0.97942	0.895025	0.843367	0.898739	0.7664419
BRD4	0.852635	1.125058	1.042466	1.191958	1.074253	1.2755724
TP53	0.890899	1.143402	0.981686	0.597358	0.658231	0.7227248
SRSF9	1.081725	0.928731	0.99539	1.059463	0.954842	0.8893618
SCD1	1.0792282	1	0.9265881	0.315246	0.267752	0.3371263

Figure 5F

	Ctrl cDNA			*ELAVL1* cDNA		
DAZAP1	0.899171	1.018656	1.091768	1.130269	0.880666	1.1782671
ARF6	0.983957	0.911722	1.114709	1.502501	1.741101	1.2570134
ATF2	0.850667	1.254112	0.937354	0.776627	0.801984	0.8391589
HELLS	0.986233	1.117287	0.907519	1.239708	0.965936	1.2923528
BRD4	0.961483	0.922316	1.127661	0.97716	1.162046	1.2716192
TP53	0.880666	1.170128	0.97041	1.006956	1.197479	1.3103934
SRSF9	0.9592641	1.1328839	0.9201877	0.828507	0.8971	0.7551967
SCD1	0.905425	0.950439	1.162046	1.874544	2.418429	2.4679845

Figure 5I

	IgG			anti-ELAVL1		
SCD1	1.089249	0.928731	0.988514	10.85283	8.938297	11.95879
GAPDH	1.325619	0.844791	0.89296	1.200249	0.909618	1.242575
CKAP5	1.016305	0.866537	1.135504	5.063026	7.361501	4.531536

Figure 5J

	Ctrl cDNA + sh-NC			*ELAVL1* cDNA + sh-NC			Ctrl cDNA + sh-circTFRC			*ELAVL1* cDNA + sh-circTFRC		
**0**	110.7009	91.17225	99.08006	95.48416	105.2145	99.53897	87.66057	109.4294	104.2466	85.6584	114.6047	101.8656
**1**	92.015618	76.147512	82.636617	92.051506	101.38357	105.12608	72.111057	67.08271	76.41952	82.24786	99.91333	95.23586
**2**	82.025088	70.51807	66.478702	102.057336	97.418293	83.402454	52.136656	62.88406	47.94202	88.27288	91.90226	79.72867
**4**	62.030713	49.477708	55.87801	82.083647	72.82592	76.744176	32.159984	17.64068	25.57622	75.28168	60.26546	57.19259
**8**	36.285528	28.52755	23.983108	72.098685	69.905888	61.38645	17.160606	12.361	9.30313	32.0398	19.78909	28.58749

Figure 5K

	Ctrl probe			CircTFRC probe		
**circTFRC**	0.963707	1.170128	0.886791	148.3986	114.8282	125.6558
TFRC	0.90125	1.189207	0.933033	1.1386	1.861292	1.632925
SCD1	0.814131	1.074253	1.143402	15.45498	10.33882	13.8326
GAPDH	0.886791	1.032876	1.091768	1.467472	1.112136	1.519223

Figure 5L

	sh-NC			sh-circTFRC		
IgG	0.838956	1.203025	0.990801	1.757267	1.277509	2.255322
anti-ELAVL1	1.132884	0.913831	0.965936	0.162292	0.122995	0.206851

Figure 7D

	Ctrl siRNA			CircTFRC siRNA		
**circTFRC**	0.972655	0.888843	1.156688	0.23374	0.35035	0.259463
***SCD1* mRNA**	1.004632	1.107009	0.899171	0.437291	0.336031	0.509212

Figure S1D

Healthy	Adjacent
1.096825	1.094294
0.890899	1.385109
1.023374	1.274561

Figure S1E

circRNA_103554	circRNA_104902	circRNA_102415	circRNA_102503	circRNA_103110	circRNA_103237
0.866537	0.185137	0.105843	0.793701	0.503478	0.260094
0.981686	0.153538	0.089003	0.658231	0.423373	0.386891
1.175548	0.198425	0.07911	0.550667	0.376312	0.343885

Figure S1F

circRNA_103554	circRNA_104902	circRNA_102415	circRNA_102503	circRNA_103110	circRNA_103237
0.907519	0.121301	0.193893	0.497695	0.468677	0.684343
0.959264	0.102002	0.160799	0.579682	0.394109	0.753782
1.148698	0.090664	0.207809	0.634342	0.326842	0.846745

Figure S2C

	Nucleus			Cytoplasm		
GAPDH	16.05271	15.68259	16.52526	83.94729	84.31741	83.47474
U6	70.01369	68.53859	69.86797	29.98631	31.46141	30.13203
**CircTFRC**	13.07206	11.57577	10.5533	86.92794	88.42423	89.4467

Figure S2D

	Nucleus			Cytoplasm		
GAPDH	18.38817	17.37068	16.68524	81.61183	82.62932	83.31476
U6	85.38616	84.68055	85.21232	14.61384	15.31945	14.78768
**CircTFRC**	16.78182	13.33686	13.74254	83.21818	86.66314	86.25746

Figure S3A

	si-NC			si-circTFRC#1			si-circTFRC#2		
***TFRC* mRNA**	1.032876	0.899171	1.076738	0.926588	0.732043	0.80107	0.897095	0.708742	0.929961
**circ_0068631**	1.132884	0.986233	0.895025	1.236847	1.107009	1.362888	1.313425	1.175548	1.047269

Figure S3B

	si-NC			si-circTFRC#1			si-circTFRC#2		
***TFRC* mRNA**	1.011619	0.874583	1.130269	0.848704	0.713672	0.591862	0.788218	0.653685	0.844791
**circ_0068631**	0.878633	0.974905	1.167428	1.051218	1.359742	1.589582	1.476436	1.207369	0.981686
